# β-Ketoenamine covalent organic framework nanoplatform combined with immune checkpoint blockade via photodynamic immunotherapy inhibit glioblastoma progression

**DOI:** 10.1016/j.bioactmat.2024.10.029

**Published:** 2024-11-07

**Authors:** Tengfeng Yan, Qiuye Liao, Zhihao Chen, Yang Xu, Wenping Zhu, Ping Hu, Si Zhang, Yanze Wu, Lei Shu, Junzhe Liu, Min Luo, Hongxin Shu, Yilei Sheng, Li Wang, Chun Xu, Chang Lei, Hongming Wang, Qingsong Ye, Li Yang, Xingen Zhu

**Affiliations:** a Department of Neurosurgery, The Second Affiliated Hospital, Jiangxi Medical College, Nanchang University, Nanchang, Jiangxi Province, 330000, China; b Jiangxi Province Key Laboratory of Neurological Diseases, Nanchang, Jiangxi Province, 330000, China; c JXHC Key Laboratory of Neurological Medicine, Nanchang, Jiangxi Province, 330000, China; d Institute of Neuroscience, Nanchang University, Nanchang, Jiangxi Province, 330000, China; e Department of Neurosurgery, Renmin Hospital of Wuhan University, Wuhan, Hubei Province, 430060, China; f Center of Regenerative Medicine, Renmin Hospital of Wuhan University, Wuhan University, 430060, Wuhan, China; g College of Chemistry and Chemical Engineering, Jiangxi Normal University, 99 Ziyang Road, Nanchang, 330022, China; h Sydney Dental School, Faculty of Medicine and Health, The University of Sydney, Sydney, NSW, 2006, Australia; i Charles Perkins Centre, The University of Sydney, Camperdown, NSW, 2006, Australia; j School of Medical Sciences, Faculty of Medicine and Health, The University of Sydney, Sydney, NSW, 2006, Australia; k College of Chemistry and Chemical Engineering and Jiangxi Provincial Key Laboratory of Functional Crystalline Materials Chemistry Nanchang University, Nanchang, 330031, China

**Keywords:** Covalent organic framework, Drug delivery, Photodynamic immunotherapy, Checkpoint blockade, Glioblastoma

## Abstract

The synergistic approach of combining photodynamic immunotherapy with endogenous clearance of PD-L1 immune checkpoint blockade therapy holds promise for enhancing survival outcomes in glioblastoma (GBM) patients. The observed upregulation of O-GlcNAc glycolysis in tumors may contribute to the stabilization of endogenous PD-L1 protein, facilitating tumor immune evasion. This study presents a pH-adapted excited state intramolecular proton transfer (ESIPT)-isomerized β-ketoamide-based covalent organic framework (COF) nanoplatform (denoted as OT@COF-RVG). Temozolomide (TMZ) and OSMI-4 (O-GlcNAc transferase inhibitor) were integrated into COF cavities, then modified on the surface with polyethylene glycol and the rabies virus peptide RVG-29, showing potential for sensitizing TMZ chemotherapy and initiating photodynamic therapy (PDT). By inhibiting O-GlcNAc and promoting lysosomal degradation of PD-L1, OT@COF-RVG enhanced the effectiveness of immune checkpoint blockade (ICB) therapy. Additionally, treatment with OT@COF-RVG led to a notable elevation in reactive oxygen species (ROS) levels, thereby re-establishing an immunostimulatory state, inducing immunogenic cell death (ICD). In summary, our research unveiled a correlation between O-GlcNAc in GBM and the evasion of immune responses by tumors, while showcasing the potential of OT@COF-RVG in reshaping the immunosuppressive microenvironment of GBM and offering a more effective approach to immunotherapy in clinical settings.

## Highlights

• **Nanoplatform:** β-ketoamide covalent organic framework acting as both a drug carrier and a type I photosensitive drug.
• **Drug Release:** Dual response of pH and light stimulation.
• **Photodynamic immunotherapy:** Induced immunogenic death and inhibited endogenous PDL-1 production by down-regulation of O-GlcNAc.
• **Treatment:** Inhibiting malignant progression of glioblastoma and inducing infiltration of CD8^+^T cells in the tumor area.

## Graphical abstract

A pH-adapted excited state intramolecular proton transfer-isomerized β-ketoamide-based covalent organic framework (COF) nanoplatform (denoted as OT@COF-RVG) has been developed. The platform enhances the effectiveness of immune checkpoint blockade therapy by inhibiting O-GlcNAc, promoting lysosomal degradation of PD-L1, and increasing systemic reactive oxygen species levels, thereby re-establishing an immunostimulatory state and inducing immunogenic cell death.

**Figure:**
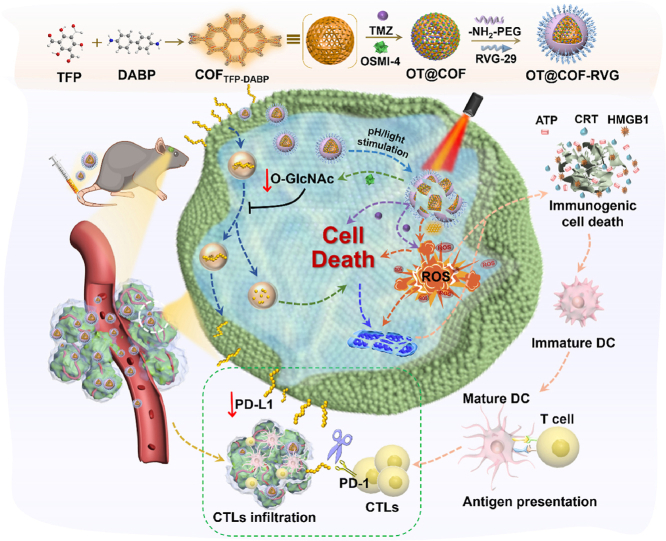

## 1 Introduction

Glioblastoma (GBM) is a prevalent primary malignant brain tumor in the adult population [1]. Despite the standard treatment protocol for GBM consisting of maximal surgical resection, postoperative radiotherapy, and adjuvant chemotherapy, patients with GBM face a grim prognosis [2]. Programmed death ligand 1 (PD-L1) and its corresponding receptor programmed cell death 1 (PD-1) play a crucial role in T cell-mediated immunity against tumors [3]. Thus, the immune checkpoint blockade (ICB) therapy utilizing anti-PD-1/PD-L1 antibodies has recently gained recognition as a successful treatment option for various tumors [4,5]. Nonetheless, GBM is often referred to as a “cold tumor” due to its tumor microenvironment (TME) exhibiting limited cytotoxic T cell infiltration, leading to suboptimal responses to ICB therapy [[6], [7], [8]]. The conventional chemotherapy drug temozolomide (TMZ) induces nucleotide mismatch and promotes the cell cycle arrest in the G2/M phase, leading to cell death of GBM cells. However, as the first-line postsurgery regimens for GBM patients, the clinical effect is very limit [9,10]. Consequently, the identification of additional molecular targets and the development of novel treatment agents to enhanced combination immunotherapy approaches to address the immune-heterogeneous TME in GBM poses a pressing challenge in the field of treatment.

The expression of PD-L1 on the tumor cells surface plays a crucial role in inducing immune escape [11]. However, PD-L1 can be internalized and transported to the cell surface through endosomes, where it undergoes recycling [12]. Recent research has shown that post-translational modifications, such as ubiquitination or palmitoylation, can lead to the degradation of PD-L1 in the lysosome [13,14]. Furthermore, the regulation of lysosomal degradation of PD-L1 can be influenced by O-linked N-acetylglucosamine (O-GlcNAc) modification of hepatocyte growth factor-regulated tyrosine kinase substrate (HGS), a crucial element in the endosomal sorting process [15]. Thus, the inhibitor of O-GlcNAc, such as OSMI-4, is expected to a potential treatment target for GBM patients [16]. Additionally, the outcomes of a phase 1 clinical trial investigating the combination of anti-PD1 monoclonal antibody pembrolizumab with chimeric antigen receptor (CAR) T cell therapy did not demonstrate significant advantages for patients with GBM [7]. The findings from clinical trials and molecular biology research suggest that the efficacy of anti-PD-L1 monoclonal antibodies in addressing current challenges is limited, necessitating the exploration of alternative therapeutic approaches.

The phenomenon of immune-competent cell death (ICD) involves the capacity of tumor cells to transition into an inflammatory immune-competent state in response to internal or external triggers, leading to enhanced infiltration of cytotoxic T lymphocytes (CTLs) within TME [17,18]. In the course of the ICD process, tumor cells release a variety of damage-associated molecular patterns (DAMP) molecules, such as adenosine triphosphate (ATP), calreticulin (CRT), and high mobility group box 1 (HMGB1), which have the capacity to facilitate the maturation of dendritic cells (DCs) and initiate sustained anti-tumor immune responses [19]. Recent research has indicated that the simultaneous administration of immunotherapy and chemotherapy can enhance the infiltration of CTLs in the TME, resulting in the conversion of immunologically “cold tumors” to “hot tumors” [20].

The integration of ICD and chemotherapy with nanoparticles carriers has shown effectiveness in reversing the immunosuppressive tumor microenvironment and enhancing the efficacy of ICB therapy [21]. The advancement of engineered nanoparticles for multi-target or multi-modal treatment of GBM is increasingly acknowledged for its potential clinical utility. Covalent organic frameworks (COF) are a unique category of crystalline porous polymer materials distinguished by their covalent bonding of small molecules and remarkable optical characteristics [22]. The material has attracted considerable attention because of its distinctive features, such as a substantial specific surface area, consistent pore channels, readily modifiable structure, exceptionally low density, and superior thermal and chemical stability [23]. Photodynamic therapy (PDT) is a photochemical treatment modality that entails the activation of intracellular photosensitizers through specific excitation light, resulting in the transformation of tumor molecular oxygen into reactive oxygen species (ROS) and consequent cell demise [24,25]. The effectiveness of PDT in managing GBM may be constrained by a singular treatment modality, however, a multifaceted strategy incorporating chemotherapy agents and targeted molecules is currently under investigation as a potentially viable solution.

Capitalizing on these properties, this study presents a pH-adapted Excited state intramolecular proton transfer (ESIPT)-isomerized β-ketoamide-based covalent organic framework nanoplatform (OT@COF-RVG) to address challenges in GBM treatment. ESIPT is a phenomenon in photophysical processes where protons transfer from one location to another within a molecule. As in the case of β-ketoamide-based covalent organic frameworks it is manifested as a proton transfer from the hydroxyl group to the neighboring nitrogen atom. Especially under acidic conditions, where the nitrogen sites in the β-ketoenamine structure undergo protonation [26]. After trigger by light, ESIPT induced photoisomerization can locally generate branched chains connected by alcohol imine bonds, reconstructing a new donor acceptor system with ketoenamine bond branches, which is beneficial for extending the charge separation lifetime and generating ROS [27]. These properties make pH and light ideal for modulating the photodynamic properties of β-ketoamide-based covalent organic frameworks. After loading the drugs TMZ and OSMI-4, to improve its blood circulation half-life and enhance blood-brain barrier (BBB) penetration, the OT@COF nanoplatform is surface-modified with polyethylene glycol (PEG) and rabies virus glycoprotein-29 (RVG-29). Upon light activation, the nanoplatform generates ROSand releases TMZ and OSMI-4 in tumor site. On the one hand, the ROS facilitate the maturation of DCs and initiate sustained anti-tumor immune responses via promoting tumor releasing a variety of DAMP molecules to trigger ICD. Then, matured DC leads to the increasing number of INF-γ^+^ CD8^+^ T cells level, which converts the “cold tumor” into “hot tumor”. On the other hand, the introduction of OSMI-4 intensifies the chemotherapy effect of TMZ via inhibiting endogenous PD-L1 expression. Additionally, we demonstrate that the nanoplatform loaded with OSMI-4 and TMZ (OT@COF-RVG) show to a higher survival compared to merely loaded with TMZ (T@COF-RVG). In conclusion, this nano strategy integrating chemotherapy, PDT, ICD and ICB provides an effective therapeutic option for GBM patients.

## 2 Results and discussion

### 2.1 Fabrication and characterization of rabies virus peptide RVG-29 camouflaged nanomedicines with high TMZ and OSMI-4 loading

**Fig. 1:**
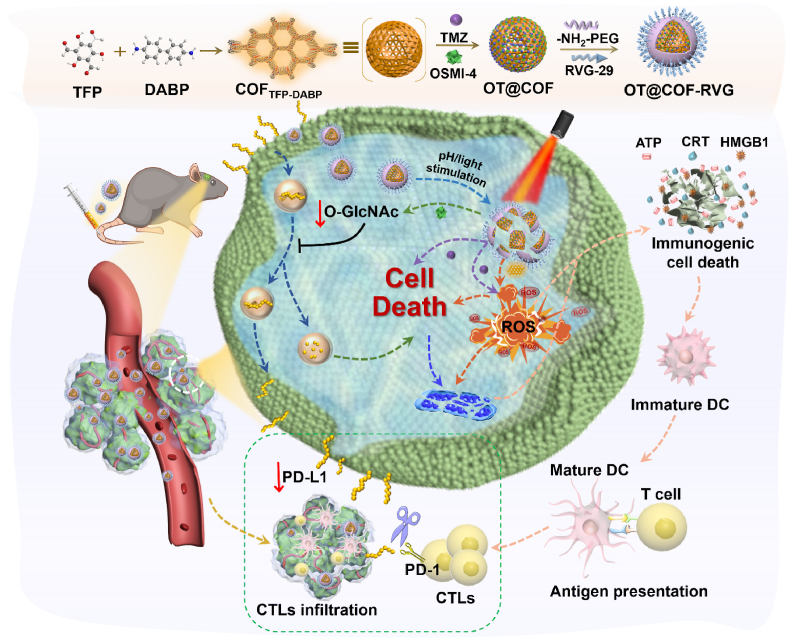
Schematic illustration. Schematic of the synthesis of OT@COF-RVG. The illustration of mechanism of OT@COF-RVG-mediated immune checkpoint blockade via photodynamic immunotherapy inhibit GBM progression.

**Fig. 2:**
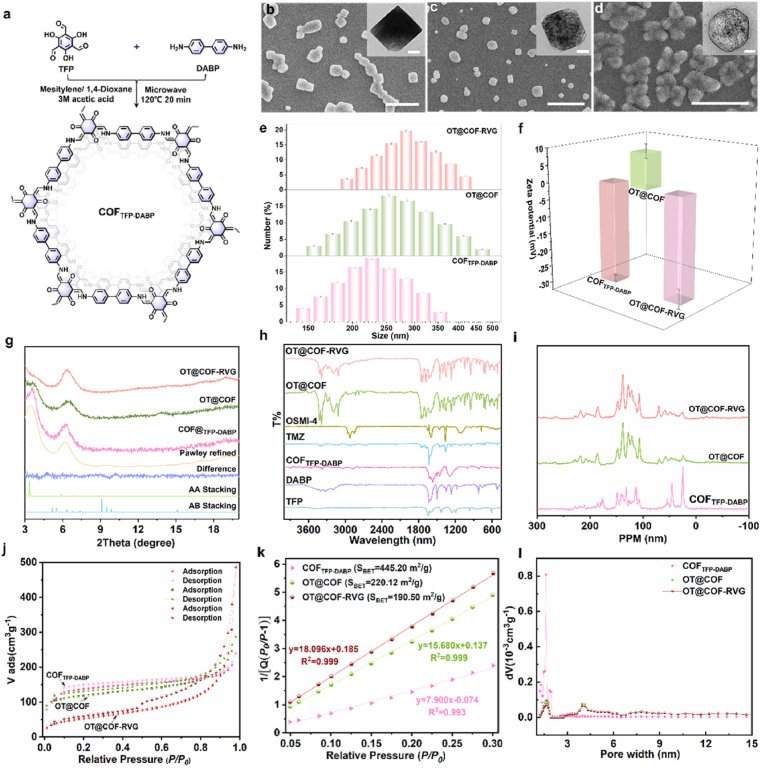
Material characterization. (a) Scheme of the synthesis process of COF_TFP-DABP_. SEM and TEM (insert) images of (b) COF_TFP-DABP_, (c) OT@COF, (d) OT@COF-RVG (SEM bar: 1 μm, TEM bar: 0.1 μm). (e) Diameter distribution of COF_TFP-DABP_, OT@COF and OT@COF-RVG measured using dynamic light scattering. (f) Zeta potential of COF_TFP-DABP_, OT@COF and OT@COF-RVG (n = 4). (g) PXRD patterns of COF_TFP-DABP_, OT@COF and OT@COF-RVG; Pawley refined with a minimum difference, simulated PXRD patterns for AA stacking and AB stacking of COF_TFP-DABP_. (h) FTIR spectra of TFP, DABP, COF_TFP-DABP_, TMZ, OSMI-4, OT@COF and OT@COF-RVG. (i) solid-state ^13^C NMR spectra of COF_TFP-DABP_, OT@COF and OT@COF-RVG. (j) The N_2_ adsorption/desorption, (k) the linear fit plot of the BET equation and (i) the pore distribution of COF_TFP-DABP_, OT@COF and OT@COF-RVG.

The artificial drug loaded nanoplatforms (OT@COF-RVG) are readily fabricated via three steps as schematically illustrated in Fig. 1. Initially, a framework network (COF_TFP-DABP_) is formed through ordered covalent connections between TFP and DABP building blocks, as shown in the chemical structure of Fig. 2a. Subsequently, TMZ and OSMI-4 are encapsulated within COF_TFP-DABP_ to form OT@COF. Finally, amino functionalized polyethylene glycol (-NH_2_-PEG) is applied as a coating, followed by the grafting of rabies virus peptide RVG-29 onto -NH_2_-PEG to form OT@COF-RVG. The scanning electron microscopy (SEM) image of COF_TFP-DABP_ reveals cube morphologies (Fig. 2b) that remain unchanged after the co-loading process (Fig. 2c). However, after the surface modification in the third step, the nanoparticles exhibit a rougher surface and blurred boundaries (Fig. 2d). The transmission electron microscopy (TEM) images in Fig. 2b–d shows that the nanoparticles become elliptical, with fuzzy boundaries and increased sizes following drug loading and peptide modification. Additionally, dynamic light scattering (DLS) measurements (Fig. 2e) indicate a significant increase in the hydrodynamic diameter of OT@COF-RVG after these modifications. Furthermore, the zeta potential of OT@COF shifts from positive to negative after the RVG-29 modification, confirming the success of the peptide grafting (Fig. 2f). Powder X-ray diffraction (PXRD) patterns revealed distinct peaks characteristic of β-Ketonamine linked crystalline porous frameworks (Fig. 2g). COF_TFP-DABP_ exhibited peaks at 3.35° and 5.82°, corresponding to the (100) and (110) facets [28], respectively (Fig. 2g). Structural reconstruction with AA stacking mode followed by Pawley refinement yielded unit cell parameters of *a=b=*30.2 Å*, c=*3.4 Å; *α* = 90°, *β* = 90° and *γ* = 120° (Table S1). Drug loading and surface peptide targeted modification reduce the crystallinity of COF_TFP-DABP_ (Fig. 2g). The amount of TMZ and OSMI-4 encapsulated in OT@COF-RVG was determined using high performance liquid chromatography (HPLC), the chromatographic conditions are shown in load efficiency (LC%) calculation in “Methods”, the results showed that the LC% of TMZ amounted to 62.1 % and that of OSMI-4 was 76.9 % (Figs. S1 and 2).

The linkage and skeleton structures were unambiguously characterized by various analytical methods. Fourier transform infrared (FT-IR) spectroscopy revealed characteristic vibration bands of the hydra-zone, imine and vinylene linkages. FTIR spectra of COF_TFP-DABP_, OT@COF and OT@COF-RVG showed characteristic C=C and C-N stretching frequencies at 1569 and 1250–1257 cm^−1^, respectively (Fig. 2h) [29]. ^13^C solid state NMR showed characteristic exocyclic C=C signals at 103–106 ppm, and the signature C=O signals were observed at 184 ppm arising from irreversible enol to keto tautomerism (Fig. 2i) [30]. The specific surface area and permanent porosity of COF_TFP-DABP_, OT@COF, OT@COF-RVG were tested using N_2_ adsorption desorption isotherms. As shown in Fig. 2j, COF_TFP-DABP_ exhibited type II reversible isotherms and formed into type IV reversible isotherms with drug loading and targeting modifications, indicating the emergence of a distinct mesoporous structure [31]. The specific surface area gradually decreases from 445.20 m^2^ g^−1^ to 190.5 m^2^ g^−1^ (Fig. 2k), and the reduced pore size distribution of 8–15 Å size indicates that the drug has been loaded successfully (Fig. 2l, Fig. S3).

### 2.2 pH responsive assist ESIPT of keto-enamine-linked COFs generates ROS

**Fig. 3:**
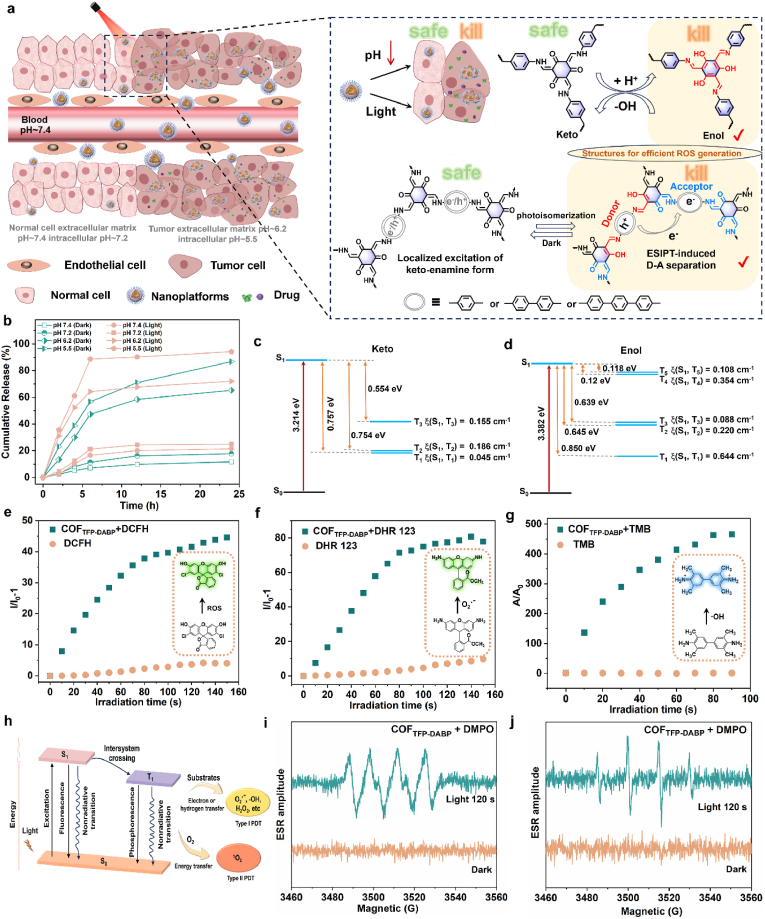
Type-I photosensitizer OT@COF-RVG via pH/light-responsive drug release. (a) Schematic diagram of the drug release mechanism of OT@COF-RVG under acidic TME stimulated by light, (b) Plot of drug release profiles of OT@COF-RVG under Dark and light conditions at different pH physiological conditions. The calculated energy level diagram between singlet and triplet states of (c) Keto and (d) Enol. (e) ROS generation of COF_TFP-DABP_ (10 μg/mL) upon light irradiation using DCFH (100 μM) as an indicator. (f) ROS generation of COF_TFP-DABP_ (10 μg/mL) upon light irradiation using DHR123 (100 μM) as an indicator. (g) Absorbance decay of TMB (100 μM) in the absence and presence of COF_TFP-DABP_ (10 μg/mL) under light irradiation. The insets in Fig. 3e–g shows the structure of the corresponding probes as well as the structural changes when indicated. The white light irradiation power is 26 mW cm^−2^. (h) Schematic illustration for the basic process of Type-I and Type-II PDT. Light exposure excites a photosensitive molecule from the ground singlet state (S_0_) to an excited singlet state (S_1_), wherein the molecule undergoes intersystem crossing to an excited triplet state (T_1_) and then forms radicals through electron or hydrogen transfer either a type I reaction or, more likely, a type II one by directly transferring its energy to the surrounding ^3^O_2_ for the generation of cytotoxic ^1^O_2_. ESR spectra to detect (i) ^.^OH and (j) O_2_^.-^ from COF_TFP-DABP_ under irradiation, using DMPO as the spin-trap agent.

Acidic TME induced by massive anaerobic glycolysis is one of the important features of malignant tumors and an important factor in inducing tumorigenesis, metastasis, and the development of drug resistance [32]. Compared to the pH of normal cells and tissues (7.2–7.4), the pH within tumor cells and tissues is low (5.5–6.2). In response to the aforementioned characteristics of the tumor microenvironment, we present a keto-enolamine-linked COF_TFP-DABP_ exhibiting ESIPT properties (Fig. S4a). As shown in Fig. 3a and Figs. S4a–c, at acidic pH, the structure is predominantly in the enol form. Under light irradiation, the keto-enolamine structure gradually transforms into a photoisomer that contains a mixture of keto and enol forms. This partially enolized photoisomer exhibits extended π-conjugation and increased electron density. Such structural changes facilitate the reconstruction of a spatially separated electron donor-acceptor (D-A) configuration, where the enol-imine linked branch serves as the electron donor, while the keto-enolamine linked branch functions as the electron acceptor [26,27]. This configuration promotes light-induced charge transfer between the two neighboring branches, characterized by a prolonged lifetime (Fig. S4b). Under acidic conditions in the tumor region, COF_TFP-DABP_ protonation increased, and the structure was dominated by the Enol conformation to release the drugs. It tends to have a more stable Keto structure in the neutral or alkaline regions of normal tissue cells and has a high safety profile. As shown in Fig. 3b, the drug release was as high as 87 % when the pH was as low as 5.5, and the drug release efficiency was further increased to 94 % under light stimulation. In contrast, the drug release efficiency was no more than 21 % in normal cells and tissues at pH 7.2–7.4.

COF_TFP-DABP_ was observed to produce a significant amount of reactive oxygen species (ROS) when exposed to light. White light covers a wide spectrum and is used as the light source for triggering photodynamic therapy in this work, the white light irradiation power is controlled at 26 mW cm^−2^. The total ROS-generating capacity was measured using 2′,7′-dichlorofluorescein (DCFH) as an indicator, and the fluorescence intensity of DCFH increased by up to 32-fold within 90 s of light exposure (Fig. 3e). The O_2_^.-^ probe dihydrorhodamine 123 (DHR 123), ^.^OH probe 3,3′,5,5′-Tetramethylbenzidine (TMB) and singlet oxygen (^1^O_2_) fluorescence probe 9,10-Anthracenediyl-bis(methylene) dimalonic Acid (ABDA) were utilized to identify the species of ROS generated by COF_TFP-DABP_. The fluorescence of DHR 123 (Fig. 3f) and the UV-absorption intensity of TMB (Fig. 3g, Fig. S4) under 90 s light irradiation reached more than 12 and 93 times of that before irradiation, respectively, while the ABDA only did not show significant changes (Figs. S5 and S6), thus indicating that COF_TFP-DABP_ produces O_2_^.-^ and ^.^OH with a higher efficiency [33]. To further confirm the generation of O_2_^.-^ and^.^OH, electron spin resonance (ESR) measurements were performed in organic and aqueous phases, respectively, using 5,5 dimethyl-1-pyrroline-n-oxide (DMPO) as spin trapping agent. In the presence of COF_TFP-DABP_ and DMPO, obvious O_2_^.-^ (Fig. 3i) and ^.^OH (Fig. 3j) ESR signals appeared in the ESR spectra after 120 s of light irradiation [34]. However, the ESR signal of ^1^O_2_ did not change significantly before and after light irradiation (Fig. S7). Hence, COF_TFP-DABP_ could act as a Type-I photosensitizer to induce Type-I PDT.

As shown in Fig. 3h, type I photosensitizers mainly react with the surrounding matrix through electron or hydrogen transfer after photoexcitation, producing free radical species such as O_2_^•−^and ^.^OH. Type II photosensitizers transfer the energy of the excited state directly to oxygen through an energy transfer mechanism, generating ^1^O_2_ [35]. From the perspective of the mechanism of ROS production, ESIPT-induced proton and electron transfer that occurs in COF_TFP-DABP_ in response to pH and light stimulation is more likely to generate type I ROS (Fig. S4). In addition, the distribution of photo generated electrons and holes in the incision model was studied using Multiwfn [36]. After ESIPT induced partial photoisomerization of ketone enamine, the hybrid bonds formed altered the electronic properties of the local skeleton, promoting the cross edge transfer of photo generated electrons and resulting in significant charge separation. According to the hole electron theory, the class charge transfer mode can be quantified by the S/D value [37,38], where S represents the overlap integral of the hole electron distribution and D represents the distance between the centers of mass of the hole electron. The smaller the S/D value, the higher the efficiency of photoelectron hole separation, as shown in Fig. S4d shows that Enol and Keto have distinct charge transfer characteristics.

To further elucidate the important role of enol isomerization in generating ROS, we conducted energy level calculations for both the singlet and triplet states of keto (Fig. 3c) and enol (Fig. 3d). It was found that enol exhibits an additional ISC channel with an energy gap as low as 0.118 eV. Spin-orbit coupling (SOC) is identified as the mechanism responsible for enhancing intersystem crossing (ISC). The calculated SOC value between S_1_ and T_5_ of enol is 0.108 cm^−1^, which is smaller than that of other ISC channels. Therefore, the effective ISC process from S_1_ to T_5_ should be the reason for the superior ROS-generating ability of the enol structure [39]. To illustrate the generality of this conclusion, COF_TFP-DABP_, COF_TFP-PDA_, COF_TFP-DATP_ and the model compound COF_TFP-AB_ with similar structures were synthesized using TFP as a backbone, and COF_TFB-DABP_ without the ketoenolamine-enol structure was used as a comparative material (Figs. S8–10, Tables S2–4). Various ROS probes (DHR123, TMB, ABDA) were employed under specific conditions (Figs. S11–14), revealing that COFs with the β-ketoenamine-based structure have a notable ability to produce O_2_^.-^ and ^.^OH, while the comparison material showed minimal production. The β-ketoenamine-based COFs, serving as a Type-I photosensitizer, represents a novel proposal and discovery. In addition, two models were employed to assess light penetration and the capability of COF_TFP-DABP_ to generate ROS upon light irradiation, as illustrated in Fig. S15. The penetration of light into the skin diminishes significantly with the increasing number of skin layers, and the depth of light penetration directly influences the efficiency of COF_TFP-DABP_ in producing ROS within tissues. Consequently, COF_TFP-DABP_ can effectively generate ROS to a specific depth within the tissue.

### 2.3 Tumor-specific accumulation property of COF-RVG

**Fig. 4:**
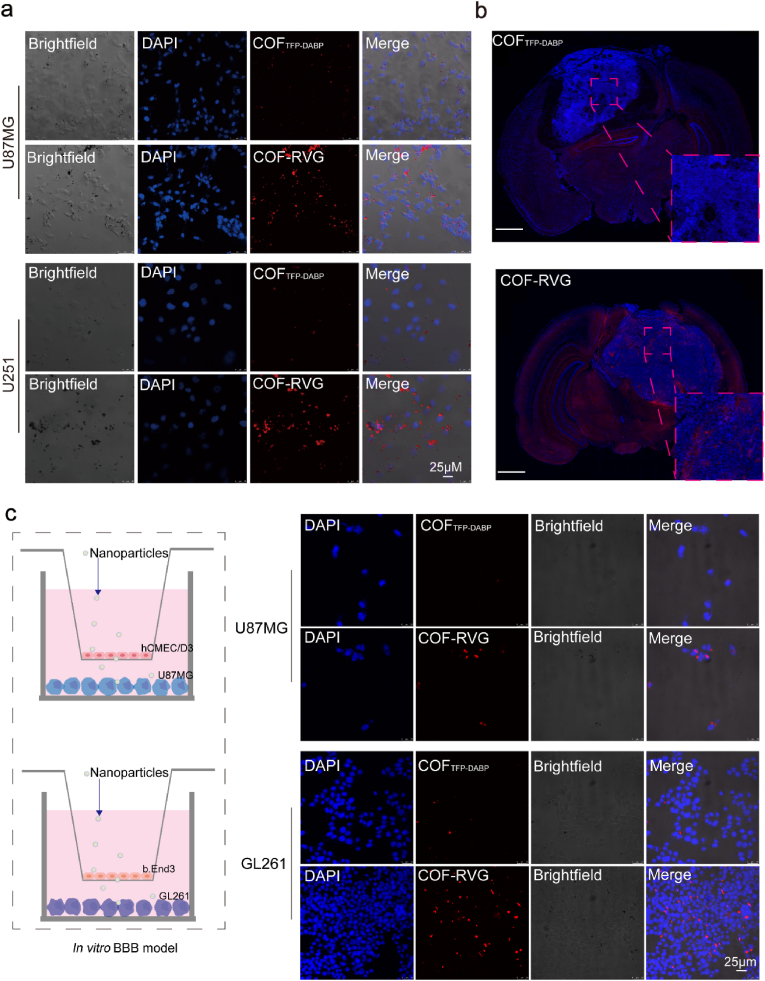
Enrichment of COF-RVG in tumor models. (a) Representative immunofluorescence images of GBM cells incubated with nanoparticles. (b) Representative immunofluorescence images of tumor sections after treaments. (c) The Schematic illustration of BBB in vitro cell model and representative immunofluorescence images of GBM cells incubated with nanoparticles (30 μg/mL). Cell nucleus was stained with DAPI (blue).

The efficacy of drug therapy for GBM hinges upon the ability of drugs to traverse the BBB and accumulate at the tumor site. The RVG29 peptide exhibits a specific affinity for the nicotinic acetylcholine receptor (nAchR) present on the outer membrane of GBM cells and brain microvascular endothelial cells, facilitating its uptake by GBM cells [40,41]. The co-incubation of U87MG or U251 cells with 30 μg/mL COF_TFP-DABP_ and COF-RVG for a duration of 4 h demonstrated a notable enhancement in the internalization of in GBM cells, particularly with the addition of RVG peptides (Fig. 4a, Fig. S16). This observation was supported by immunofluorescence analysis of tissue sections, revealing a significant increase in red fluorescence within the core region of the tumor in mice treated with COF-RVG (Fig. 4b). Cell uptake was further analyzed using CLSM. The findings indicate that the internalization of COF-RVG is significantly enhanced compared to unmodified COF_TFP-DABP_, likely due to the increased affinity between COF-RVG and nAChR receptors on GBM cells (Figs. S17–21). Additionally, the fluorescence intensity in U87MG and U251 cells exhibits a significant dependence on both time and concentration. The successful internalization of OT@COF-RVG by GBM cells is crucial for their anticancer efficacy, as evidenced by the strong fluorescence signal observed in this study.

The in vitro BBB model was developed utilizing the Transwell system, wherein a small chamber is lined with a monolayer of brain microvascular endothelial cells (hCMEC/D3 or b.End3) and a lower compartment containing GBM cells (U87MG or GL261). Following a 24-h incubation period, the fluorescence signals of GL261 or U87MG cells in the lower chamber were visualized using CLSM. Interestingly, COF-RVG added with RVG peptides exhibited significantly enhanced fluorescence intensity compared to COF_TFP-DABP_ (Fig. 4c, Fig S22). These findings suggest that RVG peptides enhance the ability of COF_TFP-DABP_ to traverse the BBB.

### 2.4 OT@COF-RVG in vitro biosafety and stimulation of ROS production

**Fig. 5:**
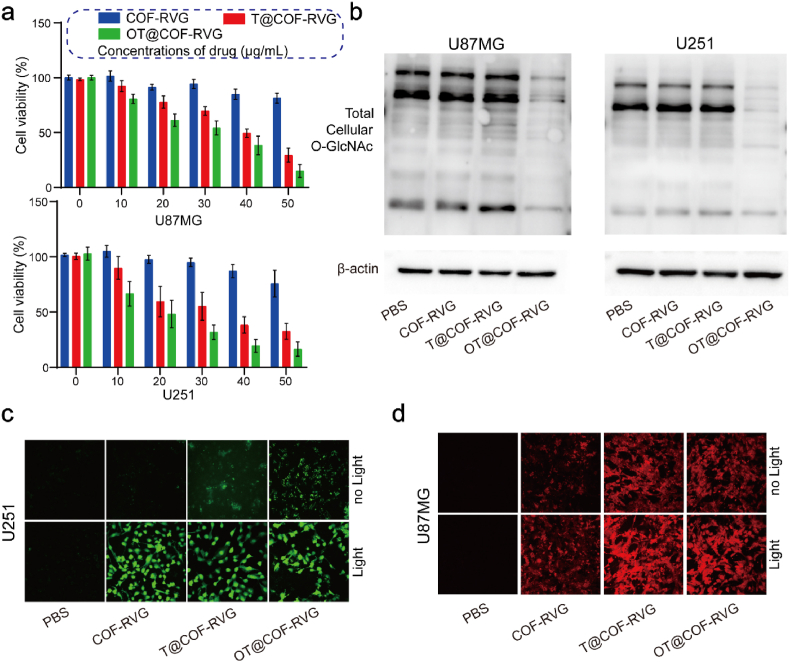
OT@COF-RVG in vitro biosafety and stimulation of ROS production. (a) Cytotoxicity analysis of CCK-8 assay (n = 3). (b) Western blot analysis of total cellular O-GlcNAcexpression in U87MG and U251 after co-incubation with different nanoparticles. (c) The representative fluorescence microscopic images of GBM cells after co-incubation with different system stained by DCFH-DA (green) and (d) DHE staining (red) with or without light. The white light irradiation power is 26 mW cm^−2^.

Prior research has demonstrated that chemotherapy has the ability to stimulate the biosynthesis pathway of hexosamine, increase O-GlcNAcylation in cancer cells, and trigger pathways associated with chemotherapy resistance [42]. OSMI-4, an inhibitor of OGT, has been shown to effectively decrease the level of O-GlcNAc in cells [43]. In this study, OSMI-4 and TMZ were encapsulated in COF-RVG. Utilizing the CCK-8 assay to assess the cytotoxicity of nanoparticles on GBM cells, it was observed that co-incubation of 30 μg/mL COF_TFP-DABP_ with GBM cells resulted in cell viability exceeding 80 %. Furthermore, the introduction of OSMI-4 notably intensified the cytotoxic effects of TMZ on GBM cells (Fig. 5a). To validate the impact of OT@COF-RVG on intracellular O-GlcNAc modification, the O-GlcNAc levels in whole cells were analyzed using Western blot. The study revealed that the introduction of OSMI-4 led to a notable decrease in O-GlcNAc levels in GBM cells (Fig. 5b, Fig S23). In order to assess the ROS generation capability of OT@COF-RVG in cells, the fluorescent probes of DCFH-DA and DHE were employed to investigate the process of ROS production in GBM cells. The results demonstrated that exposure to light significantly augmented the elevation of DCFH-DA and DHE fluorescence levels (Fig. 5c and d Fig S24), suggesting that OT@COF-RVG exhibited pronounced cytotoxic effects on GBM cells.

### 2.5 OT@COF-RVG promote ICD and cause PD-L1 down regulation

**Fig. 6:**
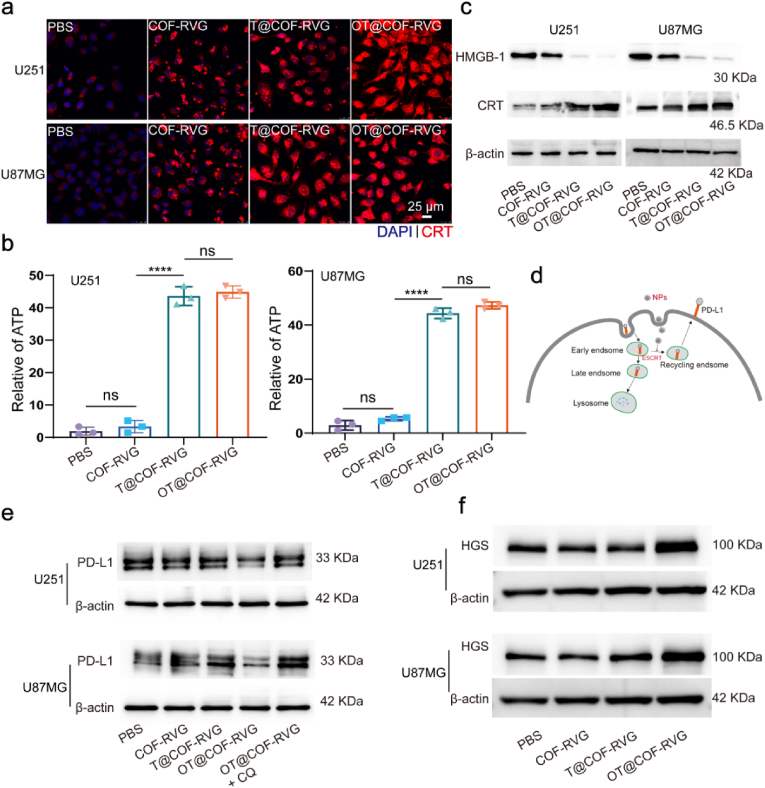
OT@COF-RVG promote ICD and cause PD-L1 down regulation. (a) Immunofluorescence staining analysis of CRT expression on GBM cells after different treatments. (b) ATP level released by cells after different treatments (n = 3). (c) Western blot analysis of CRT and HMGB-1 expression after different treatments. (d) Mechanism of O-GlcNAc modification regulating PD-L1 expression. (e–f) Western blot analysis of PD-L1 and HGS expression after different treatments. All experiments were exposed to white light. Cell nucleus was stained with DAPI (blue).

It is important to acknowledge that ROS generated during PDT have the potential to elicit cellular oxidative stress and activate ICD [44]. Furthermore, ICD plays a critical role in promoting tumor cell immunotherapy [45]. To assess the efficacy of inducing ICD in vitro and the release of DAMP molecules, the analysis of OT@COF-RVG was conducted. Following co-incubation of GBM cells with nanoparticles, it was observed that OT@COF-RVG led to a notable increase in the exposure of CRT on tumor cells compared to the control groups (Fig. 6a). The levels of ATP secretion in the cell supernatant exhibited a comparable pattern, as depicted in Fig. 6b. Moreover, the OT@COF-RVG and T@COF-RVG groups exhibited a significant increase in HMGB1 protein expression compared to the remaining groups, whereas the expression of CRT protein showed an opposite trend (Fig. 6c). The findings suggested that OT@COF-RVG effectively triggered ICD in GBM cells.

Previously documentation has shown that O-GlcNAc modification of HGS inhibits the interaction between PD-L1 and the endosomal sorting mechanism, leading to the hindered degradation of PD-L1 from early endosomal to lysosomal compartments. This ultimately results in the upregulation of PD-L1 cell membrane protein and the suppression of the anti-tumor function of T cells [15]. Thus, our hypothesis posited that OT@COF-RVG effectively suppresses PD-L1 expression through the inhibition of endosomal recycling of PD-L1 (Fig. 6d). To confirm this effect on the post-translational modification of PD-L1 protein, Western blot was employed to assess PD-L1 expression in GBM cells co-incubated with nanoparticles. The findings revealed that the introduction of chloroquine could successfully counteract the PD-L1 downregulation induced by OT@COF-RVG in a manner dependent on dosage (Fig. 6e Figs. S25–26). On the contrary, the addition of MG132 had minimal impact on the OT@COF-RVG-induced downregulation of PD-L1 (Fig. S27). Concurrently, Western blot was employed to assess the expression of HGS in GBM cells co-treated with nanoparticles, revealing a correlation with prior research indicating that reduced O-GlcNAc levels can enhance HGS expression (Fig. 6f and Fig. S25). These findings indicated that OT@COF-RVG effectively inhibits PD-L1 expression by disrupting the endosomal sorting pathway of PD-L1.

### 2.6 The OT@COF-RVG therapeutic effect in vivo

**Fig. 7:**
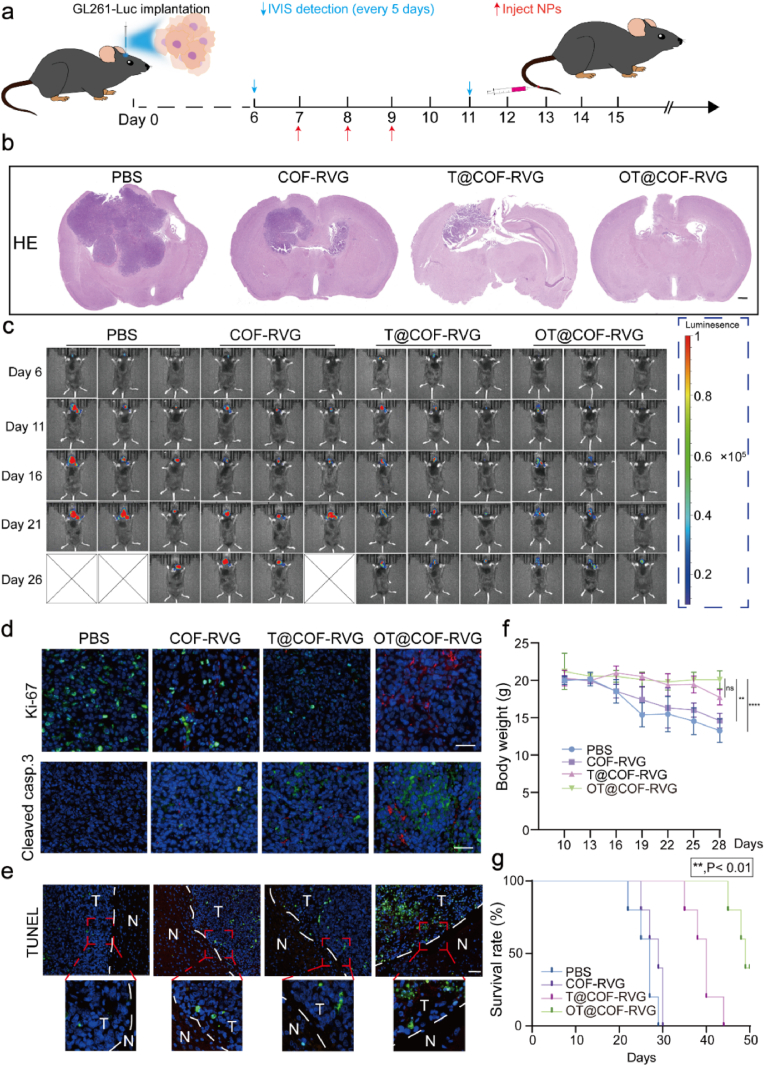
The OT@COF-RVG therapeutic effect in vivo. (a) Schematic diagram of anticancer therapy in vivo. (b) Representative brain sections of mice at termination after different treatments. (c) IVIS fluorescence imaging was utilized to observe the tumor site following various treatment modalities. (d) The fluorescence expression of Ki-67 and cleaved caspase-3 in the tumor area was assessed following various treatments. (e) The TUNEL assay was employed to assess the apoptosis of tumor cells following various treatments. (f) The study examines the variations in weight of mice with GBM in situ at various time intervals following diverse treatment regimens. (g) Survival curves of mice with intracranial GBM following various therapeutic interventions.

To further explore the anticancer properties of distinct nanoparticles group on the advancement of GBM, a murine model was developed through the intraventricular injection of luciferase-tagged GL261 cells into C57 mice. Bioluminescence imaging was conducted on the sixth day post-implantation and subsequently repeated every five days. The experimental group was administered nanoparticles treatment once daily for a period of 3 days following 7 days post tumor implantation (Fig. 7a). The white light irradiation power is 26 mW cm^−2^. The utilization of OT@COF-RVG demonstrated a notable decrease in tumor volume in comparison to the remaining groups. This finding indicated that the treatment with OT@COF-RVG effectively impeded the progression of GBM (Fig. 7b, c, and Fig. S28). Furthermore, Ki-67 and Cleaved caspase-3 were utilized for immunofluorescence assessment of tumor proliferation and apoptosis rates. The results revealed a significant decrease in Ki-67 positivity and a significant increase in Cleaved caspase-3 positivity in the OT@COF-RVG group (Fig. 7d). Then, similar outcomes were achieved through TUNEL assay, wherein the OT@COF-RVG group exhibited the highest rate of positivity, indicating that OSMI-4 may enhance the apoptotic effects of TMZ on GBM cells in an in vivo setting (Fig. 7e). Monitoring of the mice's body weights every three days, commencing ten days post-tumor implantation, revealed minimal fluctuations in the body weights of mice in the OT@COF-RVG group, contrasting with the notably rapid weight loss observed in the PBS group (Fig. 7f). Ultimately, the survival rate of mice in the OT@COF-RVG group surpassed that of other groups (Fig. 7g). These findings suggested that targeting O-GlcNAc in GBM cells may augment the efficacy of TMZ treatment for GBM.

### 2.7 Biosafety of OT@COF-RVG

In order to assess the biocompatibility of OT@COF-RVG in vivo, the nanoparticles were administered via tail vein injection to healthy mice, and subsequent examination of major organs (heart, liver, spleen, lung, and kidney) was conducted using H&E staining. Following three days of daily tail vein injections of nanoparticles s or PBS, no discernible pathological abnormalities were observed in the aforementioned organs, indicating a high level of histocompatibility for OT@COF-RVG (Fig. S29). Moreover, the haemolysis rate of OT@COF-RVG was found to be low, indicating the absence of haemolysis (Fig. S30). Subsequently, an evaluation of blood haematological and biochemical parameters in mice was conducted, focusing primarily on major liver and kidney function indices (Figs. S31–32). The findings revealed no statistically significant differences in the levels of key parameters such as blood urea nitrogen (BUN), hemoglobin (HGB), creatinine (CR), etc, suggesting that the mice exhibited tolerance to the administration of these nanoparticles. Therefore, it can be concluded that OT@COF-RVG can be safely utilized in vivo.

To investigate whether the observed anti-tumor effects were dependent on the immune system (CD8 T cells), anti-CD8α antibody was injected intraperitoneally into mice to remove lymphocytes in vivo. The results showed that CD8^+^ T cell depletion significantly impaired tumor suppression, resulting in failure to inhibit tumor growth for a short period of time and shortened the survival time of OT@COF-RVG-treated mice (Fig. S33), confirming the central role of the immune system in OT@COF-RVG therapy.

### 2.8 OT@COF-RVG activating CD8^+^T cells and down regulating PD-L1 in vivo

**Fig. 8:**
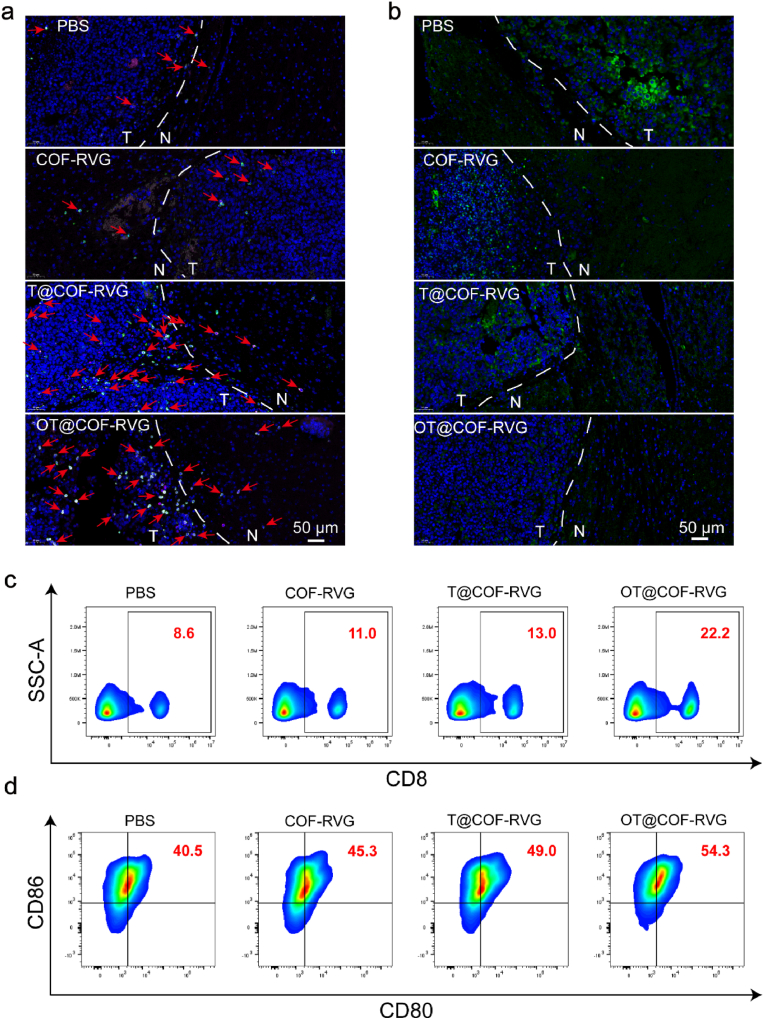
OT@COF-RVG Activating CD8^+^T cells and down regulating PD-L1 in vivo. (a) The immunofluorescence imaging was utilized to detect the expression of CD8 in the tumor area of GBM in situ mice following various treatments. (b) The assessment of PD-L1 expression within the tumor microenvironment of GBM-bearing mice following various therapeutic interventions utilizing immunofluorescence imaging. (c) Flow cytometry dot plots depicting the distribution of CD8 cells illustrate the relative abundance of CD8^+^ T cells within the lymphocyte population in the spleen following various experimental interventions. (d) Flow cytometry dot plots displaying CD11c and CD86 expression represent the proportions of dendritic cells in mouse tumor-draining lymph node (TDLN) following various treatments.

In the context of therapy for GBM, the induction of a robust ICD effect within the GBM in situ is essential for enhancing the infiltration of CD8^+^ T cells and subsequently converting the immunologically “cold” microenvironment [45,46]. To investigate this phenomenon, we evaluated the expression of CD8 within GBM tumor regions following the administration of nanoparticles through immunofluorescence staining of brain sections obtained from mice bearing transplanted tumors. Our findings revealed a significant upregulation of CD8 expression within the GBM tumor region subsequent to treatment with OT@COF-RVG and T@COF-RVG (Fig. 8a–c Figs. S34–35). The findings from the in vitro experiments indicated that OSMI-4 effectively reduced the expression of endogenous PD-L1, leading to an enhancement in ICB therapy. Subsequent immunofluorescence analysis of mouse tissue sections revealed a significant decrease in PD-L1 expression within the GBM tumor region following treatment with OT@COF-RVG in situ transplanted tumor mice (Fig. 8b). We further determined the secretion of cytokines in tumor tissues by enzyme-linked immunosorbent assay (ELISA). Compared with other groups, the secretion of pro-inflammatory-related factors IFN-γ, TNF-α, IL-6, IL-12, and CXCL-10 was significantly increased in the group treated with OT@COF-RVG, while the secretion of anti-inflammatory-related factor IL-10 was significantly decreased (Fig. S36), suggesting immune activation in the tumor region. Mature dendritic cells (mDCs) serve as a notable indicator for the augmentation of T cell-mediated anti-tumour immunity via proficient presentation of tumour antigens [47,48]. Consequently, we conducted an examination of the expression of mDCs biomarkers CD80 and CD86 in tumors post-treatment with various nanoparticles using flow cytometry. The flow cytometry analysis demonstrated a significantly elevated expression of CD80 and CD86 in the tumor regions of mice with in situ transplanted tumors treated with OT@COF-RVG and T@COF-RVG compared to other treatment groups (Fig. 8d and Figs. S37–38). To assess the immune function of OT@COF-RVG-triggered T cells, we monitored the proportion of IFN-γ-expressing CD8^+^ T cells in the spleen by flow cytometry analysis. After treatment with OT@COF-RVG, the proportion of IFN-γ^+^CD8^+^ T cells increased to a high level of approximately 5.66 % (Figs. S39–40), suggesting that T-cell immunity with tumor-killing function was induced. Meanwhile, the proportion of splenic immunosuppressive regulatory T cells (Tregs, CD4^+^Foxp3^+^) was significantly decreased in the OT@COF-RVG group compared with the other groups (Figs. S41–42). In addition, anti-inflammatory M2-like tumor-associated macrophages (TAMs, CD206^hi^CD11b^+^F4/80^+^) were significantly decreased and pro-inflammatory M1-like TAMs (CD86^hi^CD11b^+^F4/80^+^) were significantly increased in the OT@COF-RVG treatment group (Figs. S43–46). And, we explored other anti-tumor immune cells in the tumor region, such as NK cells (CD45^+^NK1.1^+^CD3^−^), and the results showed that the OT@COF-RVG-treated group had a significantly higher content of NK cells compared with the other groups (Figs. S47–48). These results indicated that OT@COF-RVG induced a robust immune response. In addition, we monitored the levels of CD8^+^ effector memory T cells (T_EM_, CD3^+^CD8^+^CD62L^low^CD44^hi^) in mice after different treatments. The results show that the proportion of T_EM_ in the spleen was dramatically increased after treatment with OT@COF-RVG (Fig. S49-50**)**, indicating that OT@COF-RVG established a durable immune memory. In order to examine the impact of human-derived T cells on GBM tumor cells treated with OT@COF-RVG, a co-culture of T cells and GBM cells was established, followed by live-dead cell staining conducted after 24 h of incubation. The results of the live-dead cell staining experiments demonstrated that OT@COF-RVG and T@COF-RVG exhibited the most potent cytotoxic effect on GBM cells. Notably, the cytotoxic effect of OT@COF-RVG was significantly enhanced following exposure to light (Fig. S51**)**. These findings suggested that OT@COF-RVG has the capacity to induce a robust immune response and suppress PD-L1 expression.

## 3 Conclusions

In summary, we have developed an artificial nanocomponent to enhance the efficacy of ICD based on PDT response. This nanocomponent contained an inhibitor (OSMI-4) and a chemotherapy drug (TMZ). The immunosuppressive of the tumor microenvironment in GBM hinders the effectiveness of ICB therapy for patients. Our study demonstrated that these COF nanoparticles possess the capability to produce Type-I ROS, leading to a substantial increase in ROS levels. This heightened ROS production within the tumor microenvironment can result in ICD and alteration of the immune-suppressive milieu. Furthermore, our findings showed a notable upregulation of CD8 expression in brain tumor slices from mice with orthotopic GBM. Our research exemplifies the efficacy of inducing ICD, reducing endogenous PD-L1, and utilizing a combination of nanoparticles and chemotherapy agents. It underscores the significance of artificial assemblies in modulating the immune microenvironment of tumors. Nonetheless, the heterogeneity of the tumor microenvironment necessitates further clinical trials and fundamental research to elucidate the temporal evolution of the tumor microenvironment in GBM patients following temozolomide treatment. The implementation of administration through different temporal windows may hold greater significance for patients undergoing combined GBM chemotherapy and immunotherapy.

## CRediT authorship contribution statement

**Tengfeng Yan:** Writing – original draft, Methodology, Funding acquisition, Conceptualization. **Qiuye Liao:** Methodology, Investigation. **Zhihao Chen:** Validation, Methodology. **Yang Xu:** Formal analysis. **Wenping Zhu:** Visualization, Project administration. **Ping Hu:** Formal analysis. **Si Zhang:** Methodology. **Yanze Wu:** Methodology. **Lei Shu:** Conceptualization. **Junzhe Liu:** Visualization, Formal analysis. **Min Luo:** Investigation. **Hongxin Shu:** Validation, Formal analysis. **Yilei Sheng:** Formal analysis. **Li Wang:** Supervision. **Chun Xu:** Writing – review & editing. **Chang Lei:** Writing – review & editing, Validation. **Hongming Wang:** Conceptualization, Formal analysis. **Qingsong Ye:** Supervision. **Li Yang:** Writing – review & editing, Funding acquisition. **Xingen Zhu:** Supervision, Methodology.

## Ethics approval and consent to participte

All animal handling protocols and experiments were approved by Institutional Animal Care and Use Committee of Nanchang University (NCULAE-20221031054).

## Declaration of competing interest

Qingsong Ye is an editorial board member for Bioactive Materials and was not involved in the editorial review or the decision to publish this article. The authors declare that they have no known competing financial interests or personal relationships that could have appeared to influence the work reported in this paper.
