# Supplementary material for: β-Ketoenamine covalent organic framework nanoplatform combined with immune checkpoint blockade via photodynamic immunotherapy inhibit glioblastoma progression
Source: Bioact Mater. 2024 Nov 7;44:531–43. doi: 10.1016/j.bioactmat.2024.10.029 (PMC11583667; doi:10.1016/j.bioactmat.2024.10.029)
Supplement: Multimedia component 1 [file mmc1.doc]

**Supplementary data**

**β-Ketoenamine Covalent Organic Framework Nanoplatform Combined with Immune Checkpoint Blockade via Photodynamic Immunotherapy Inhibit Glioblastoma Progression**

**Methods**

**Materials:** All chemicals and solvents used for synthesis were commercially available and directly used without further purification. 1,3,5-Triformylbenzene (TFB), 1,3,5-Triformylphloroglucinol (TFP), aminobenzene (AB), p-Phenylenediamine (PDA), 4,4-Diaminobiphenyl (DABP), 4,4''-Diamino-p-terphenyl (DATP) were obtained from Jilin Chinese Academy of Sciences-Yanshen Technology Co., Ltd. (Changchun, China). 3,3',5,5'-Tetramethylbenzidine (TMB), Glacial acetic acid (CH_3_COOH), sodium dihydrogen phosphate (NaH_2_PO_4_•2H_2_O), disodium hydrogen phosphate (Na_2_HPO_4_•12H_2_O) were purchased from Aladdin Bio-Chem Technology Co., Ltd (Shanghai, China). PEG300 were purchased from Beijing Solarbio science&technology Co. Ltd. (Beijing, China). Tetrahydrofuran (THF), ethanol and methanol were purchased from Xilong Chemical Co. Ltd. (Guangdong, China). Cell Counting Kit-8, ROS Assay Kit DCFH-DA and DHE was purchased from Nanjing Jiancheng Bioengineering Institute (Nanjing, China). Dihydrorhodamine 123 (DHR 123), 9,10-Anthracenediyl-bis(methylene)dimalonic Acid (ABDA), OSMI-4, Temozolomide (TMZ), Tween 80, DMEM, penicillin-streptomycin liquid and trypsin-EDTA solution (0.25%) were purchased from M&C Gene Technology Co., Ltd. (Beijing, China). RVG29 was purchased from Xi'an Qiyue Biotechnology Co., Ltd. (Xi'an, China). Fetal bovin serum (FBS) was purchased from Gibco (USA). All other reagents and solvents gotten from the suppliers were analytical grade and used as received.

**Characterization:** Scanning electron microscopy (SEM) was performed on HITACHI S-3400N instrument. Transmission electron microscopy (TEM) images were taken on JEOLJEM-2100 microscopes. Zeta potential and average diameter of nanoparticles were measured by NanoBrook 90PlusPALS. Fourier transform infrared spectroscopy (FTIR) were measured on a Perkin-Elmer Spectrum I spectrometer. Solid-State ^13^C CP-MAS nuclear magnetic resonance (NMR) spectroscopy were recorded on a Bruker AVANCE III spectrometer. X-ray powder diffraction analysis (PXRD) was obtained with D/Max 2500 V/PC instrument. N_2_ adsorption/desorption isothermal tests were carried out by Autosorb-iQ (Quantachrome) under a liquid nitrogen atmosphere of 77 K. Fluorescence emission spectrum was measured with a Hitachi-4700 fluorescence spectrometer. The UV-vis absorption spectrum was measured with a Shimadzu UV-2600 spectrophotometer.

**Preparation of** **COF_TFP-DABP_, COF_TFP-PDA_, COF_TFP-DATP_, COF_TFP-AB_,** **COF_TFB-DABP_:** COF_TFP-DABP_, COF_TFP-PDA_, COF_TFP-DATP_, COF_TFP-AB_ were synthesized via the Schiff-base reactions and the irreversible enol-toketo tautomerization between TFP and linkers (DABP, AB, PDA, or DATP). In short, COF_TFP-DABP_ was synthesized under microwave heating conditions by preparing in a 3:2 molar ratio solution of DABP (55 mg, 184.24, 0.3 mmol) and TFP (42 mg, 0.2 mmol) in a mixture of mesitylene/1,4-dioxane/6M acetic acid (8.4 mL, 10: 10:1). After sonication for 15 minutes, the reaction solution was subjected to three freeze-pump-thaw cycles and the thick-walled pressure-resistant bottles was turned off and heated by microwave irradiation at 120 °C with stirring for 20 min using a microwave synthesizer. Afterwards, the product was centrifugal washing with THF and acetone, respectively. Then the powder was collected and washed by employing THF as solvent using standard soxhlet method to remove any impurities adsorbed in the porous structure. After dried under vacuum at 60 °C for 24 hours, the COF_TFP-DABP_ was obtained as an orange-yellow powder in 87.3% yield based on the starting materials [1-2]. COF_TFP-PDA_, COF_TFP-DATP_ and COF_TFP-AB_ were prepared in the same way as COF_TFP-DABP_ by substituting PDA (32 mg, 108.14, 0.3 mmol), DATP (78 mg, 260.33, 0.3 mmol) and AB (27 mg, 93.13, 0.3 mmol) for DABP, the corresponding yields were 71.4%, 86.7% and 79.8%, respectively. The comparison material COF_TFB-DABP_, which does not contain enol-toketo tautomerization, then substitutes TFB (32 mg, 162.14, 0.2 mmol) for TFP. The preparation process of the above COFs and the structures of the individual COFs obtained are shown in Fig. 1a and Fig. S1a.

**Preparation of T@COF, OT@COF, COF-RVG, T@COF-RVG and** **OT@COF-RVG**

Briefly, 1 mL of TMZ (3.8 mg, 20 mM), formulated from DMSO, and OSMI-4 (2.1 mg, 3.44 mM) solutions were added to 3 mL of COF (5 mg/mL) solution in PBS (pH=7.4), respectively, and the reaction was carried out for 12 h at room temperature on a magnetic stirrer. The products were collected by centrifugation (5000 rpm for 5 min) and dialyzed for 24 h in PBS using a 3500 Da dialysis bag with dialysate changes every 6 h. The dialysis products were collected by centrifugation. Freeze-drying for 6h gave the product OT@COF, which was an orange-yellow powder with a yield of 91.8%. T@COF and O@COF were synthesized in a similar way with the addition of TMZ or OSMI-4 as required, giving yields of 89.5% and 87.6%, respectively. RVG-29 and bis-terminal amino PEG (MW 1000) were dissolved in DMSO at a molar ratio of 1:1.2. 5 mg of OT@COF was added and stirred on a magnetic stirrer for 12 h. The product was collected by centrifugation (5000 rpm for 5 min). and dialyzed in PBS using a 3500 Da dialysis bag for 24 h. The dialysate was changed every 6 h [3-4]. The dialysis product was collected by centrifugation and freeze-dried to obtain the product OT@COF-RVG with a yield of 81.3%.COF-RVG, T@COF-RVG were synthesized similarly, with the yields of 79.3%, 81.5%, and 77.6%, respectively.

**Calculation details:** All calculations were carried out with the Gaussian 09 software. The functional PBE0 and basis set 6-31G(d, p) were used for geometry optimization and frequency calculation. The dispersion was considered at the GD3BJ level. The harmonic frequency calculations were carried out at the same level of theory to verify that all structures have no imaginary frequency. The spin-orbit coupling matrix elements were calculated by TD-DFT calculation as implemented in ORCA program. The natural transition orbitals (NTOs) were visualized by Vesta program with assisted by Multiwfn program.

**Fluorescence sensing experiments of COFs:** The finely ground COF_TFP-DABP_ (1mg) was dissolved in 1 mL of ethanol solution and sonicated to dissolve completely. The solution was diluted 5 times as the original probe concentration for fluorescence detection and PBS was used as a dispersant. The preparation method of 0.2 M PBS with different pH: Weigh 0.716 g Na_2_HPO_4_-12H_2_O respectively, dissolve in 10 mL water, weigh 0.312 g NaH_2_PO_4_-2H_2_O, dissolve in 10 mL water, then just mix 0.2 M Na_2_HPO_4_-12H_2_O and NaH_2_PO_4_-2H_2_O in corresponding proportions to obtain PBS solutions of different pH.

**ROS generation ability of COFs (COF_TFP-DABP_, COF_TFP-PDA_, COF_TFP-DATP_, COF_TFP-AB_, COF_TFB-DABP_):** The fluorescence emission spectra of DCFH and DHR 123 was used to investigate the generation of total ROS and O_2_^.-^. Different samples were incubated with DCFH and DHR 123 (0.2 mM), then the mixed solution was exposed to of LED light (26 mW cm^2^, 400-700 nm). The fluorescence emission spectra change of DCFH and DHR 123 at the wavelength of 525 nm and 535 nm, respectively, every 10s was recorded. The UV-vis spectral change of TMB was used to investigate the generation of .OH. Different samples were incubated with TMB (0.2 mM) and the change of UV-vis absorption spectra at 655 nm was recorded. Concentrations of COFs were 20 μg/mL. The same approach was used by ABDA to assess the ability of COFs to produce oxygen in a single linear state, using UV spectroscopy to detect changes in the intensity of the absorption peak at 378 nm.

**Stability of OT@COF-RVG under** **light irradiation:** The change in fluorescence emission spectra was utilized to study the stability of different samples under light irradiation. The OT@COF-RVG solution was subjected to light irradiation. The changes in fluorescence emission spectra were recorded at different pH (pH=5.5, 6.2,7.2,7.4) for different times (Time=0,2,4,6,12,24h).

**Load efficiency calculation:** To assess the loading content of TMZ and OSMI-4, we measured the fluorescence emission spectra before and after immobilization with TMZ and OSMI-4. Under the excitation of 300 nm, the fluorescence emission peaks of TMZ and OSMI-4 appeared at 395 nm and 370 nm, respectively. Hence, the concentration of unbound TMZ and OSMI-4 in the supernatant solution was determined by fluorescence emission studies at 395 nm and 370 nm. To assess the loading content of TMZ and OSMI-4, we detected the concentration of TMZ and OSMI-4 in the supernatant solution by HPLC assay. The column was an Agilent ZORBAX SB-C18 column (4.6×250 mm 5 μm), the mobile phase was methanol/0.1% acetic acid=20/80 (v/v), the flow rate was 1.0 mL/min, the injection volume was 10 μL, the column temperature was kept at room temperature. The detection wavelength of TMZ was 329 nm, the retention time was 5.5 min. The detection wavelength of OSMI-4 was 202 nm, the retention time was 3.64 min.

The loading capacity (LC) of drugs in nanoparticles were calculated as indicated below (n=3).

**Drug release assay:** The drug-release was study by a dialysis method. A dialysis bag containing 1 ml of 2 mg/ml OT@COF-RVG was immersed in a beaker 10 mL of PBS (0.01 M, pH=5.5/6.2/7.2/7.4), and placed the beaker at 37 ℃ thermostatic water bath. OSMI-4 content of dialysate was detected by fluorescence emission spectra at different time (0 h, 2 h, 4 h, 6 h, 12 h, 24 h). 1 mL of sample dialysate was taken at each time point, and then replaced with 1 ml PBS buffer into the dialysis bag. The cumulative release amount of OSMI-4 from OT@COF-RVG was plotted against time.

**Cell lines:** Various cell lines, including human GBM cell lines (U87MG, U251), mouse glioma cell line (GL261), mouse brain microvascular endothelial cell line (bEnd.3), and human brain microvascular endothelial cell line (hCMEC/D3), were obtained from the Chinese Academy of Sciences Shanghai Cell Bank. The cells were cultured in an incubator at 37 °C, 95% humidity, and 5% CO_2_, and supplemented with 10% fetal bovine serum (FBS) and 1% penicillin/streptomycin in the culture medium.

**Cell culture:** Various cell lines, including human GBM cell lines (U87MG, U251), mouse glioma cell line (GL261), mouse brain microvascular endothelial cell line (bEnd.3), and human brain microvascular endothelial cell line (hCMEC/D3), were obtained from the Chinese Academy of Sciences Shanghai Cell Bank. The cells were cultured in an incubator at 37 °C, 95% humidity, and 5% CO_2_, and supplemented with 10% fetal bovine serum (FBS) and 1% penicillin/streptomycin in the culture medium.

**In vitro ROS generation test:** The intracellular ROS was detected by DCFH-DA and DHE probe. GBM cells were cultured in a six-well plate and subjected to various treatments for 24 hours. After that the plate was incubated with various formulations (COF: 100 μM) for 6 h. Then DCFH-DA or DHE probe was added into the plate and cultured for 30 min. Then the plate was washed by PBS and exposed to light irradiation (26 mW cm^2^, 400-700 nm) at 37 ℃ for 20 min. Finally, the intracellular ROS was evaluated .by confocal laser scanning microscopy (CLSM).

**Cytotoxicity experiment:** Cells were inoculated at a density of 1 × 10^4^ cells per well in a 96-well plate. Subsequently, the cells were exposed to various concentrations and types of nanoparticles for a duration of 4 hours. Proliferation was assessed using Cell Counting Kit-8 (CCK-8, Beyotime) according to the manufacturer's instructions.

**Protein isolation and western blotting analysis:** The experimental procedures outlined in our previous publication were followed^5^, with comprehensive information regarding antibodies provided in Supplementary Table 5.

**ICD analysis:** In the in vitro setting, various nanoparticles and GBM cells were introduced into a six-well plate positioned in a cell climbing apparatus, co-incubated for a duration of 4 hours, and subsequently subjected to white light exposure lasting 3 minutes. The assessment of HMGB1 expression on the GBM cell membrane was conducted through immunoblotting. The detection of CRT on the cell membrane was quantified using immunofluorescence, with GBM cells being co-cultured with diverse nanoparticles and subjected to white light irradiation prior to fixation with 4% paraformaldehyde. Subsequently, the cells were treated with anti-CRT antibodies followed by a secondary antibody at ambient temperature in a sequential manner. Subsequently, the localization of CRT on the cellular membrane was examined through confocal laser scanning microscopy (CLSM). Following cell processing, the supernatant was harvested and extracellular ATP secretion was quantified using the ATP assay kit (Beyotime) in accordance with the provided guidelines.

**In vivo studies:** Male C57BL/6 mice were utilized to establish a mouse intracranial glioma model by injecting GL261 cells carrying luciferase (1×10^5^) into the lateral ventricle. The six-week-old C57BL/6 mice were procured from the Chinese Academy of Sciences Shanghai Laboratory Animal Center. Bioluminescence imaging was employed to monitor tumor growth, followed by euthanasia of the mice for brain tissue sectioning and subsequent haematoxylin–eosin (H&E) and immunofluorescence analysis. The mice were anesthetized using isoflurane and the hair around calvarium was shaved off. Then, the skin was carefully cut with surgical scissors. White light was used to trigger nanoplatform.

**Hemolysis analysis:** Whole blood was collected from C57BL/6 mice and centrifuged at 1500rcf for 3 minutes to isolate red blood cells. The red blood cells were then divided into four 1.5 ml EP tubes and treated with varying concentrations of nanoparticles and Triton X-100. The samples were co-incubated in a 37 °C constant temperature water bath for 3 hours. Subsequently, the samples were centrifuged at 1500rcf for 5 minutes and images were captured. The supernatant from the centrifuge tube was collected, and the absorbance of each group at 570 nm was measured using a UV spectrophotometer to calculate the hemolysis rate.

**ELISA analysis:** Standards or samples were added to each well, incubated at 37°C for 90 minutes and washed 5 times. Biotinylated detection antibody was then added, incubated at 37°C for 60 minutes and the wells washed 5 times. Streptavidin conjugated HRP was added, incubated for 30 min at 37°C and the wells were washed 5 times. Then 3,3′,5,5′-tetramethylbenzidine (TMB) was added and incubated at 37°C for 15 minutes. Stop solution is added at the end and monitored immediately at 450 nm.

**Flow cytometry:** Tumours isolated from mice were cut into small sections. The tumour sections were homogenised in staining buffer with digestive enzymes (collagenase IV, hyaluronidase and deoxyribonuclease I) to obtain a single cell suspension. The cells were then stained with fluorescence-labelled antibodies, F4/80 (Biolegend, cat. no. 123109, Clone: BM8, 1:200 dilution), CD11b (Biolegend, cat. no. 101261, Clone: M1/70, 1:200 dilution), CD206 (Biolegend, cat. no. 141717, Clone: C068C2, 1:200 dilution), CD86 (Biolegend, cat. no. 159215, Clone: A17199A, 1:200 dilution), Foxp3 (Biolegend, cat. no. 126419, Clone: MF-14, 1:200 dilution), CD4 (Biolegend, cat. no. 100411, Clone: GK1.5, 1:200 dilution), CD3 (Biolegend, cat. no. 100205, Clone: 17A2, 1:200 dilution), CD8 (Biolegend, cat. no. 100711, Clone: 53-6.7, 1:200 dilution) following instructions provided by the manufacturer. Eventually, flow cytometer (SONY ID7000) was applied to monitor the stained cells and FlowJo software was employed to analyze the data. Gating set up was based on FMO control.

Lymph nodes were harvested using surgical equipment and then crushed between the surface of frosted microscope slides into a well containing PBS. The cell mixture was then filtered through a 40 μm cell strainer into the conical tube. The cells were then stained with fluorescence-labelled antibodies, CD80 (Biolegend, cat. no. 104705, Clone: 16-10A1, 1:200 dilution), CD86 (Biolegend, cat. no. 159215, Clone: A17199A, 1:200 dilution), CD11c (Biolegend, cat. no. 117307, Clone: N418, 1:200 dilution) per manufacturer’s procedure. Stained cells were analyzed using flow cytometer (SONY ID7000) and evaluated using FlowJo software. Gating set up was based on FMO control.

The spleen was surgically removed using sterilised surgical equipment. The spleen mixture was filtered into a 50 mL conical tube and centrifuged at 500 × g for 5 min. After washing the mixture, the cell pellet was resuspended with erythrocyte lysis solution for 5 min. The cells were then stained with fluorescence-labelled antibodies, CD3 (Biolegend, cat. no. 100205, Clone: 17A2, 1:200 dilution), CD4 (Biolegend, cat. no. 100405, Clone: GK1.5, 1:200 dilution), CD8 (Biolegend, cat. no. 100711, Clone: 53-6.7, 1:200 dilution), CD62L (Biolegend, cat. no. 104449, Clone: MEL-14, 1:200 dilution), and CD44 (Biolegend, cat. no. 103040, Clone: IM7, 1:200 dilution) referring to manufacturer’s protocol. Stained cells were monitored using flow cytometer (SONY ID7000) and evaluated using FlowJo software. Gating set up was based on FMO control.

**Statistical analysis:** Statistical analyses were conducted using GraphPad Prism 9 software. Student's t test was utilized to compare two groups, while one-way analysis of variance (ANOVA) was employed to assess mean differences among multiple groups. Kaplan-Meier analysis, was utilized to evaluate overall survival in C57 mice. Statistical significance was defined as P < 0.05 (*), and all data were reported as mean ± standard deviation (SD) in the results.

**Reporting summary:** Further information on research design is available in the Nature Portfolio Reporting Summary linked to this article.

**Data availability statement:** The data that supports the findings of this study are available from the corresponding author upon reasonable request.

**Table S1.** Atomic coordinates of COF_TFP-DABP_ for the AA-stackinig model (space group *P6*, *a=b=*30.2 Å*, c=*3.4 Å; *α*=90°, *β*=90° and *γ*=120°).

| **Element** | **Atom Number** | **Fractional coordinates of atoms** | | |
| --- | --- | --- | --- | --- |
| H | 1 | 8.558114 | -2.65746 | 0 |
| H | 2 | 8.454645 | -2.58989 | 0 |
| H | 3 | 8.502976 | -2.62924 | 0 |
| H | 4 | 8.637032 | -2.48704 | 0 |
| H | 5 | 8.589351 | -2.4465 | 0 |
| H | 6 | 8.6426 | -2.55675 | 0 |
| H | 7 | 2.657457 | 11.21557 | 0 |
| H | 8 | 2.589885 | 11.04453 | 0 |
| H | 9 | 2.629239 | 11.13222 | 0 |
| H | 10 | 2.487037 | 11.12407 | 0 |
| H | 11 | 2.446497 | 11.03585 | 0 |
| H | 12 | 2.556751 | 11.19935 | 0 |
| H | 13 | -11.2156 | -8.55811 | 0 |
| H | 14 | -11.0445 | -8.45465 | 0 |
| H | 15 | -11.1322 | -8.50298 | 0 |
| H | 16 | -11.1241 | -8.63703 | 0 |
| H | 17 | -11.0358 | -8.58935 | 0 |
| H | 18 | -11.1994 | -8.6426 | 0 |
| H | 19 | -8.55811 | 2.657457 | 0 |
| H | 20 | -8.45465 | 2.589885 | 0 |
| H | 21 | -8.50298 | 2.629239 | 0 |
| H | 22 | -8.63703 | 2.487037 | 0 |
| H | 23 | -8.58935 | 2.446497 | 0 |
| H | 24 | -8.6426 | 2.556751 | 0 |
| H | 25 | -2.65746 | -11.2156 | 0 |
| H | 26 | -2.58989 | -11.0445 | 0 |
| H | 27 | -2.62924 | -11.1322 | 0 |
| H | 28 | -2.48704 | -11.1241 | 0 |
| H | 29 | -2.4465 | -11.0358 | 0 |
| H | 30 | -2.55675 | -11.1994 | 0 |
| H | 31 | 11.21557 | 8.558114 | 0 |
| H | 32 | 11.04453 | 8.454645 | 0 |
| H | 33 | 11.13222 | 8.502976 | 0 |
| H | 34 | 11.12407 | 8.637032 | 0 |
| H | 35 | 11.03585 | 8.589351 | 0 |
| H | 36 | 11.19935 | 8.6426 | 0 |
| C | 1 | 8.631738 | -2.64765 | 0 |
| C | 2 | 8.614113 | -2.70071 | 0 |
| C | 3 | 8.595671 | -2.63071 | 0 |
| C | 4 | 8.492837 | -2.56618 | 0 |
| C | 5 | 8.521793 | -2.59053 | 0 |
| C | 6 | 8.575273 | -2.56223 | 0 |
| C | 7 | 8.598285 | -2.50905 | 0 |
| C | 8 | 8.569024 | -2.48504 | 0 |
| C | 9 | 8.514923 | -2.51235 | 0 |
| C | 10 | 2.647652 | 11.27939 | 0 |
| C | 11 | 2.700707 | 11.31482 | 0 |
| C | 12 | 2.630706 | 11.22638 | 0 |
| C | 13 | 2.566175 | 11.05901 | 0 |
| C | 14 | 2.590534 | 11.11233 | 0 |
| C | 15 | 2.562226 | 11.1375 | 0 |
| C | 16 | 2.509046 | 11.10733 | 0 |
| C | 17 | 2.485036 | 11.05406 | 0 |
| C | 18 | 2.512354 | 11.02728 | 0 |
| C | 19 | -11.2794 | -8.63174 | 0 |
| C | 20 | -11.3148 | -8.61411 | 0 |
| C | 21 | -11.2264 | -8.59567 | 0 |
| C | 22 | -11.059 | -8.49284 | 0 |
| C | 23 | -11.1123 | -8.52179 | 0 |
| C | 24 | -11.1375 | -8.57527 | 0 |
| C | 25 | -11.1073 | -8.59829 | 0 |
| C | 26 | -11.0541 | -8.56902 | 0 |
| C | 27 | -11.0273 | -8.51492 | 0 |
| C | 28 | -8.63174 | 2.647652 | 0 |
| C | 29 | -8.61411 | 2.700707 | 0 |
| C | 30 | -8.59567 | 2.630706 | 0 |
| C | 31 | -8.49284 | 2.566175 | 0 |
| C | 32 | -8.52179 | 2.590534 | 0 |
| C | 33 | -8.57527 | 2.562226 | 0 |
| C | 34 | -8.59829 | 2.509046 | 0 |
| C | 35 | -8.56902 | 2.485036 | 0 |
| C | 36 | -8.51492 | 2.512354 | 0 |
| C | 37 | -2.64765 | -11.2794 | 0 |
| C | 38 | -2.70071 | -11.3148 | 0 |
| C | 39 | -2.63071 | -11.2264 | 0 |
| C | 40 | -2.56618 | -11.059 | 0 |
| C | 41 | -2.59053 | -11.1123 | 0 |
| C | 42 | -2.56223 | -11.1375 | 0 |
| C | 43 | -2.50905 | -11.1073 | 0 |
| C | 44 | -2.48504 | -11.0541 | 0 |
| C | 45 | -2.51235 | -11.0273 | 0 |
| C | 46 | 11.27939 | 8.631738 | 0 |
| C | 47 | 11.31482 | 8.614113 | 0 |
| C | 48 | 11.22638 | 8.595671 | 0 |
| C | 49 | 11.05901 | 8.492837 | 0 |
| C | 50 | 11.11233 | 8.521793 | 0 |
| C | 51 | 11.1375 | 8.575273 | 0 |
| C | 52 | 11.10733 | 8.598285 | 0 |
| C | 53 | 11.05406 | 8.569024 | 0 |
| C | 54 | 11.02728 | 8.514923 | 0 |
| N | 1 | 8.607019 | -2.58156 | 0 |
| N | 2 | 2.581557 | 11.18858 | 0 |
| N | 3 | -11.1886 | -8.60702 | 0 |
| N | 4 | -8.60702 | 2.581557 | 0 |
| N | 5 | -2.58156 | -11.1886 | 0 |
| N | 6 | 11.18858 | 8.607019 | 0 |
| O | 1 | 8.702274 | -2.56707 | 0 |
| O | 2 | 2.567067 | 11.26934 | 0 |
| O | 3 | -11.2693 | -8.70227 | 0 |
| O | 4 | -8.70227 | 2.567067 | 0 |
| O | 5 | -2.56707 | -11.2693 | 0 |
| O | 6 | 11.26934 | 8.702274 | 0 |

**Table S2. Atomic coordinates of COF_TFP-PDA_ for the AA-stackinig model (space group *P6*, *a=c=*22.8 Å*, b=*3.5 Å; *α*=90°, *β*=90° and *γ*=120°).**

| **Element** | **Atom Number** | **Fractional coordinates of atoms** | | |
| --- | --- | --- | --- | --- |
| H | 1 | -8.71756 | 0 | -14.33 |
| H | 2 | -9.01956 | 0 | -14.4945 |
| H | 3 | -8.85498 | 0 | -14.6319 |
| H | 4 | -9.62784 | 0 | -14.53 |
| H | 5 | -9.57508 | 0 | -14.597 |
| H | 6 | -9.76689 | 0 | -14.7743 |
| H | 7 | -9.81964 | 0 | -14.7073 |
| H | 8 | -9.71981 | 0 | -14.521 |
| H | 9 | -9.53975 | 0 | -14.6723 |
| H | 10 | -9.67717 | 0 | -14.9743 |
| H | 11 | -9.67492 | 0 | -14.7833 |
| H | 12 | -9.37517 | 0 | -14.8098 |
| H | 13 | -9.24999 | 0 | -14.5826 |
| H | 14 | -9.13022 | 0 | -14.5299 |
| H | 15 | -9.14474 | 0 | -14.7217 |
| H | 16 | -9.26451 | 0 | -14.7744 |
| H | 17 | -9.04379 | 0 | -14.6297 |
| H | 18 | -8.57521 | 0 | -14.0995 |
| H | 19 | -8.64223 | 0 | -14.2193 |
| H | 20 | -8.81952 | 0 | -14.2048 |
| H | 21 | -8.7525 | 0 | -14.085 |
| H | 22 | -9.35094 | 0 | -14.6746 |
| H | 23 | -8.56624 | 0 | -13.9986 |
| H | 24 | -8.82849 | 0 | -14.3057 |
| C | 1 | -8.81644 | 0 | -14.4138 |
| C | 2 | -8.79421 | 0 | -14.462 |
| C | 3 | -8.83995 | 0 | -14.5331 |
| C | 4 | -8.91041 | 0 | -14.5553 |
| C | 5 | -8.9357 | 0 | -14.5096 |
| C | 6 | -8.88747 | 0 | -14.4391 |
| C | 7 | -8.76738 | 0 | -14.3434 |
| C | 8 | -9.00607 | 0 | -14.5309 |
| C | 9 | -8.81864 | 0 | -14.5821 |
| C | 10 | -9.72889 | 0 | -14.6115 |
| C | 11 | -9.65782 | 0 | -14.5815 |
| C | 12 | -9.62662 | 0 | -14.6213 |
| C | 13 | -9.66583 | 0 | -14.6928 |
| C | 14 | -9.73691 | 0 | -14.7227 |
| C | 15 | -9.7681 | 0 | -14.683 |
| C | 16 | -9.55477 | 0 | -14.7712 |
| C | 17 | -9.48432 | 0 | -14.749 |
| C | 18 | -9.45903 | 0 | -14.7947 |
| C | 19 | -9.50726 | 0 | -14.8652 |
| C | 20 | -9.57829 | 0 | -14.8905 |
| C | 21 | -9.60052 | 0 | -14.8423 |
| C | 22 | -9.57609 | 0 | -14.7222 |
| C | 23 | -9.62735 | 0 | -14.9609 |
| C | 24 | -9.38866 | 0 | -14.7734 |
| C | 25 | -9.26952 | 0 | -14.6837 |
| C | 26 | -9.22842 | 0 | -14.6126 |
| C | 27 | -9.15747 | 0 | -14.5814 |
| C | 28 | -9.12521 | 0 | -14.6206 |
| C | 29 | -9.16631 | 0 | -14.6917 |
| C | 30 | -9.23726 | 0 | -14.7229 |
| C | 31 | -8.65674 | 0 | -14.08 |
| C | 32 | -8.62677 | 0 | -14.1211 |
| C | 33 | -8.66652 | 0 | -14.192 |
| C | 34 | -8.73799 | 0 | -14.2243 |
| C | 35 | -8.76796 | 0 | -14.1832 |
| C | 36 | -8.72821 | 0 | -14.1122 |
| N | 1 | -9.75351 | 0 | -14.5682 |
| N | 2 | -9.64122 | 0 | -14.736 |
| N | 3 | -9.33742 | 0 | -14.7083 |
| N | 4 | -9.05731 | 0 | -14.596 |
| N | 5 | -8.61346 | 0 | -14.0121 |
| N | 6 | -8.78127 | 0 | -14.2922 |
| O | 1 | -8.90916 | 0 | -14.3983 |
| O | 2 | -8.7317 | 0 | -14.4403 |
| O | 3 | -8.95123 | 0 | -14.6178 |
| O | 4 | -9.66303 | 0 | -14.8639 |
| O | 5 | -9.44349 | 0 | -14.6865 |
| O | 6 | -9.48557 | 0 | -14.906 |

**Table S3. Atomic coordinates of COF_TFP-DATP_ for the AA-stackinig model (space group *P6*, *a=c=*38.0 Å*, b=*3.5 Å; *α*=90°, *β*=90° and *γ*=120°).**

| **Element** | **Atom Number** | **Fractional coordinates of atoms** | | |
| --- | --- | --- | --- | --- |
| H | 1 | 1.42594 | 0 | -9.53908 |
| H | 2 | 1.322732 | 0 | -9.46138 |
| H | 3 | 1.503646 | 0 | -9.35817 |
| H | 4 | 1.543199 | 0 | -9.69212 |
| H | 5 | 1.511336 | 0 | -9.65509 |
| H | 6 | 1.625012 | 0 | -9.54696 |
| H | 7 | 1.656874 | 0 | -9.584 |
| H | 8 | 1.481379 | 0 | -9.62067 |
| H | 9 | 1.449205 | 0 | -9.58264 |
| H | 10 | 1.56234 | 0 | -9.47463 |
| H | 11 | 1.595459 | 0 | -9.51215 |
| H | 12 | 1.686831 | 0 | -9.61841 |
| H | 13 | 1.719006 | 0 | -9.65645 |
| H | 14 | 1.605871 | 0 | -9.76445 |
| H | 15 | 1.572751 | 0 | -9.72694 |
| H | 16 | 1.506162 | 0 | -9.47091 |
| H | 17 | 1.84548 | 0 | -9.77771 |
| H | 18 | 1.664566 | 0 | -9.88092 |
| H | 19 | 1.742271 | 0 | -9.7 |
| H | 20 | 2.11578 | 0 | -9.66045 |
| H | 21 | 2.046882 | 0 | -9.69231 |
| H | 22 | 2.052432 | 0 | -9.57864 |
| H | 23 | 2.12133 | 0 | -9.54677 |
| H | 24 | 1.98251 | 0 | -9.72227 |
| H | 25 | 1.912299 | 0 | -9.75444 |
| H | 26 | 1.917431 | 0 | -9.64131 |
| H | 27 | 1.988064 | 0 | -9.60819 |
| H | 28 | 2.185702 | 0 | -9.51682 |
| H | 29 | 2.255913 | 0 | -9.48464 |
| H | 30 | 2.250781 | 0 | -9.59778 |
| H | 31 | 2.180147 | 0 | -9.6309 |
| H | 32 | 1.857528 | 0 | -9.69749 |
| H | 33 | 1.662049 | 0 | -9.76818 |
| H | 34 | 2.310683 | 0 | -9.5416 |
| H | 35 | 1.511526 | 0 | -9.15122 |
| H | 36 | 1.548562 | 0 | -9.08232 |
| H | 37 | 1.656687 | 0 | -9.08787 |
| H | 38 | 1.619651 | 0 | -9.15677 |
| H | 39 | 1.582976 | 0 | -9.01795 |
| H | 40 | 1.621013 | 0 | -8.94773 |
| H | 41 | 1.729016 | 0 | -8.95287 |
| H | 42 | 1.691502 | 0 | -9.0235 |
| H | 43 | 1.585236 | 0 | -9.22114 |
| H | 44 | 1.5472 | 0 | -9.29135 |
| H | 45 | 1.439197 | 0 | -9.28622 |
| H | 46 | 1.476711 | 0 | -9.21558 |
| H | 47 | 1.732741 | 0 | -8.89296 |
| H | 48 | 1.435472 | 0 | -9.34612 |
| C | 1 | 1.390562 | 0 | -9.495 |
| C | 2 | 1.374205 | 0 | -9.46853 |
| C | 3 | 1.402195 | 0 | -9.426 |
| C | 4 | 1.445017 | 0 | -9.40964 |
| C | 5 | 1.45956 | 0 | -9.43763 |
| C | 6 | 1.433096 | 0 | -9.48045 |
| C | 7 | 1.447099 | 0 | -9.50896 |
| C | 8 | 1.331699 | 0 | -9.48253 |
| C | 9 | 1.47352 | 0 | -9.36713 |
| C | 10 | 1.561914 | 0 | -9.66151 |
| C | 11 | 1.542177 | 0 | -9.63857 |
| C | 12 | 1.563359 | 0 | -9.59543 |
| C | 13 | 1.606296 | 0 | -9.57758 |
| C | 14 | 1.626033 | 0 | -9.60052 |
| C | 15 | 1.604851 | 0 | -9.64366 |
| C | 16 | 1.543172 | 0 | -9.57196 |
| C | 17 | 1.500069 | 0 | -9.59007 |
| C | 18 | 1.480173 | 0 | -9.56729 |
| C | 19 | 1.50243 | 0 | -9.52449 |
| C | 20 | 1.545011 | 0 | -9.50566 |
| C | 21 | 1.564635 | 0 | -9.52868 |
| C | 22 | 1.625038 | 0 | -9.66713 |
| C | 23 | 1.668141 | 0 | -9.64902 |
| C | 24 | 1.688037 | 0 | -9.6718 |
| C | 25 | 1.665781 | 0 | -9.7146 |
| C | 26 | 1.623199 | 0 | -9.73343 |
| C | 27 | 1.603575 | 0 | -9.71041 |
| C | 28 | 1.766017 | 0 | -9.81309 |
| C | 29 | 1.723195 | 0 | -9.82944 |
| C | 30 | 1.708652 | 0 | -9.80145 |
| C | 31 | 1.735116 | 0 | -9.75863 |
| C | 32 | 1.777649 | 0 | -9.74409 |
| C | 33 | 1.794007 | 0 | -9.77055 |
| C | 34 | 1.836513 | 0 | -9.75655 |
| C | 35 | 1.694692 | 0 | -9.87195 |
| C | 36 | 1.721112 | 0 | -9.73013 |
| C | 37 | 2.10388 | 0 | -9.64173 |
| C | 38 | 2.0612 | 0 | -9.66147 |
| C | 39 | 2.039245 | 0 | -9.64029 |
| C | 40 | 2.064332 | 0 | -9.59735 |
| C | 41 | 2.107012 | 0 | -9.57761 |
| C | 42 | 2.128967 | 0 | -9.5988 |
| C | 43 | 1.995586 | 0 | -9.66047 |
| C | 44 | 1.970595 | 0 | -9.70358 |
| C | 45 | 1.927921 | 0 | -9.72347 |
| C | 46 | 1.907375 | 0 | -9.70122 |
| C | 47 | 1.931127 | 0 | -9.65864 |
| C | 48 | 1.97377 | 0 | -9.63901 |
| C | 49 | 2.172626 | 0 | -9.57861 |
| C | 50 | 2.197616 | 0 | -9.53551 |
| C | 51 | 2.24029 | 0 | -9.51561 |
| C | 52 | 2.260836 | 0 | -9.53787 |
| C | 53 | 2.237085 | 0 | -9.58045 |
| C | 54 | 2.194442 | 0 | -9.60007 |
| C | 55 | 1.542142 | 0 | -9.13932 |
| C | 56 | 1.565084 | 0 | -9.09664 |
| C | 57 | 1.608221 | 0 | -9.07468 |
| C | 58 | 1.626071 | 0 | -9.09977 |
| C | 59 | 1.603129 | 0 | -9.14245 |
| C | 60 | 1.559992 | 0 | -9.1644 |
| C | 61 | 1.631693 | 0 | -9.03102 |
| C | 62 | 1.613581 | 0 | -9.00603 |
| C | 63 | 1.636359 | 0 | -8.96336 |
| C | 64 | 1.679161 | 0 | -8.94281 |
| C | 65 | 1.697991 | 0 | -8.96656 |
| C | 66 | 1.674972 | 0 | -9.00921 |
| C | 67 | 1.53652 | 0 | -9.20806 |
| C | 68 | 1.554632 | 0 | -9.23305 |
| C | 69 | 1.531854 | 0 | -9.27573 |
| C | 70 | 1.489052 | 0 | -9.29627 |
| C | 71 | 1.470221 | 0 | -9.27252 |
| C | 72 | 1.493241 | 0 | -9.22988 |
| N | 1 | 1.48655 | 0 | -9.49944 |
| N | 2 | 1.866452 | 0 | -9.7171 |
| N | 3 | 1.681661 | 0 | -9.73964 |
| N | 4 | 2.30176 | 0 | -9.52199 |
| N | 5 | 1.704205 | 0 | -8.90189 |
| N | 6 | 1.464008 | 0 | -9.3372 |
| O | 1 | 1.366922 | 0 | -9.5327 |
| O | 2 | 1.497261 | 0 | -9.42357 |
| O | 3 | 1.388135 | 0 | -9.40236 |
| O | 4 | 1.780078 | 0 | -9.83673 |
| O | 5 | 1.801289 | 0 | -9.70639 |
| O | 6 | 1.670951 | 0 | -9.81551 |

**Table S4.** Atomic coordinates of COF_TFB-DABP_ for the AA-stackinig model (space group *P6*, *a=b=*30 Å*, c=*3.7 Å; *α*=90°, *β*=90° and *γ*=120°).

| **Element** | **Atom Number** | **Fractional coordinates of atoms** | | |
| --- | --- | --- | --- | --- |
| H | 1 | -8.30341 | -7.57589 | 0 |
| H | 2 | -8.21719 | -7.5562 | 0 |
| H | 3 | -8.04416 | -7.4537 | 0 |
| H | 4 | -8.13249 | -7.50176 | 0 |
| H | 5 | -8.12561 | -7.63739 | 0 |
| H | 6 | -8.03678 | -7.59002 | 0 |
| H | 7 | 7.575889 | -0.72752 | 0 |
| H | 8 | 7.556201 | -0.66098 | 0 |
| H | 9 | 7.453695 | -0.59047 | 0 |
| H | 10 | 7.501756 | -0.63074 | 0 |
| H | 11 | 7.637388 | -0.48823 | 0 |
| H | 12 | 7.59002 | -0.44676 | 0 |
| H | 13 | 0.727519 | 8.303408 | 0 |
| H | 14 | 0.660983 | 8.217185 | 0 |
| H | 15 | 0.590469 | 8.044164 | 0 |
| H | 16 | 0.630738 | 8.132494 | 0 |
| H | 17 | 0.488225 | 8.125613 | 0 |
| H | 18 | 0.446757 | 8.036776 | 0 |
| H | 19 | 8.303408 | 7.575889 | 0 |
| H | 20 | 8.217185 | 7.556201 | 0 |
| H | 21 | 8.044164 | 7.453695 | 0 |
| H | 22 | 8.132494 | 7.501756 | 0 |
| H | 23 | 8.125613 | 7.637388 | 0 |
| H | 24 | 8.036776 | 7.59002 | 0 |
| H | 25 | -7.57589 | 0.727519 | 0 |
| H | 26 | -7.5562 | 0.660983 | 0 |
| H | 27 | -7.4537 | 0.590469 | 0 |
| H | 28 | -7.50176 | 0.630738 | 0 |
| H | 29 | -7.63739 | 0.488225 | 0 |
| H | 30 | -7.59002 | 0.446757 | 0 |
| H | 31 | -0.72752 | -8.30341 | 0 |
| H | 32 | -0.66098 | -8.21719 | 0 |
| H | 33 | -0.59047 | -8.04416 | 0 |
| H | 34 | -0.63074 | -8.13249 | 0 |
| H | 35 | -0.48823 | -8.12561 | 0 |
| H | 36 | -0.44676 | -8.03678 | 0 |
| H | 37 | 0.44125 | 0.79879 | 1 |
| H | 38 | -0.79879 | -0.35754 | 1 |
| H | 39 | 0.357541 | -0.44125 | 1 |
| H | 40 | -0.44125 | -0.79879 | 1 |
| H | 41 | 0.79879 | 0.357541 | 1 |
| H | 42 | -0.35754 | 0.44125 | 1 |
| C | 1 | -8.27982 | -7.63064 | 0 |
| C | 2 | -8.31619 | -7.61424 | 0 |
| C | 3 | -8.22724 | -7.59387 | 0 |
| C | 4 | -8.05904 | -7.49218 | 0 |
| C | 5 | -8.11272 | -7.52095 | 0 |
| C | 6 | -8.13846 | -7.57479 | 0 |
| C | 7 | -8.10847 | -7.59836 | 0 |
| C | 8 | -8.05482 | -7.56929 | 0 |
| C | 9 | -8.02747 | -7.51483 | 0 |
| C | 10 | 7.630644 | -0.64917 | 0 |
| C | 11 | 7.614237 | -0.70195 | 0 |
| C | 12 | 7.593874 | -0.63336 | 0 |
| C | 13 | 7.49218 | -0.56686 | 0 |
| C | 14 | 7.52095 | -0.59177 | 0 |
| C | 15 | 7.574792 | -0.56366 | 0 |
| C | 16 | 7.598363 | -0.5101 | 0 |
| C | 17 | 7.569285 | -0.48554 | 0 |
| C | 18 | 7.514827 | -0.51264 | 0 |
| C | 19 | 0.649174 | 8.279818 | 0 |
| C | 20 | 0.701951 | 8.316188 | 0 |
| C | 21 | 0.633361 | 8.227235 | 0 |
| C | 22 | 0.56686 | 8.05904 | 0 |
| C | 23 | 0.591773 | 8.112723 | 0 |
| C | 24 | 0.563662 | 8.138455 | 0 |
| C | 25 | 0.510102 | 8.108465 | 0 |
| C | 26 | 0.485536 | 8.054821 | 0 |
| C | 27 | 0.512639 | 8.027467 | 0 |
| C | 28 | 8.279818 | 7.630644 | 0 |
| C | 29 | 8.316188 | 7.614237 | 0 |
| C | 30 | 8.227235 | 7.593874 | 0 |
| C | 31 | 8.05904 | 7.49218 | 0 |
| C | 32 | 8.112723 | 7.52095 | 0 |
| C | 33 | 8.138455 | 7.574792 | 0 |
| C | 34 | 8.108465 | 7.598363 | 0 |
| C | 35 | 8.054821 | 7.569285 | 0 |
| C | 36 | 8.027467 | 7.514827 | 0 |
| C | 37 | -7.63064 | 0.649174 | 0 |
| C | 38 | -7.61424 | 0.701951 | 0 |
| C | 39 | -7.59387 | 0.633361 | 0 |
| C | 40 | -7.49218 | 0.56686 | 0 |
| C | 41 | -7.52095 | 0.591773 | 0 |
| C | 42 | -7.57479 | 0.563662 | 0 |
| C | 43 | -7.59836 | 0.510102 | 0 |
| C | 44 | -7.56929 | 0.485536 | 0 |
| C | 45 | -7.51483 | 0.512639 | 0 |
| C | 46 | -0.64917 | -8.27982 | 0 |
| C | 47 | -0.70195 | -8.31619 | 0 |
| C | 48 | -0.63336 | -8.22724 | 0 |
| C | 49 | -0.56686 | -8.05904 | 0 |
| C | 50 | -0.59177 | -8.11272 | 0 |
| C | 51 | -0.56366 | -8.13846 | 0 |
| C | 52 | -0.5101 | -8.10847 | 0 |
| C | 53 | -0.48554 | -8.05482 | 0 |
| C | 54 | -0.51264 | -8.02747 | 0 |
| N | 1 | -8.4164 | -7.81008 | 0 |
| N | 2 | 7.810075 | -0.60633 | 0 |
| N | 3 | 0.606326 | 8.416401 | 0 |
| N | 4 | 8.416401 | 7.810075 | 0 |
| N | 5 | -7.81008 | 0.606326 | 0 |
| N | 6 | -0.60633 | -8.4164 | 0 |

**Table S5.** Antibodies information

| **Antibodies** | **Source** | **Category No.** |
| --- | --- | --- |
| GAPDH Monoclonal antibody | Proteintech | 60004-1-Ig |
| Beta Actin Monoclonal antibody | Proteintech | 66009-1-Ig |
| Beta Tubulin Polyclonal antibody | Proteintech | 10068-1-AP |
| O-GlcNAc MultiMab® Rabbit mAb mix | Cell Signaling Technology | 82332S |
| Anti-OGT / O-Linked N-Acetylglucosamine Transferase | Abcam | ab177941 |
| EGFR Monoclonal antibody | Proteintech | 66455-1-Ig |
| HGS Polyclonal antibody | Proteintech | 10390-1-AP |
| Hrs (phospho Tyr334) Polyclonal Antibody | Immunoway | YP0935 |
| Hrs (phospho Tyr216) Polyclonal Antibody | Immunoway | YP0132 |
| PD-L1/CD274 Monoclonal antibody | Proteintech | 66248-1-Ig |
| PD-L1 (E1L3N®) XP® Rabbit mAb | Cell Signaling Technology | 13684T |
| HMGB1 Polyclonal antibody | Proteintech | 10829-1-AP |
| calreticulin Polyclonal antibody | Proteintech | 27298-1-AP |
| ZO-1 Polyclonal antibody | Proteintech | 21773-1-AP |
| AMPKα1/2 Polyclonal Antibody | Immunoway | YT0216 |
| AMPKα1/2 (phospho Thr183/172) Polyclonal  Antibody | Immunoway | YP0575 |
| Claudin-5 Polyclonal Antibody | Immunoway | YT0953 |

**
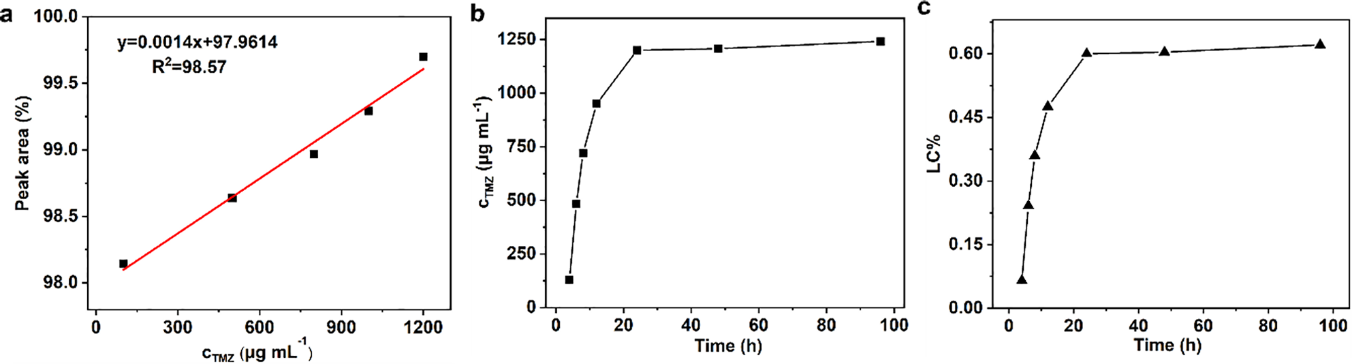
Fig. S1** (a) The standard curve of TMZ peak occupancy versus concentration was plotted by linear regression using peak area occupancy as the vertical coordinate and TMZ concentration as the horizontal coordinate. (b) The concentration of TMZ in OT@COF-RVG was determined using the standard curve in (a). (c) Load efficiency calculation of TMZ in OT@COF-RVG.

**
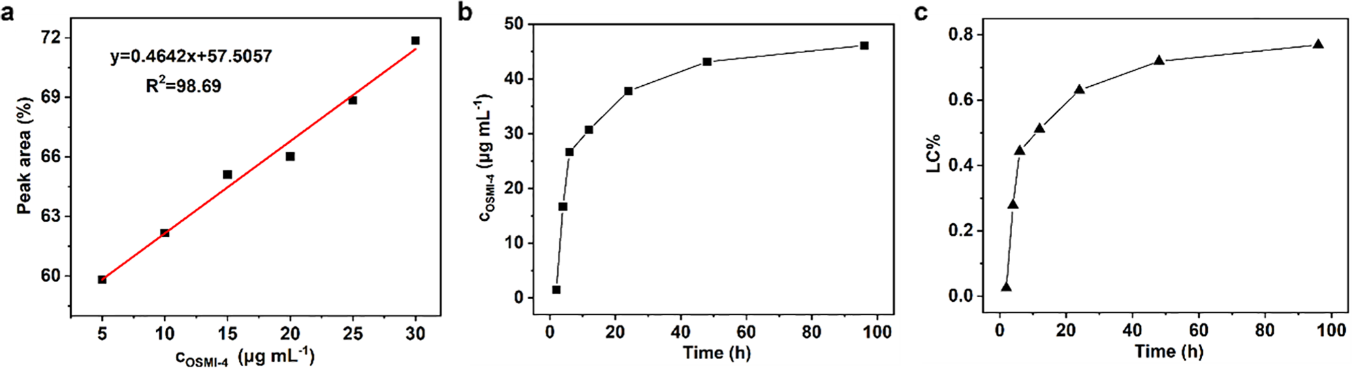
Fig. S2** (a) The standard curve of OSMI-4 peak occupancy versus concentration was plotted by linear regression using peak area occupancy as the vertical coordinate and OSMI-4 concentration as the horizontal coordinate. (b) The concentration of OSMI-4 in OT@COF-RVG was determined using the standard curve in (a). (c) Load efficiency calculation of OSMI-4 in OT@COF-RVG.

**
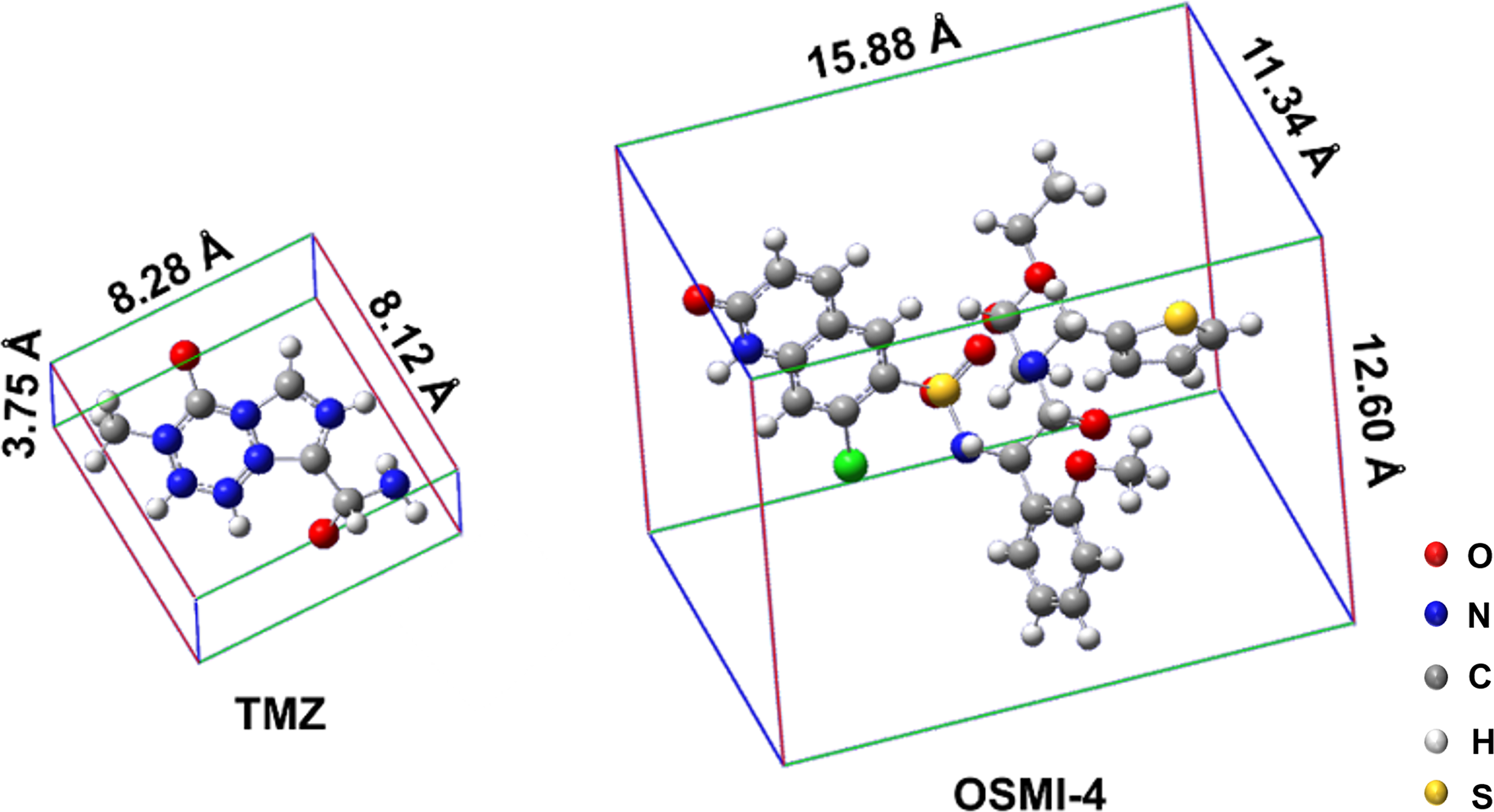
**

**Fig. S3** The three-dimensional dimensions of TMZ and OSMI-4 molecules.


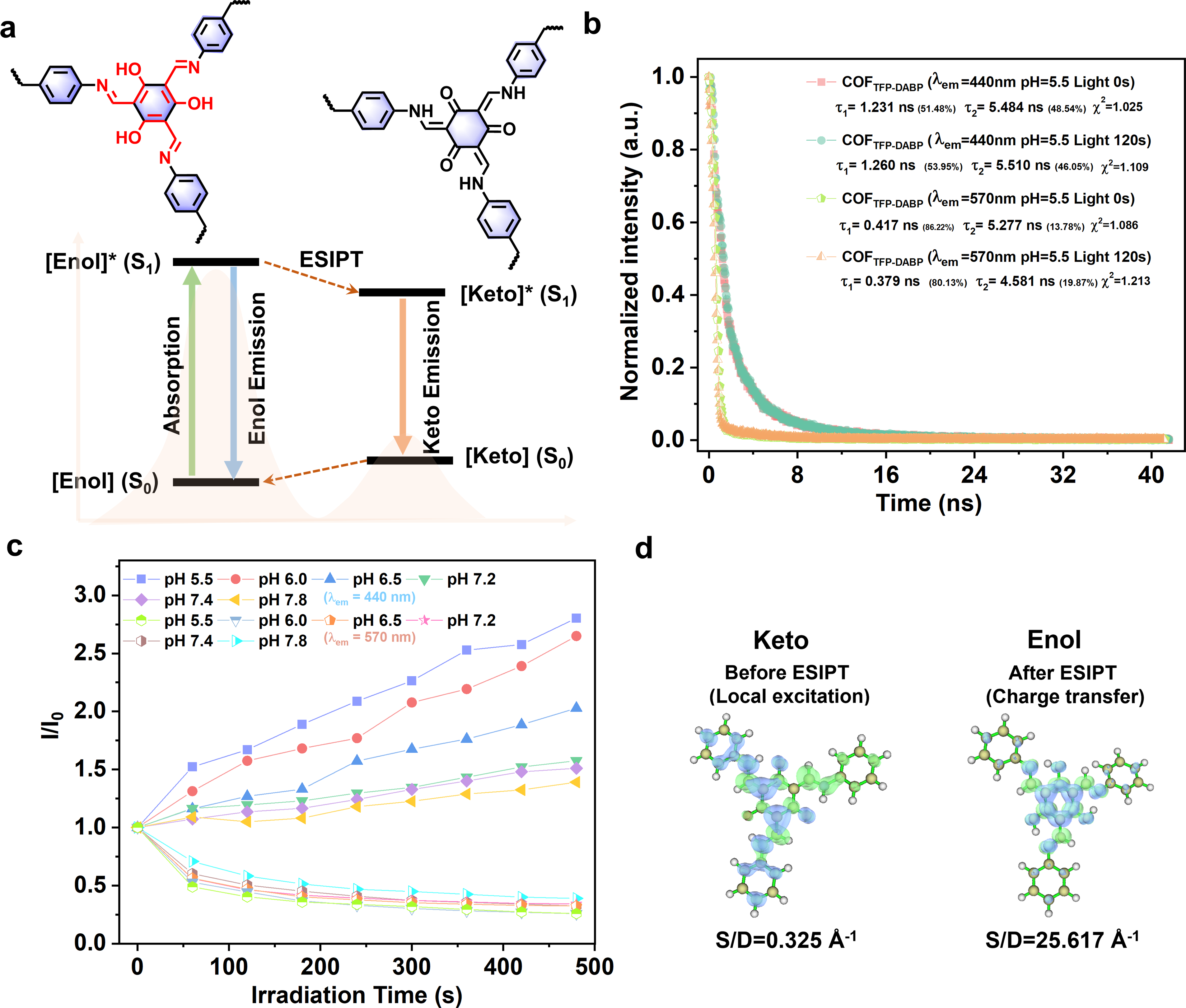


**Fig. S4** (a) Scheme of the ESIPT process from Enol to Keto forms (* indicate the excited state). (b) Normalized fluorescence lifetime differences monitored at 440 nm (Enol form dominates) and 570 nm (Keto form dominates) as a white light irradiation time for COF_TFP-DABP_ in PBS of pH 5.5. (c) The fluorescence intensity of COF_TFP-DABP_ at 440 nm and 570 nm in PBS with different pH values varies with irradiation time. (d) Electron-hole distributions for the hexagonal cutout models with different linkers (isovalue = 0.0015, calculated on the TD-PBE0-D3/def2-SVP level). Blue and green regions represent the holes and electrons, respectively.


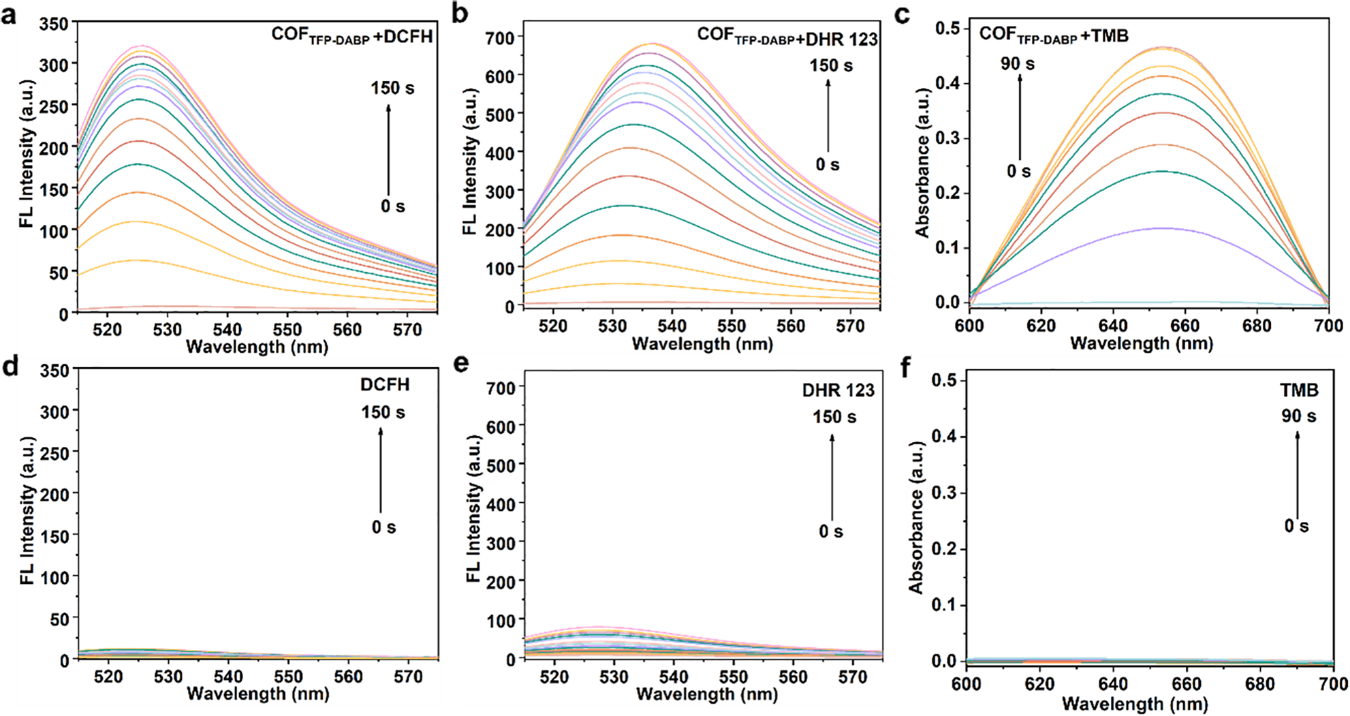


**Fig. S5 Characterization of the photodynamic performance of COF_TFP-DABP_ in PBS.** (a) ROS generation of COF_TFP-DABP_ (10 μg/mL) upon light irradiation using DCFH (100 μM) as an indicator. (b) ROS generation of COF_TFP-DABP_ (10 μg/mL) upon light irradiation using DHR123 (100 μM) as an indicator. (c) Absorbance decay of TMB (100 μM) in the presence of COF_TFP-DABP_ (10 μg/mL) under light irradiation. (d-f) The control group was the same test conditions without COF_TFP-DABP_. The time interval of the test is 10s and the white light irradiation power is 26 mW cm^−2^.


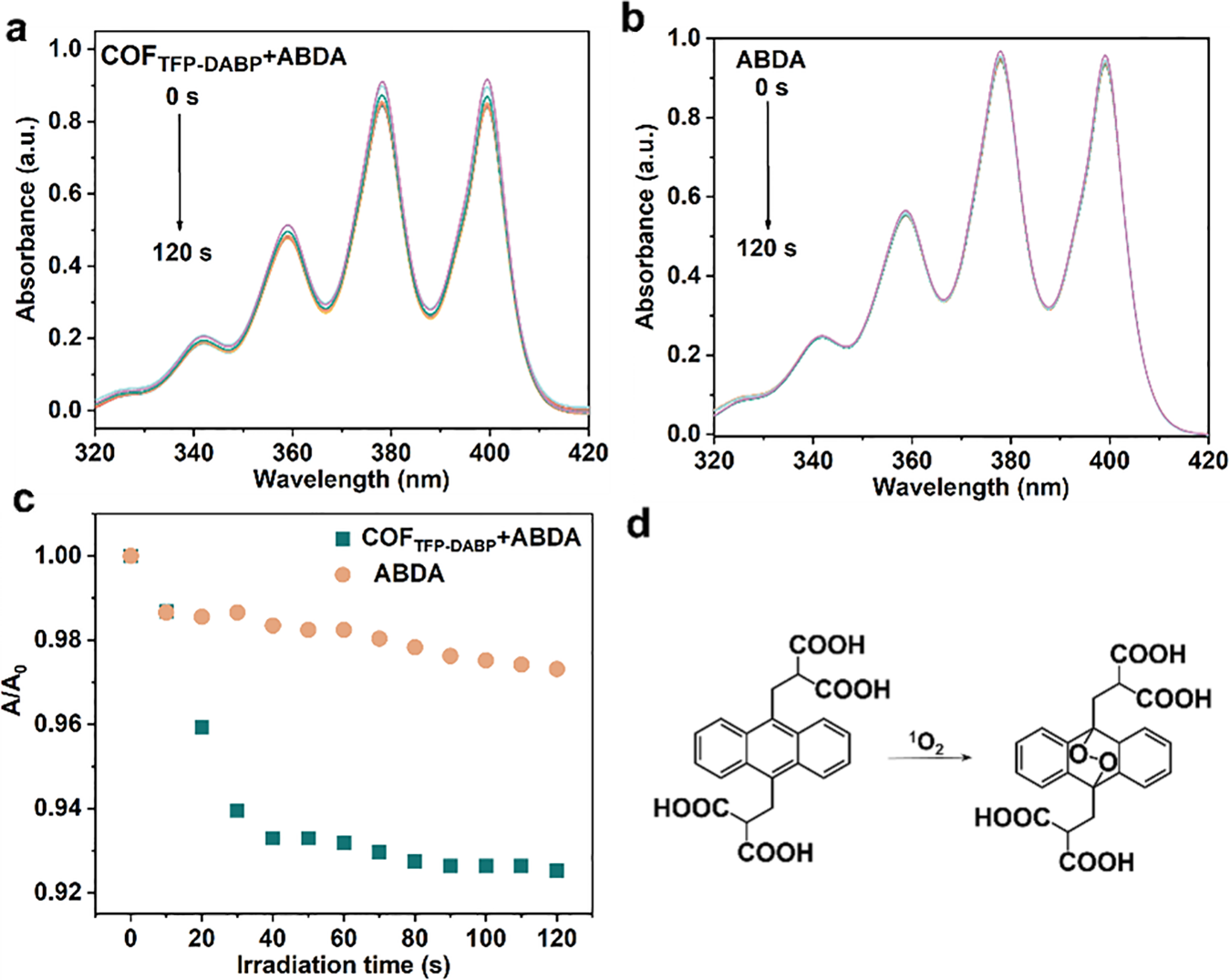


**Fig. S6** **Characterization of the photodynamic performance of COF_TFP-DABP_ in PBS.** Absorption spectra of ABDA (100 μM) in the (a) absence and (b) presence of COF_TFP-DABP_ (10 μg/mL) under light irradiation. (c) The absorbance change of ABDA at 378 nm as a function of irradiation time in the presence of COF_TFP-DABP_. (d) Chemical structure of ABDA and structural changes upon interaction with ^1^O_2_. The time interval of the test is 10s and the white light irradiation power is 26 mW cm^−2^.


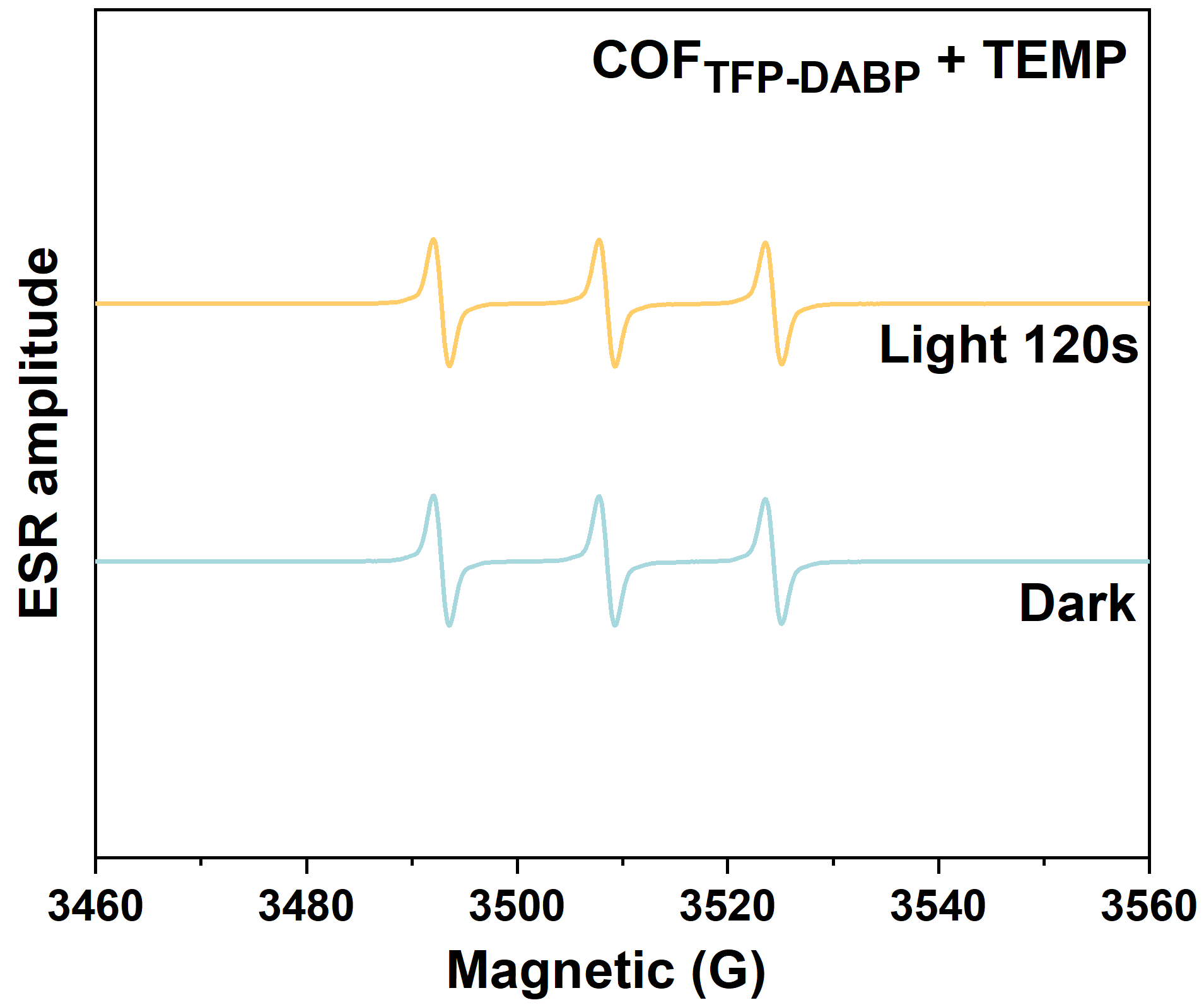


**Fig. S7** ESR spectra to detect ^1^O_2_ from COF_TFP-DABP_ under light irradiation, using triacetonamine hydrochloride (TEMP) as the spin-trap agent, the white light irradiation power is 26 mW cm^−2^.

**
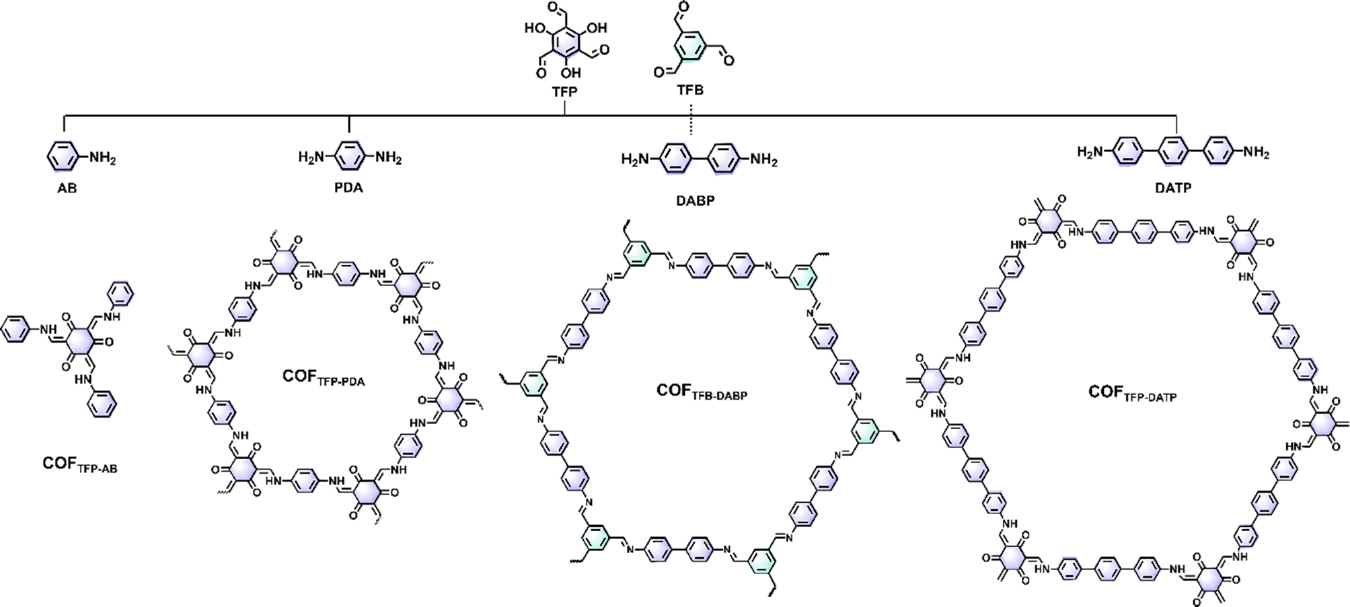
Fig. S8** The chemical structures of the COF_TFP-AB_, COF_TFP-PDA_, COF_TFB-DABP_ and COF_TFB-DATP_.


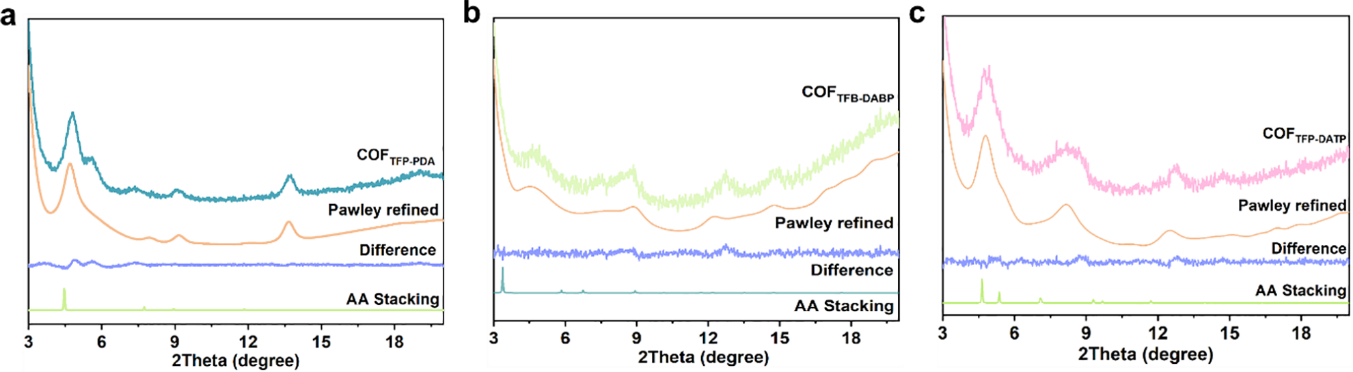


**Fig. S9** PXRD patterns and Pawley refined with a minimum difference, simulated PXRD patterns for AA stacking of (a) COF_TFP-PDA_, (b) COF_TFB-DABP_, and (c) COF_TFP-DATP_.


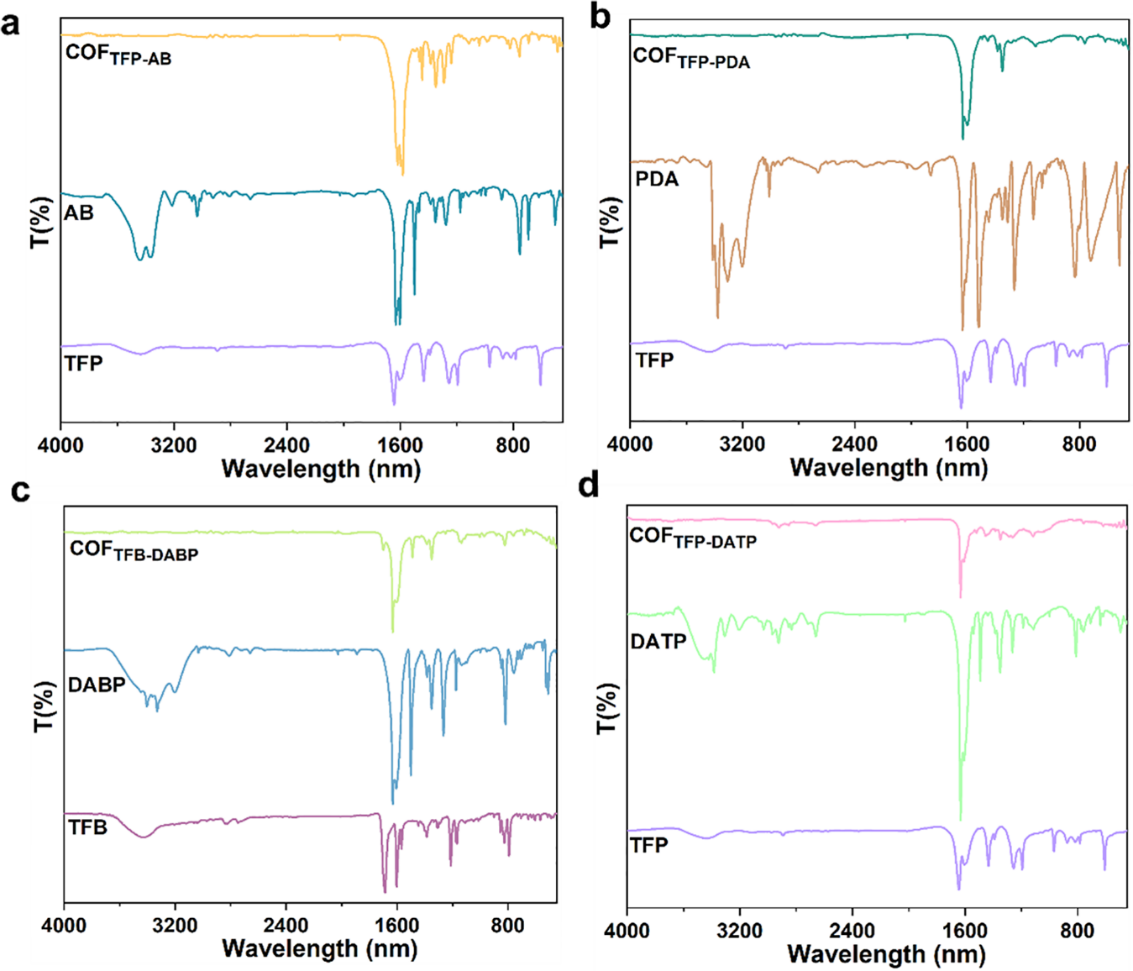


**Fig. S10** FTIR spectra of (a) TFP, AB, COF_TFP-AB_, (b) TFP, PDA, COF_TFP-PDA_, (c) TFB, DABP, COF_TFB-DABP_, and (d)TFP, DATP, COF_TFP-DATP_.

**
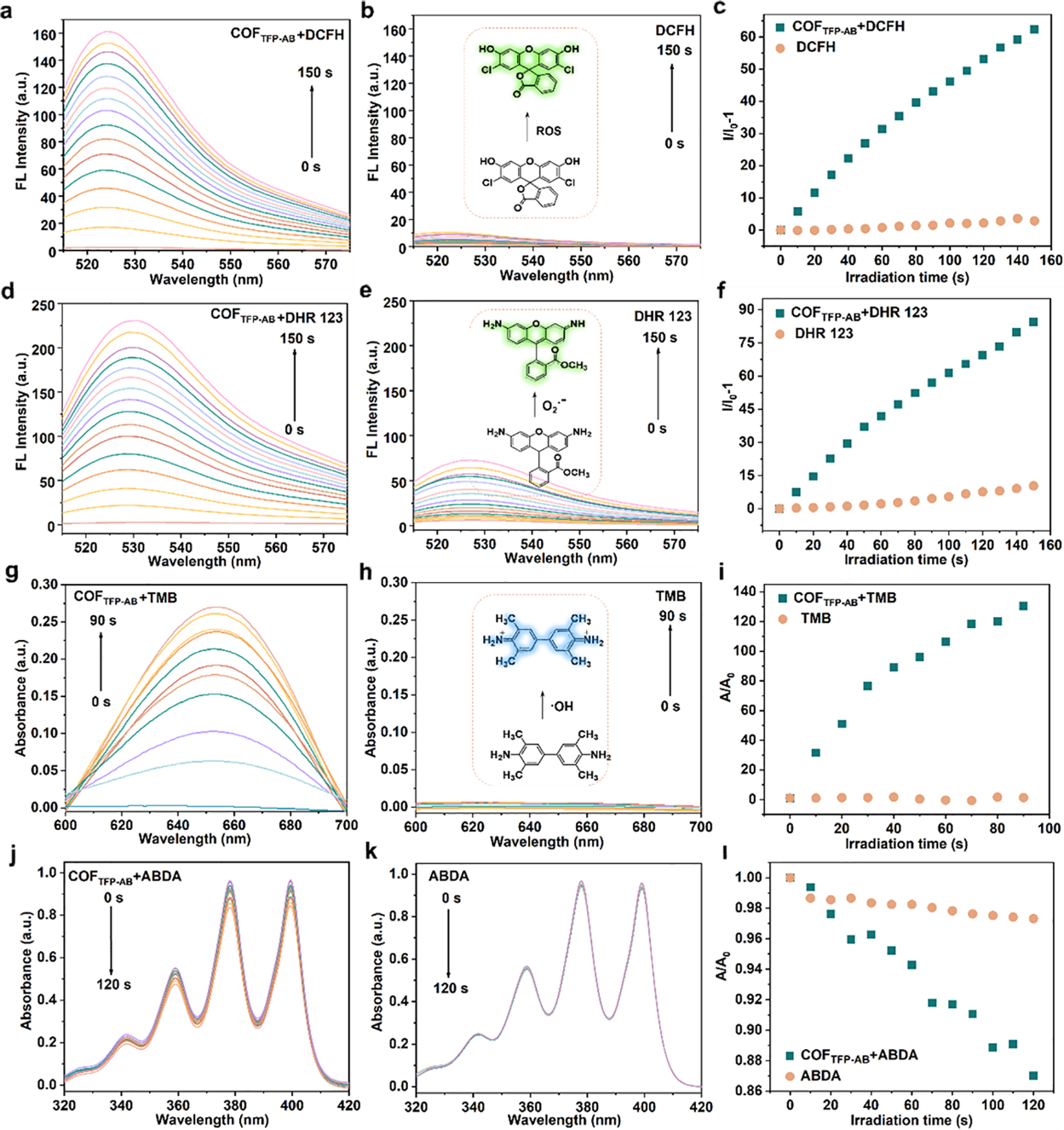
**

**Fig. S11 Characterization of the photodynamic performance of** **COF_TFP-AB_ in PBS.** (a) ROS generation of COF_TFP-AB_ (10 μg/mL) upon light irradiation using DCFH (100 μM) as an indicator. (d) ROS generation of COF_TFP-AB_ (10 μg/mL) upon light irradiation using DHR123 (100 μM) as an indicator. (g) Absorbance decay of TMB (100 μM) in the presence of COF_TFP-AB_ (10 μg/mL) under light irradiation. (b, e, h) The control group was the same test conditions without COF_TFP-AB_. The insets in Fig. S8b, e, g show the structure of the corresponding probes as well as the structural changes when indicated. Absorption spectra of ABDA (100 μM) in the (j) presence and (k) absence of COF_TFP-AB_ (10 μg/mL) under light irradiation. The fluorescence emission spectrum changes of (c) DCFH at 525 nm and (f) DHR 123 at 535 nm and the absorbance changes of (i) TMB at 655 nm and (j) ABDA at 378 nm as a function of irradiation time in the presence of COF_TFP-AB_. The time interval of the test is 10s and the white light irradiation power is 26 mW cm^−2^.

**
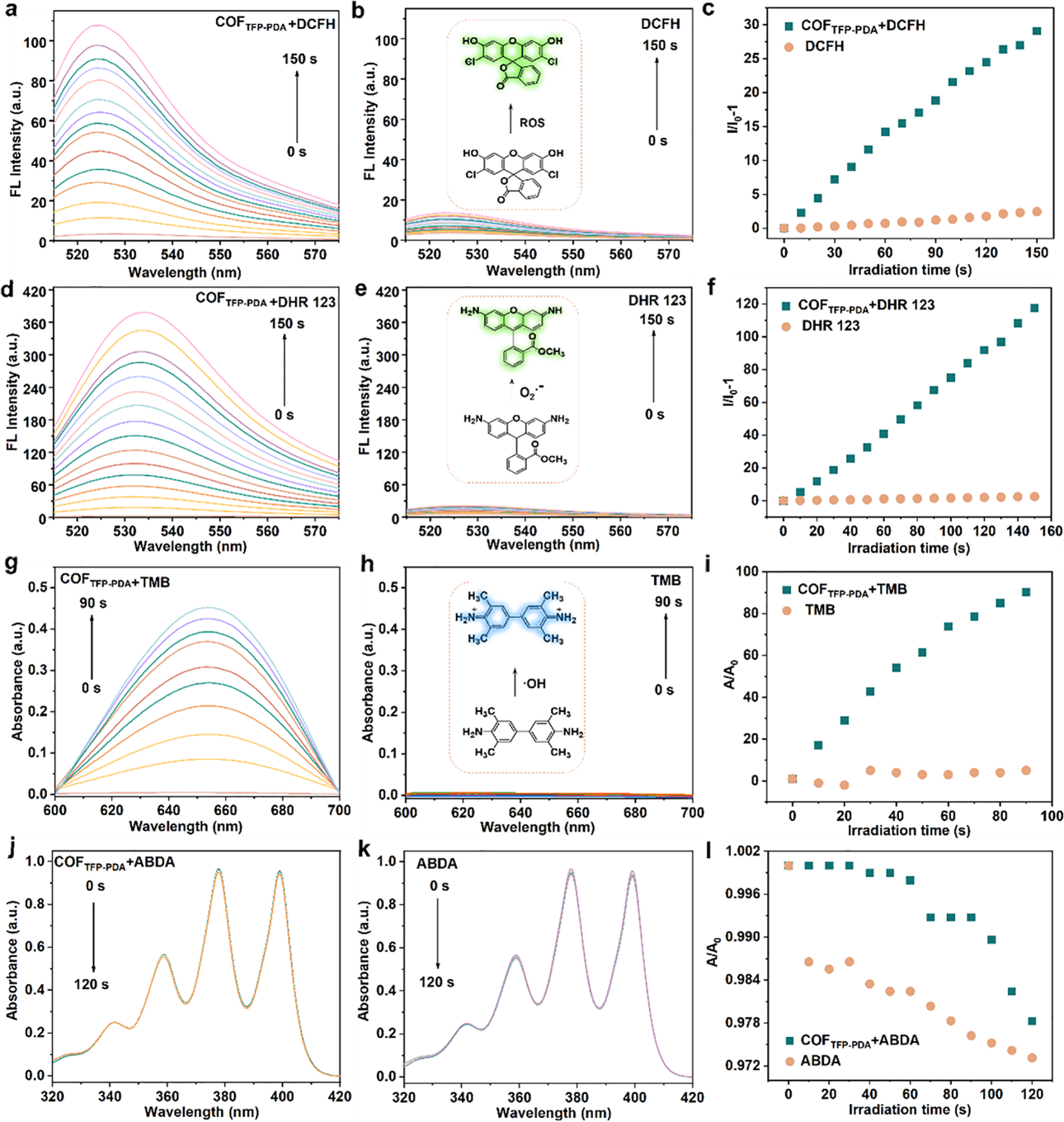
**

**Fig. S12 Characterization of the photodynamic performance of COF_TFP-PDA_ in PBS.** (a) ROS generation of COF_TFP-PDA_ (10 μg/mL) upon light irradiation using DCFH (100 μM) as an indicator. (d) ROS generation of COF_TFP-PDA_ (10 μg/mL) upon light irradiation using DHR123 (100 μM) as an indicator. (g) Absorbance decay of TMB (100 μM) in the presence of COF_TFP-PDA_ (10 μg/mL) under light irradiation. (b, e, h) The control group was the same test conditions without COF_TFP-PDA_. The insets in Fig. S9b, e, g show the structure of the corresponding probes as well as the structural changes when indicated. Absorption spectra of ABDA (100 μM) in the (j) presence and (k) absence of COF_TFP-PDA_ (10 μg/mL) under light irradiation. The fluorescence emission spectrum changes of (c) DCFH at 525 nm and (f) DHR 123 at 535 nm and the absorbance changes of (i) TMB at 655 nm and (j) ABDA at 378 nm as a function of irradiation time in the presence of COF_TFP-PDA_. The time interval of the test is 10s and the white light irradiation power is 26 mW cm^−2^.

**
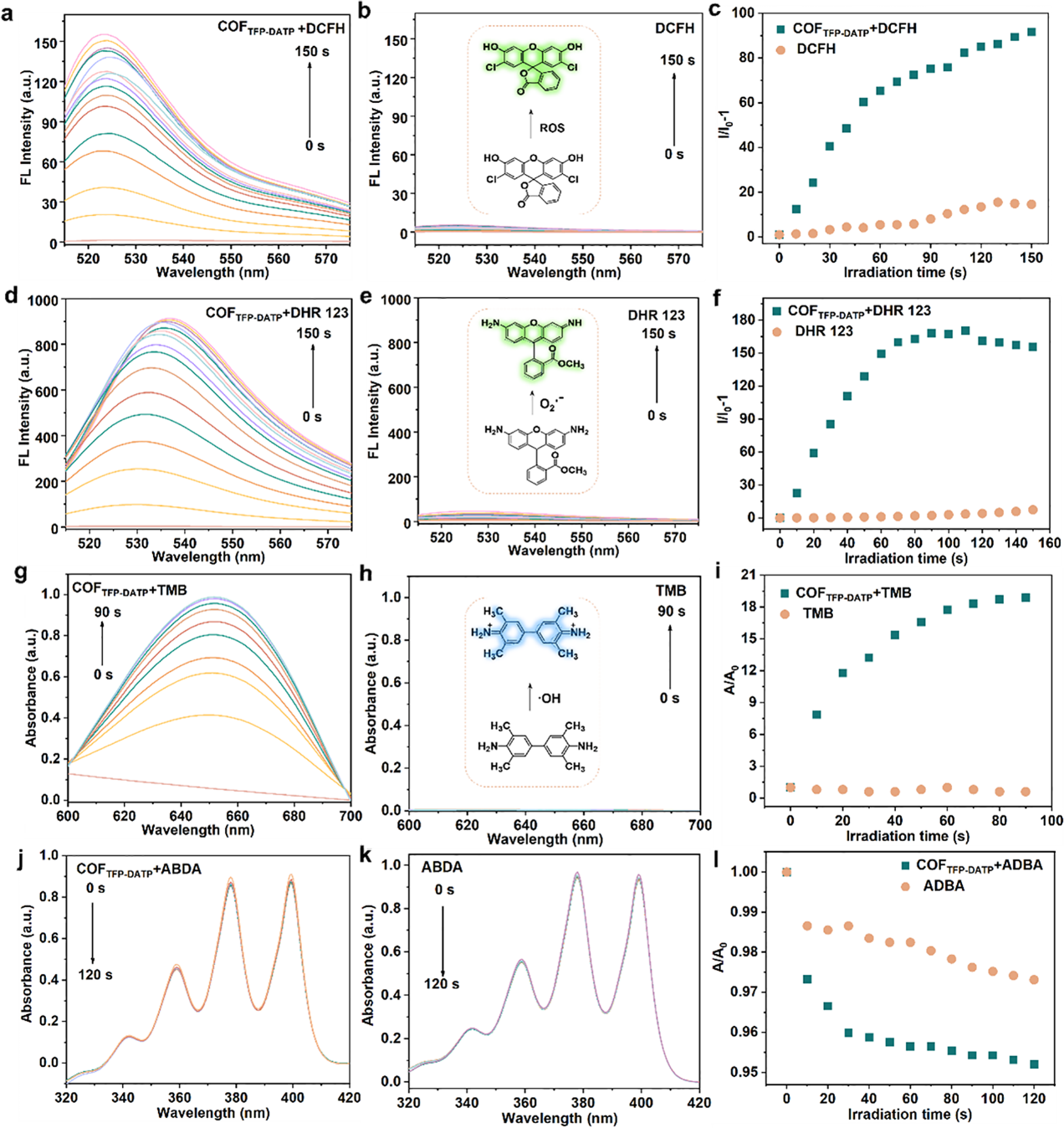
**

**Fig. S13** **Characterization of the photodynamic performance of COF_TFP-DATP_ in PBS.** (a) ROS generation of COF_TFP-DATP_ (10 μg/mL) upon light irradiation using DCFH (100 μM) as an indicator. (d) ROS generation of COF_TFP-DATP_ (10 μg/mL) upon light irradiation using DHR123 (100 μM) as an indicator. (g) Absorbance decay of TMB (100 μM) in the presence of COF_TFP-DATP_ (10 μg/mL) under light irradiation. (b, e, h) The control group was the same test conditions without COF_TFP-DATP_. The insets in Fig. S10b, e, g show the structure of the corresponding probes as well as the structural changes when indicated. Absorption spectra of ABDA (100 μM) in the (j) presence and (k) absence of COF_TFP-DATP_ (10 μg/mL) under light irradiation. The fluorescence emission spectrum changes of (c) DCFH at 525 nm and (f) DHR 123 at 535 nm and the absorbance changes of (i) TMB at 655 nm and (j) ABDA at 378 nm as a function of irradiation time in the presence of COF_TFP-DATP_. The time interval of the test is 10s and the white light irradiation power is 26 mW cm^−2^.

**
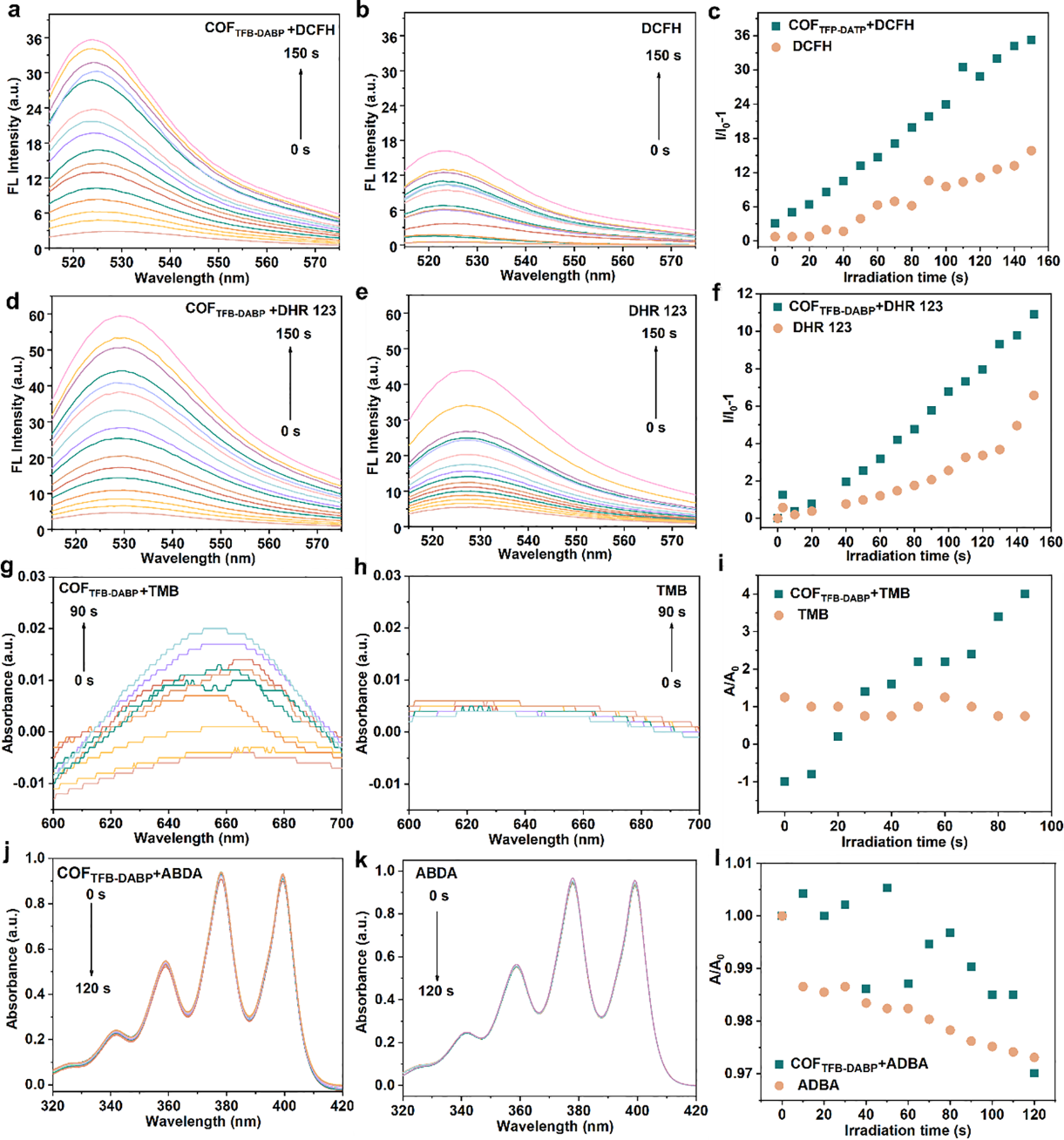
**

**Fig. S14 Characterization of the photodynamic performance of** **COF_TFB-DABP_ in PBS.** (a) ROS generation of COF_TFB-DABP_ (10 μg/mL) upon light irradiation using DCFH (100 μM) as an indicator. (d) ROS generation of COF_TFB-DABP_ (10 μg/mL) upon light irradiation using DHR123 (100 μM) as an indicator. (g) Absorbance decay of TMB (100 μM) in the presence of COF_TFB-DABP_ (10 μg/mL) under light irradiation. (b, e, h) The control group was the same test conditions without COF_TFB-DABP_. The insets in Fig. S11b, e, g show the structure of the corresponding probes as well as the structural changes when indicated. Absorption spectra of ABDA (100 μM) in the (j) presence and (k) absence of COF_TFB-DABP_ (10 μg/mL) under light irradiation. The fluorescence emission spectrum changes of (c) DCFH at 525 nm and (f) DHR 123 at 535 nm and the absorbance changes of (i) TMB at 655 nm and (j) ABDA at 378 nm as a function of irradiation time in the presence of COF_TFB-DABP_. The time interval of the test is 10s and the white light irradiation power is 26 mW cm^−2^.


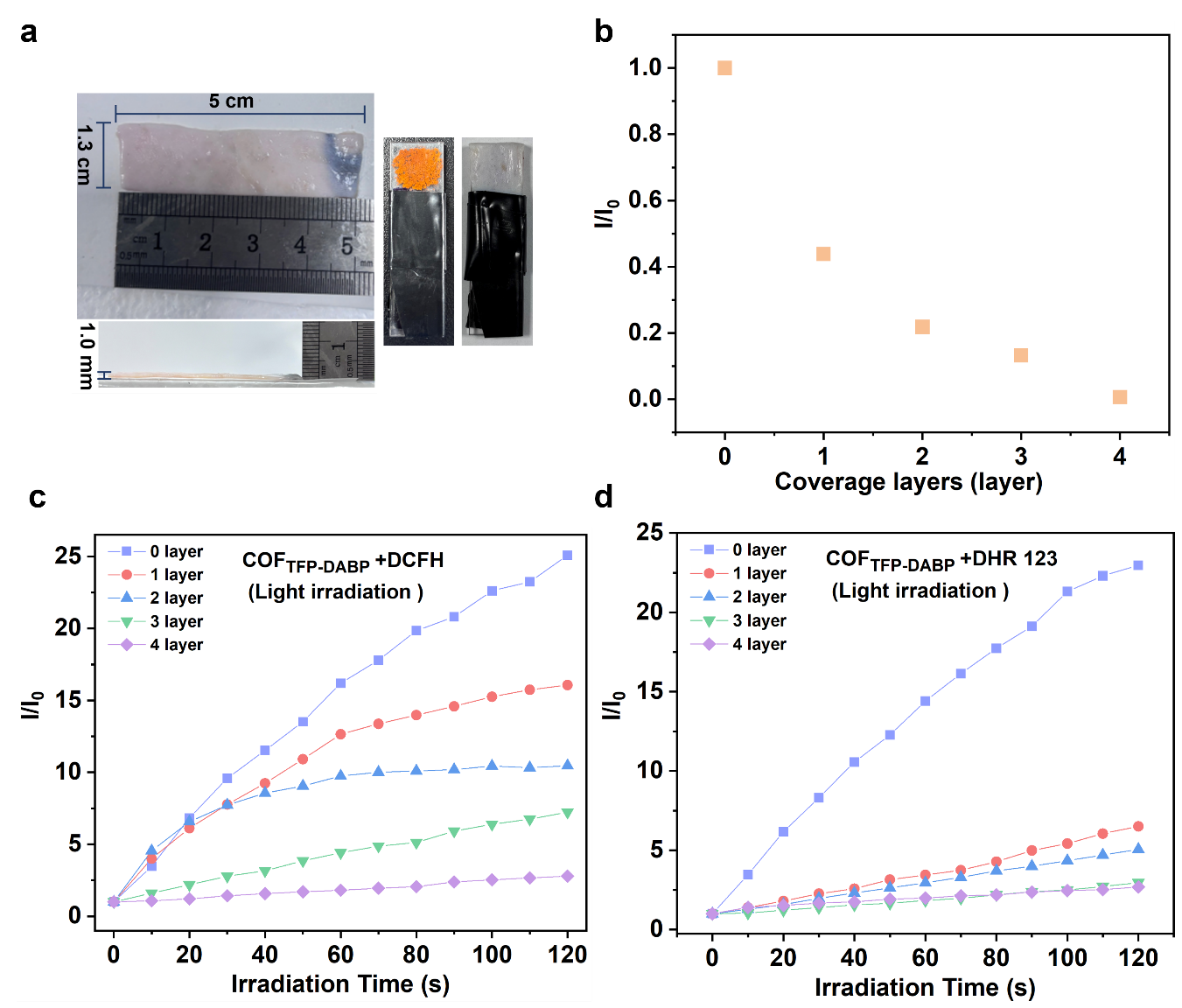


**Fig. S15** (a) Display of the length, width, and thickness of mouse skin and the method of fixing COF_TFP-DABP_ powder on a solid quartz plate, as well as the method of wrapping mouse skin with a quartz plate. (b) The fluorescence intensity of COF_TFP-DABP_ changes after wrapping different layers of mouse skin (λex=400nm, λem=650 nm). (c) Using DCFH (100 μM) as an indicator, the ROS production of COF_TFP-DABP_ (10 μg/mL) after wrapping 0-4 layers of mouse skin under light irradiation. (d) Using DHR 123 (100 μM) as an indicator, the ROS production of COF_TFP-DABP_ (10 μg/mL) wrapped in 0-4 layers of mouse skin under light irradiation.

We explored the penetration of light into tissues through two experiments in our preliminary research. We collected the outer skin of mice that had completed the experiment and were euthanized during the experiment. After freezing and hardening at -20℃, they were cut flat into 5cm×1.3cm long square strips with a thickness of approximately 1mm (Fig. S15a). Place the COF_TFP-DABP_ powder in a solid quartz plate and fix it with black tape. Use a fluorescence spectrophotometer to test the fluorescence intensity changes of COF_TFP-DABP_ under different layers of mouse skin obstruction. As the full spectrum white light was used in this experiment, the excitation wavelength selected was 400 nm in the white light range. The collected fluorescence emission peak intensity changes at 650 nm. As shown in Fig. S15b, As the number of layers of mouse skin increases, the fluorescence intensity becomes weaker. The fluorescence quenching rates of 1-4 layers of mouse skin are 56.16%, 78.16%, 86.77%, and 99.33%, respectively. The most important reason for using this model is that liquid colorimetric dishes cannot be fixed on liquid scaffolds for testing after being wrapped in 1-4 layers of mouse skin. Therefore, solid scaffolds and solid powders are selected for fluorescence testing to explore the penetration of light. The ultimate goal of studying the penetrability of light is to demonstrate that COF_TFP-DABP_ can effectively produce reactive oxygen species within a certain depth of tissue. Therefore, we designed another model to investigate the effect of different tissue depths on the production of reactive oxygen species by light irradiated COF_TFP-DABP_. The specific experimental details are as follows: DCFH and DHR 123 are used as ROS indicators respectively (Fig. S15c and Fig. S15d), added to the aqueous solution of COF_TFP-DABP_, and the characteristic peaks at 580 nm of DCFH and DHR 123 are collected at an excitation wavelength of 489 nm to evaluate the ROS generation performance of COF_TFP-DABP_. A set of data is measured every 10 seconds of illumination. Similarly, in the experimental group, 1-4 layers of 5×5 cm mouse skin were fully wrapped around a liquid colorimetric dish and illuminated to record the test data. As shown in Fig. S15, as the number of layers of mouse skin increases, the light transmission efficiency decreases, and the ROS produced by COF_TFP-DABP_ gradually decreases. After covering 4 layers of skin, the ROS production efficiency of DCFH and DHR123 decreased by 88.68% and 88.31%, respectively, after 120 seconds of light exposure. In this experiment, in order to increase the light transmittance and maximize the ROS generation performance of COF_TFP-DABP_, we adopted the experimental method of directly irradiating the modelling area of in situ transplant tumor model.


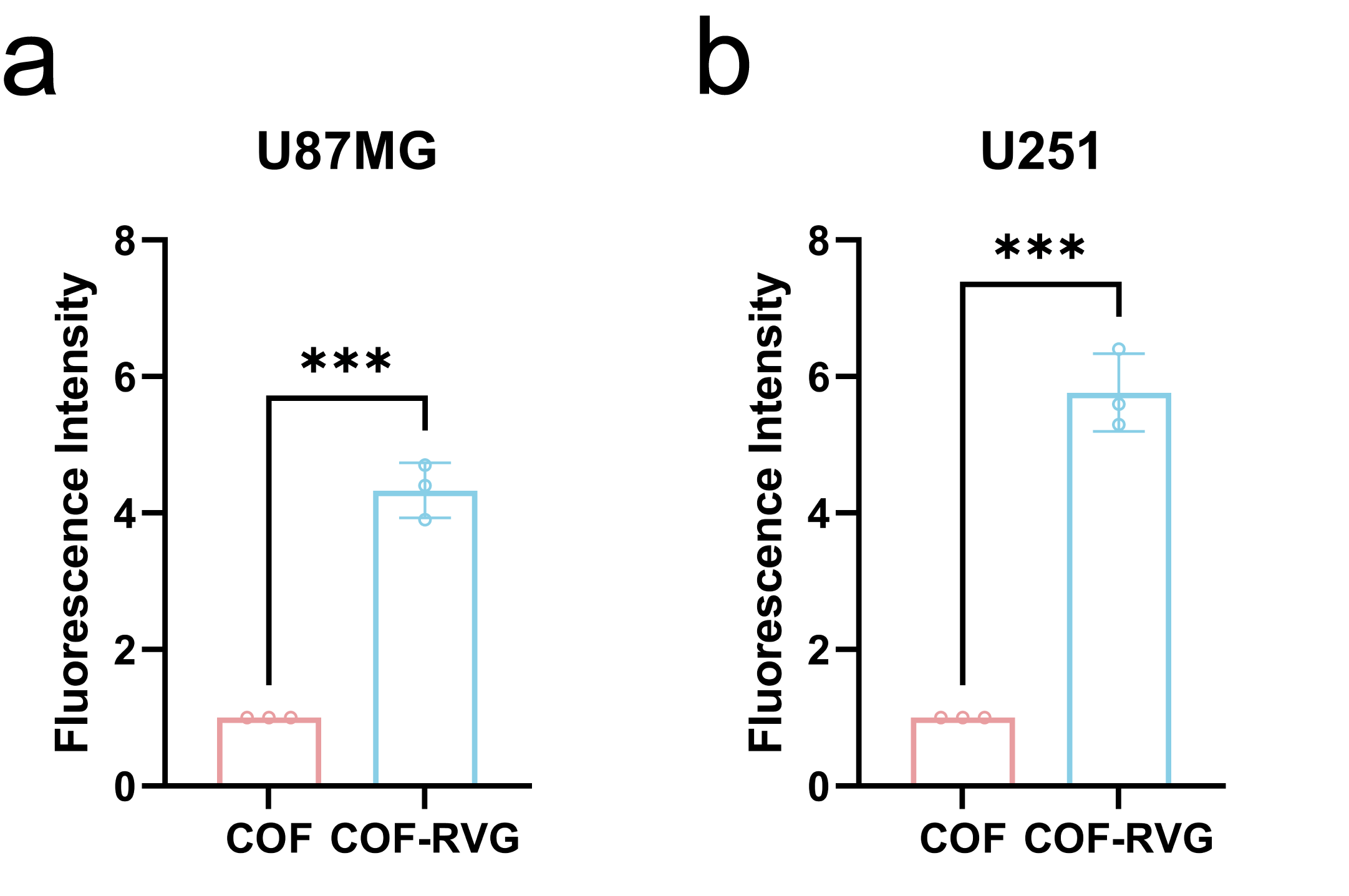


Fig. S16 The total fluorescence intensity of immunofluorescence images of GBM cells incubated with nanoparticles. (cf. **Fig. 4a**)


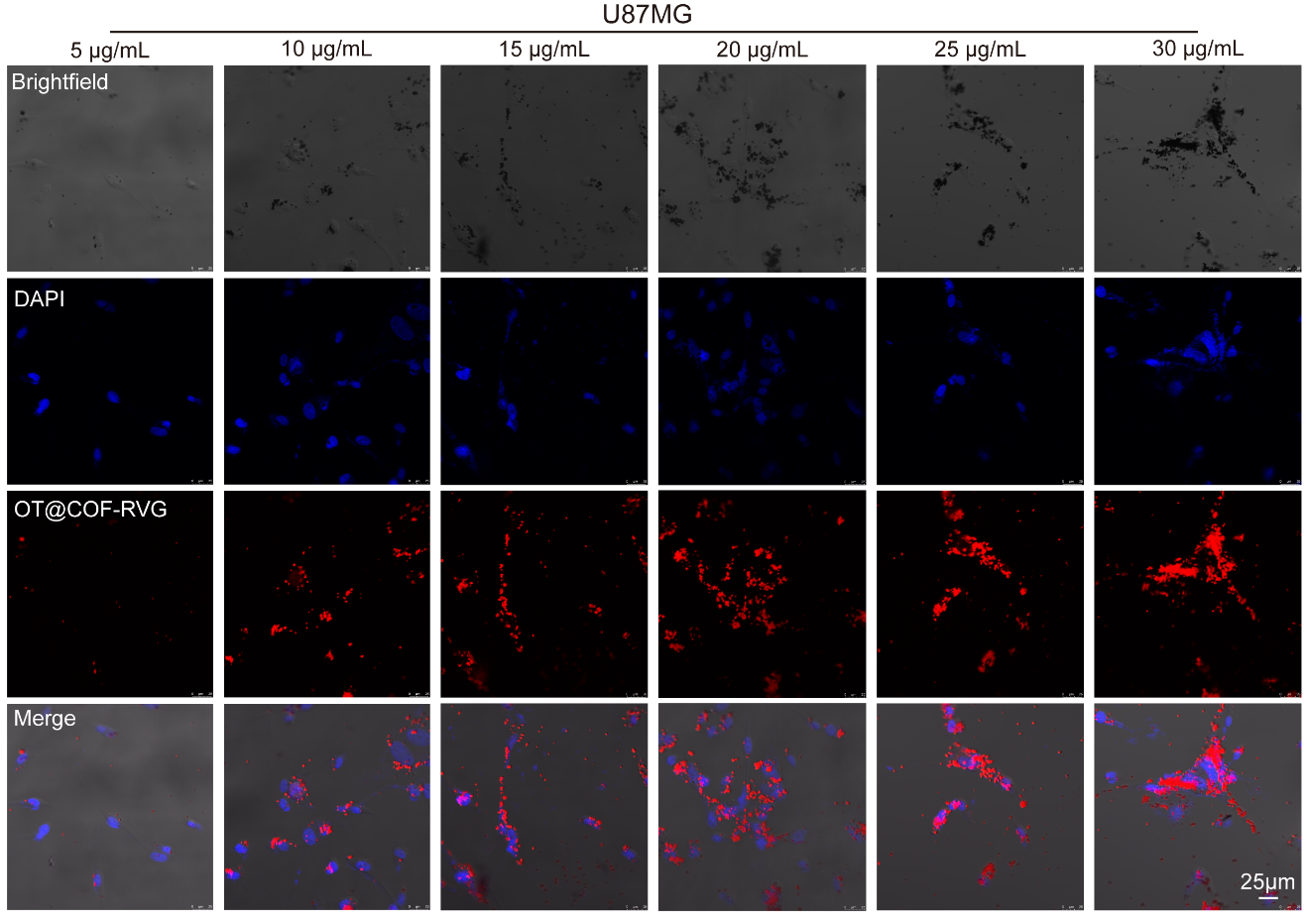
 **Fig. S17.** **Enrichment of COF-RVG in tumor models.** The uptake of U87MG cells incubated with the different concentrations of nanoparticles was evaluated by confocal laser scanning microscope. Cell nucleus was stained with DAPI (blue).


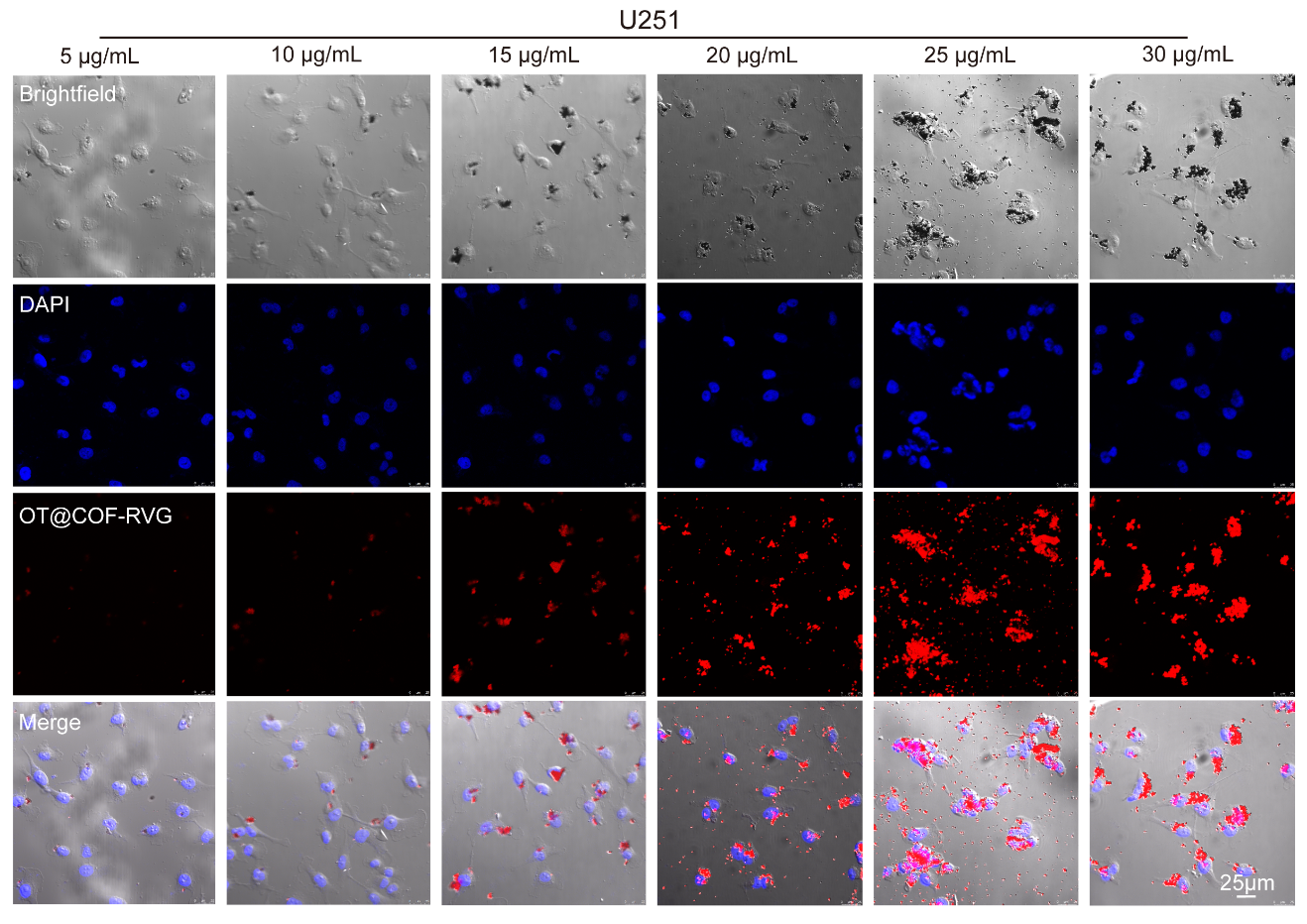
 **Fig. S18 Enrichment of COF-RVG in tumor models.** The uptake of U251 cells incubated with the different concentrations of nanoparticles was evaluated by confocal laser scanning microscope. Cell nucleus was stained with DAPI (blue).


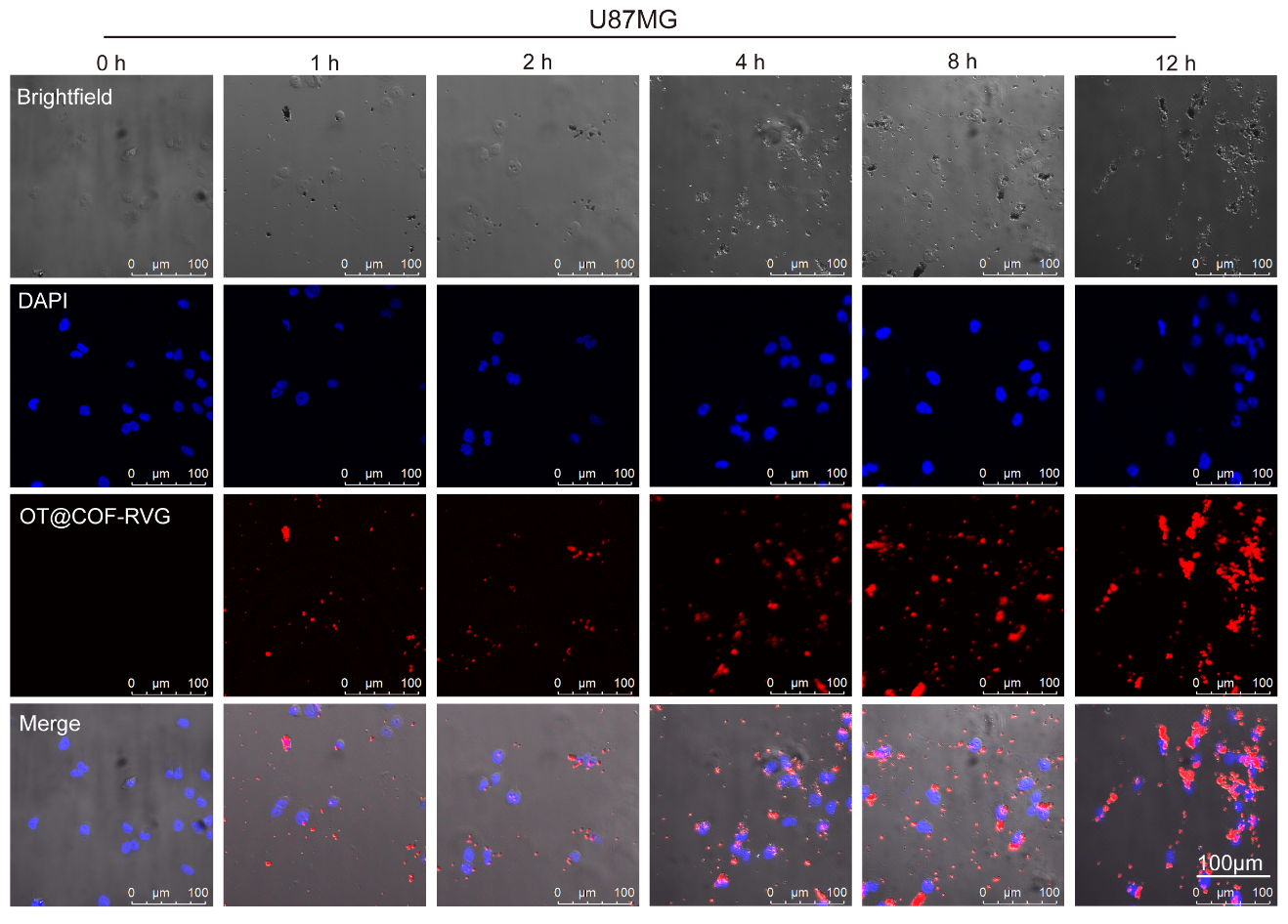


**Fig. S19** **Enrichment of COF-RVG in tumor models.** Confocal laser scanning microscope was used to evaluate the cellular uptake of nanoparticles by U87MG cells after incubation with OT@COF-RVG for different times points. Cell nucleus was stained with DAPI (blue).


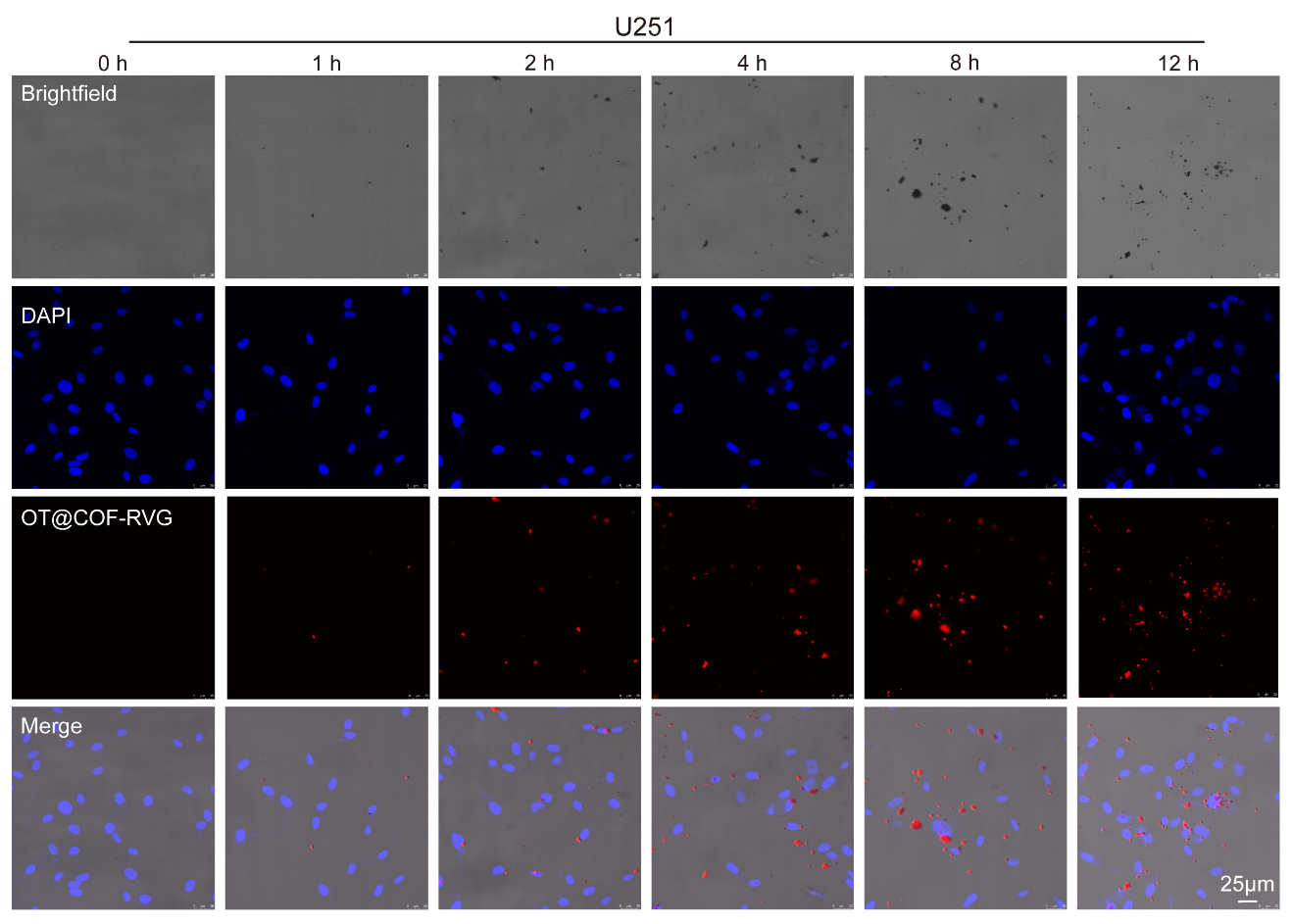


**Fig. S20** **Enrichment of COF-RVG in tumor models.** Confocal laser scanning microscope was used to evaluate the cellular uptake of nanoparticles by U251 cells after incubation with OT@COF-RVG for different times. Cell nucleus was stained with DAPI (blue).


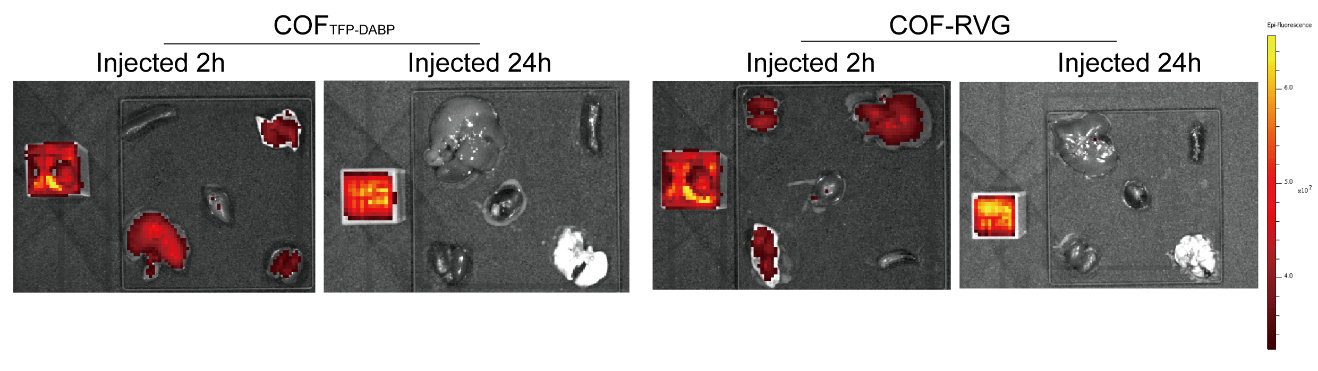
 **Fig. S21** **Enrichment of COF-RVG in tumor models.** The main organs of mice were collected after venously injecting COF_TFP-DABP_ or COF-RVG, and the fluorescence image represented the biological distribution of nanoparticles.


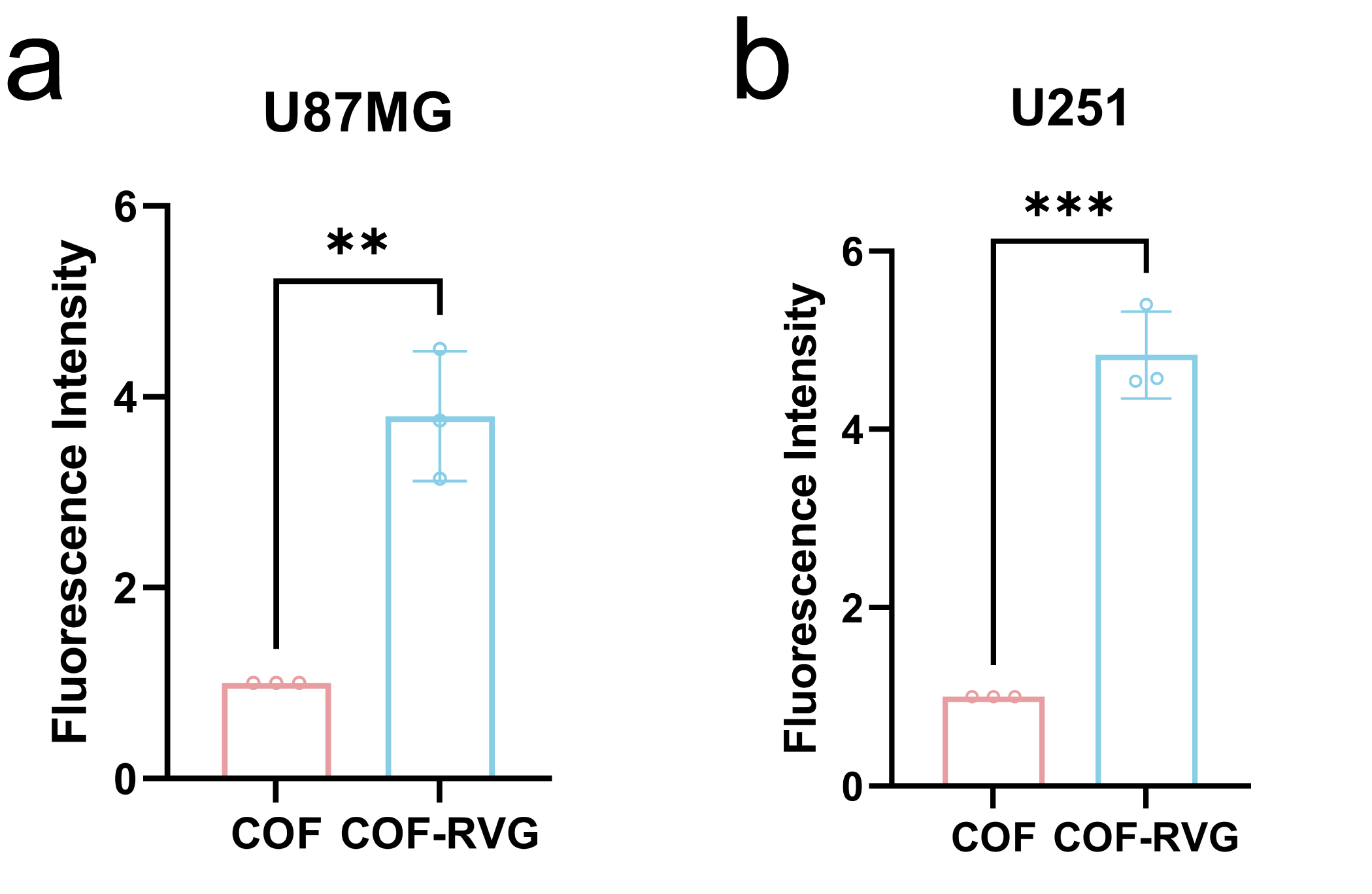


Fig. S22 The total fluorescence intensity of immunofluorescence images of GBM cells incubated with nanoparticles in BBB in vitro cell model. (cf. **Fig 4c)**


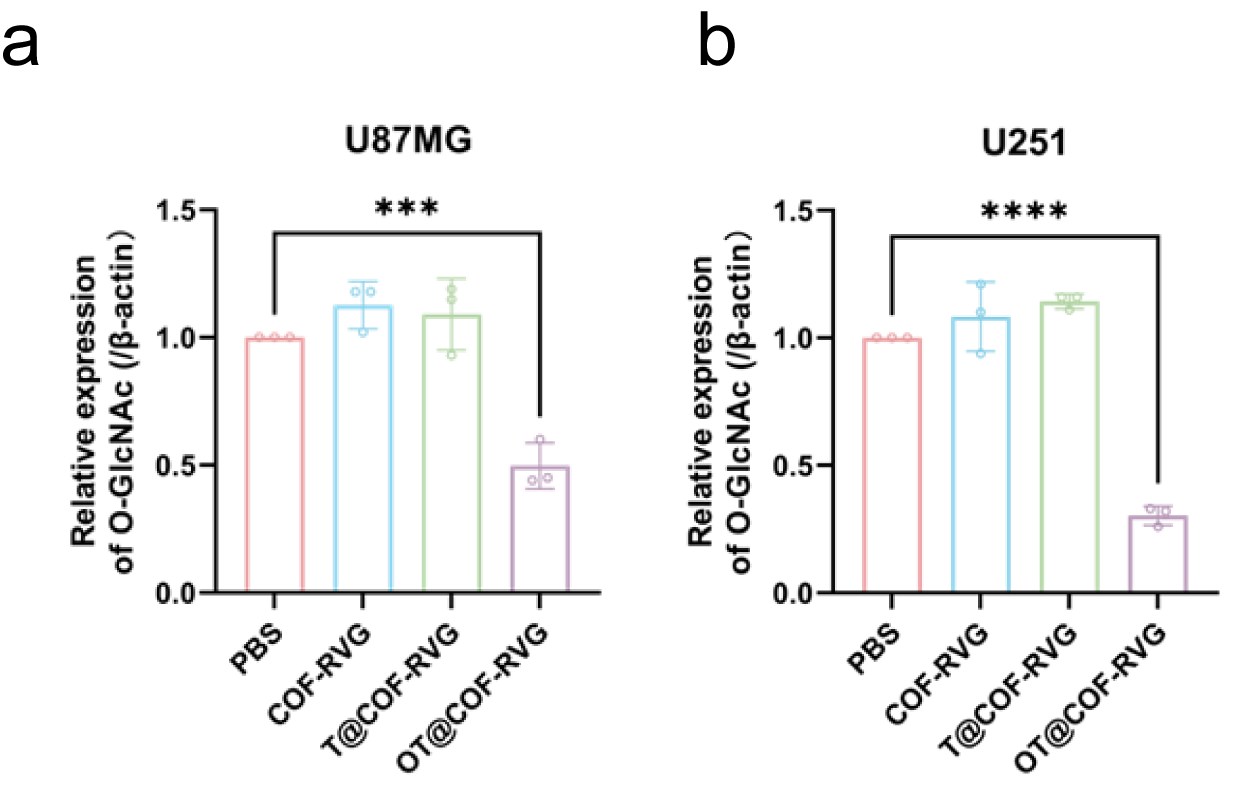


Fig. S23 The total cellular O-GlcNAc expression was determined after different treatments by western blot analysis in (a) U87MG and (b) U251 cells. (cf. **Fig 5b**)


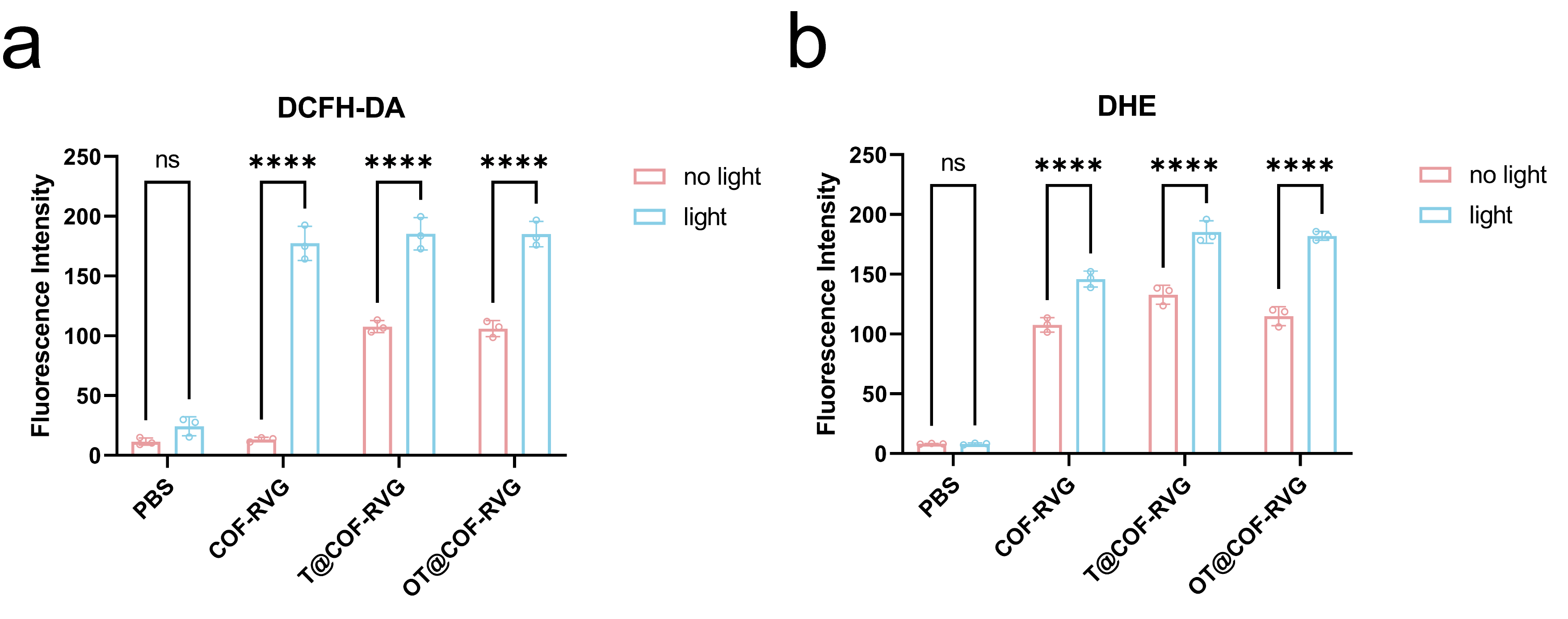


Fig. S24 The quantified fluorescence intensities of DCFH-DA and DHE. (cf. **Fig.5c and Fig.5d**).


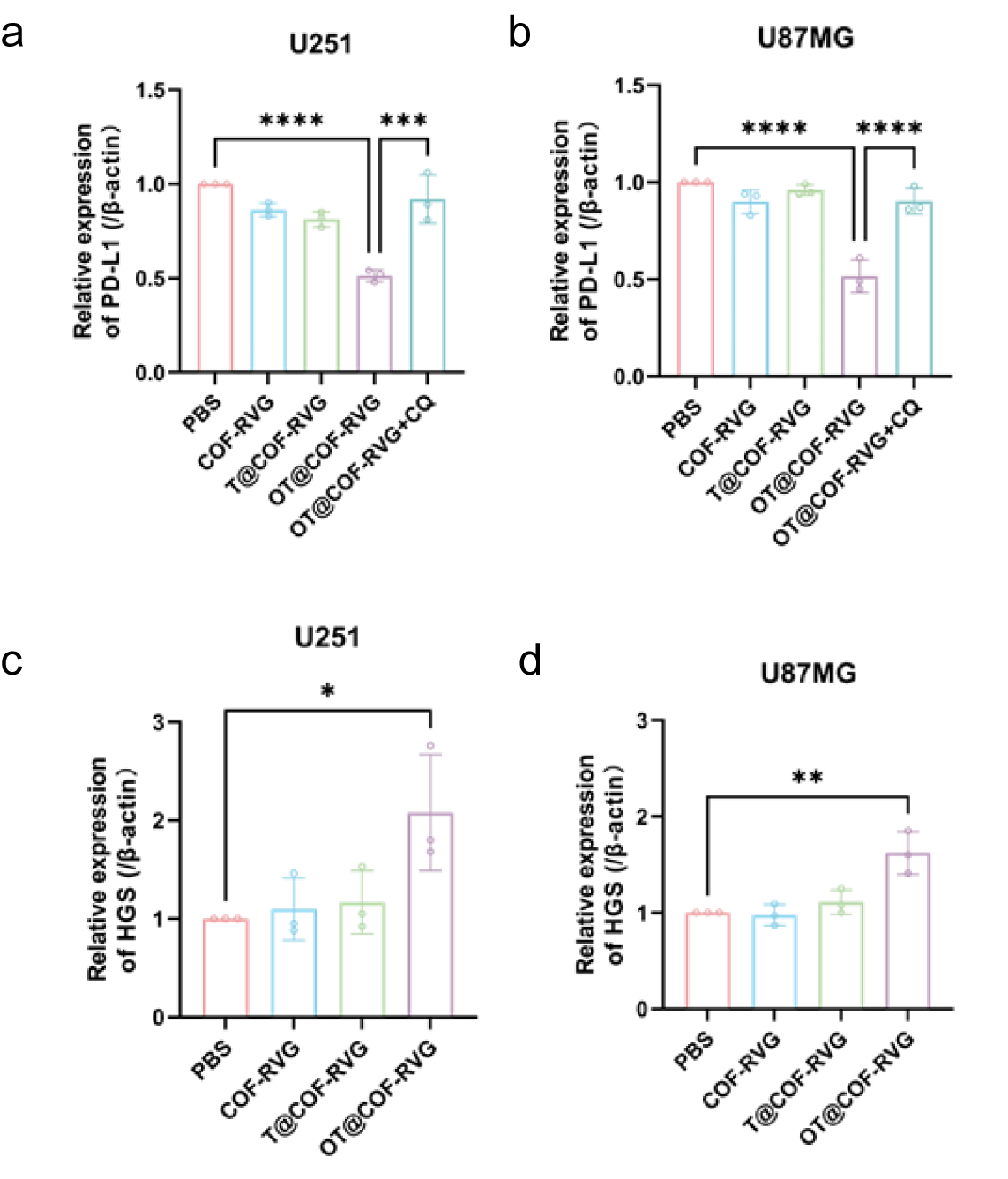


Fig.S25 Western blot analyses of (a) the expression of PD-L1 in U251; (b) the expression of PD-L1 in U87MG; (c) the expression of HGS in U251; and (d) the expression of HGS in U87MG. (cf. **Fig 6e and Fig 6f**).


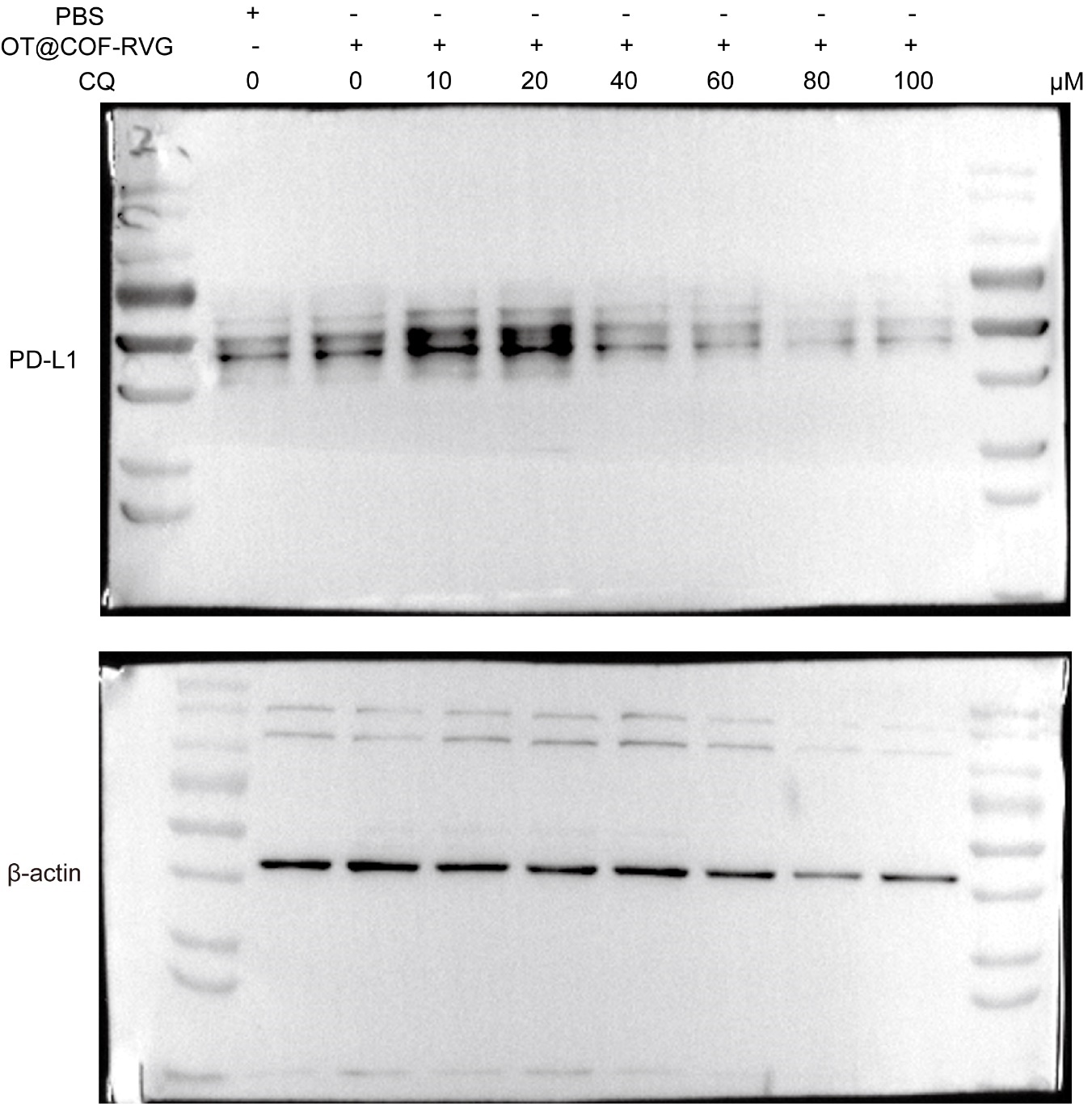


**Fig. S26** **OT@COF-RVG Promote ICD and cause PD-L1 down regulation.** The expression analysis of PD-L1 protein in cells following various treatments was conducted using western blot.


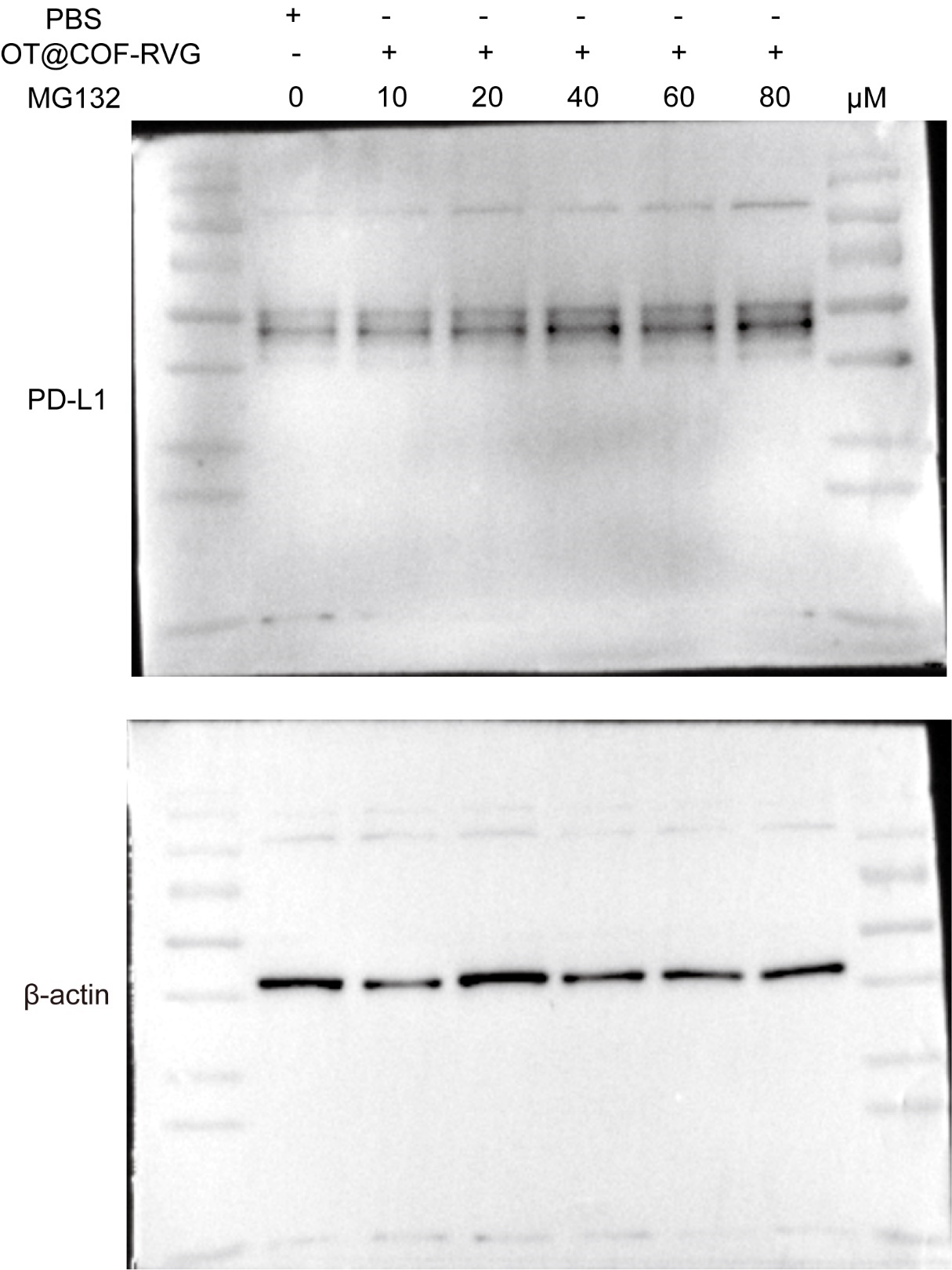


**Fig. S27** **OT@COF-RVG Promote ICD and cause PD-L1 down regulation.** The expression analysis of PD-L1 protein in cells following various treatments was conducted using western blot.


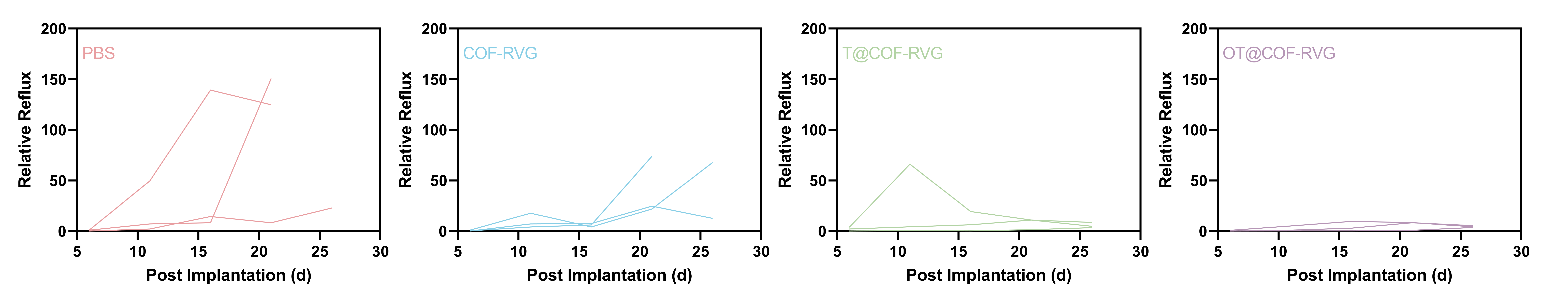


Fig. S28 Quantified luminescence levels of mice using the LivingImage. (n = 3 mice) (cf. **Fig. 7c**).


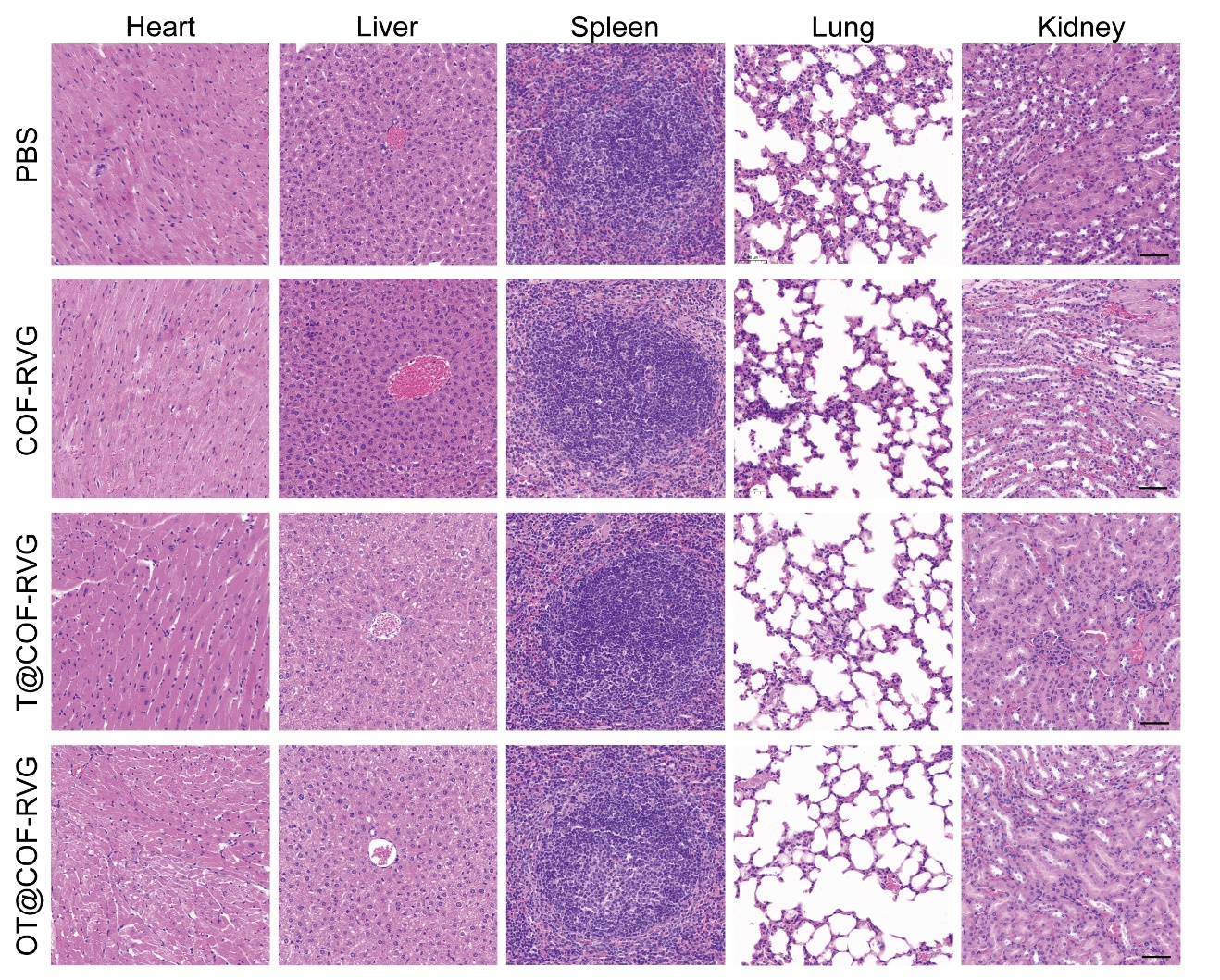


**Fig. S29** **In vivo biosafety of nanoparticles.** HE staining was employed to identify pathological abnormalities in main organs (including heart, liver, spleen, lung, and kidney) of mice post three days of tail vein administration of different NPs.


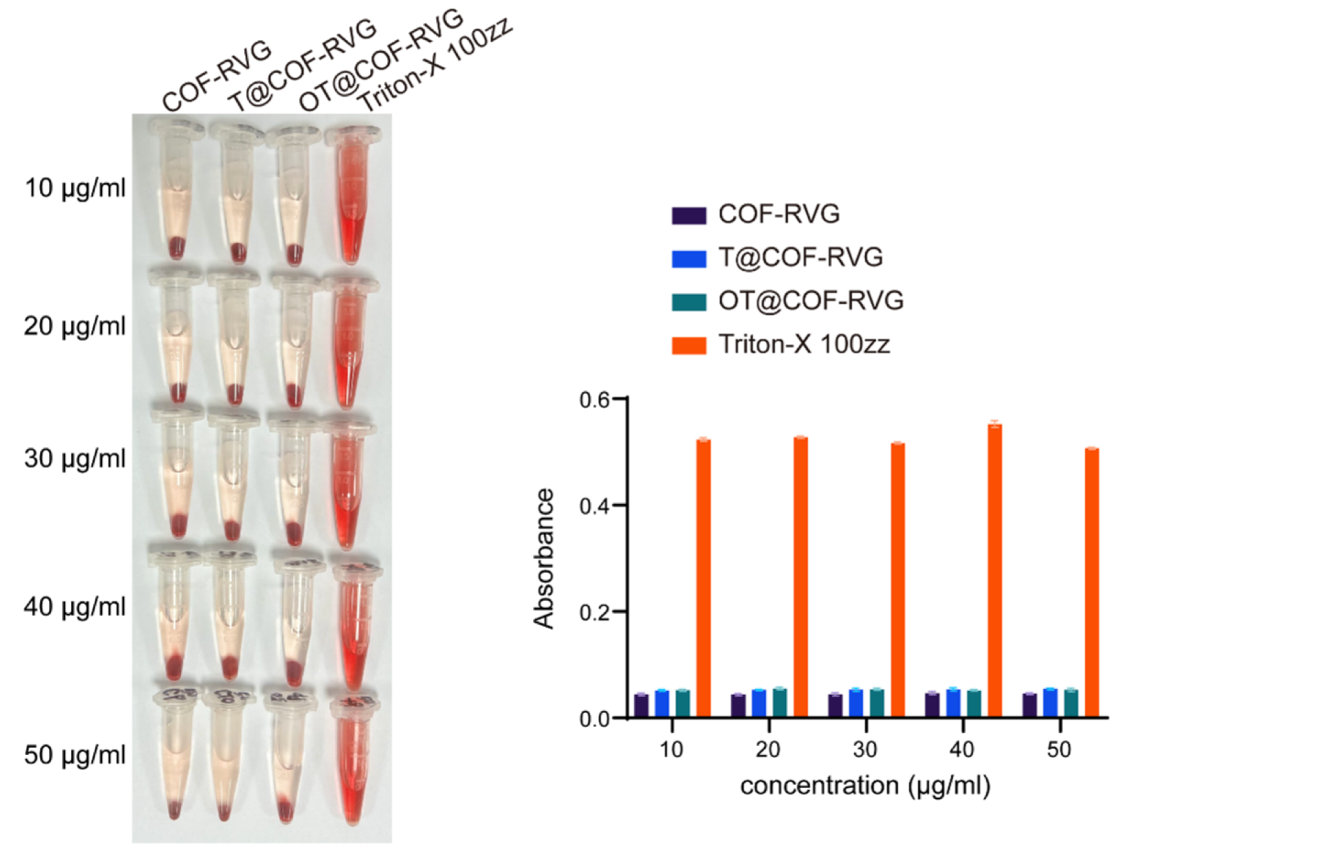
 **Fig. S30** **Haemolysis assay of nanoparticles.** Hemolysis results and quantitative analysis of hemoglobin content of supernatants following the introduction of various NPs.


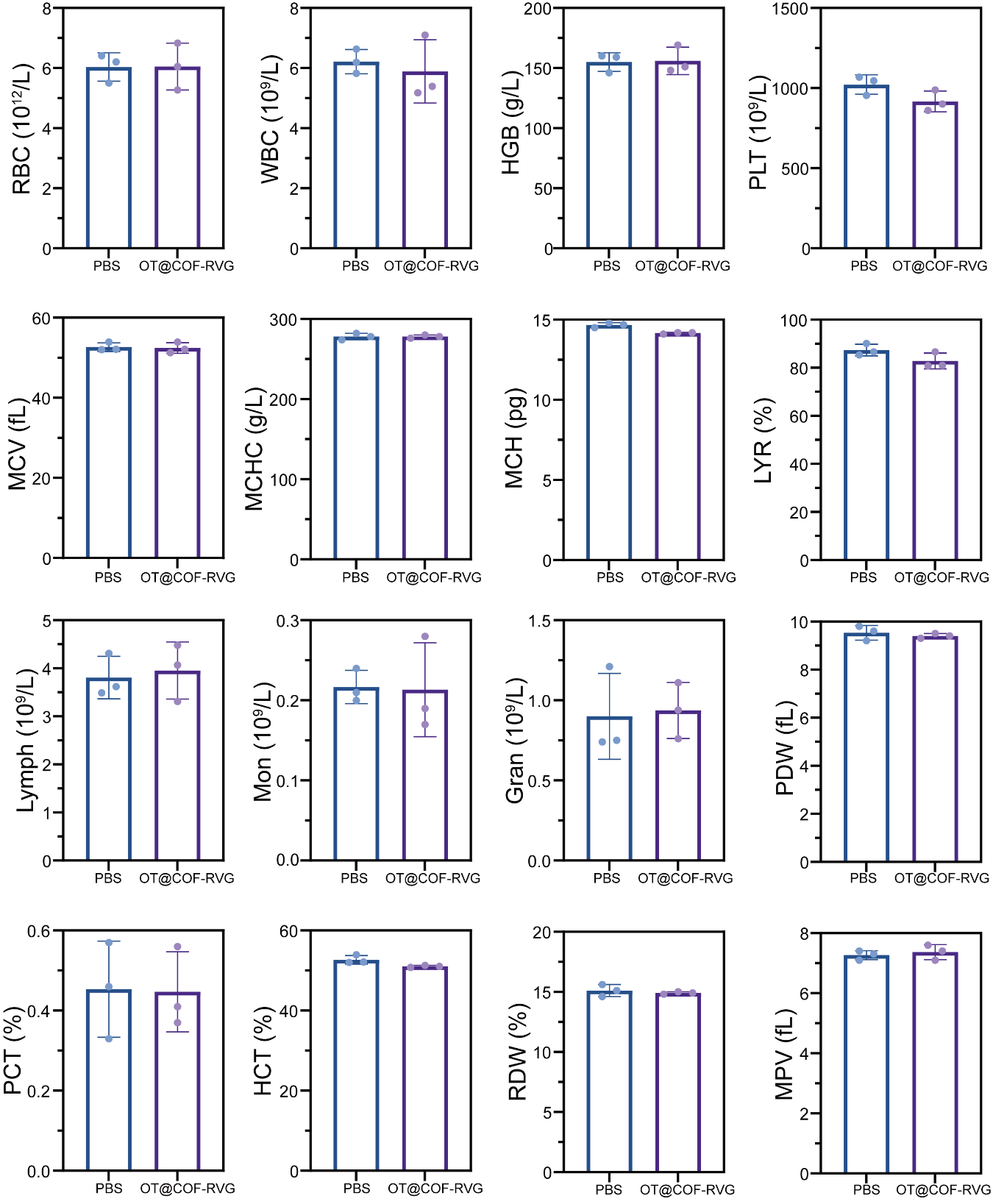


**Fig. S31** **Hematological experiments.** Hematological experiments were conducted to assess alterations in various hematological parameters in mice following the administration of different NPs via tail vein injection. Red blood cell count (RBC), white blood cell count (WBC), hemoglobin concentration (HGB), mean corpuscular volume (MCV), platelet count (PLT), mean corpuscular hemoglobin (MCH), mean corpuscular hemoglobin concentration (MCHC), lymphocyte rate (LYR), Monocytes (Mon), Platelet distribution width (PDW), procalcitonin (PCT), hematocrit (HCT), red blood cell distribution width (RDW), mean platelet volume (MPV).


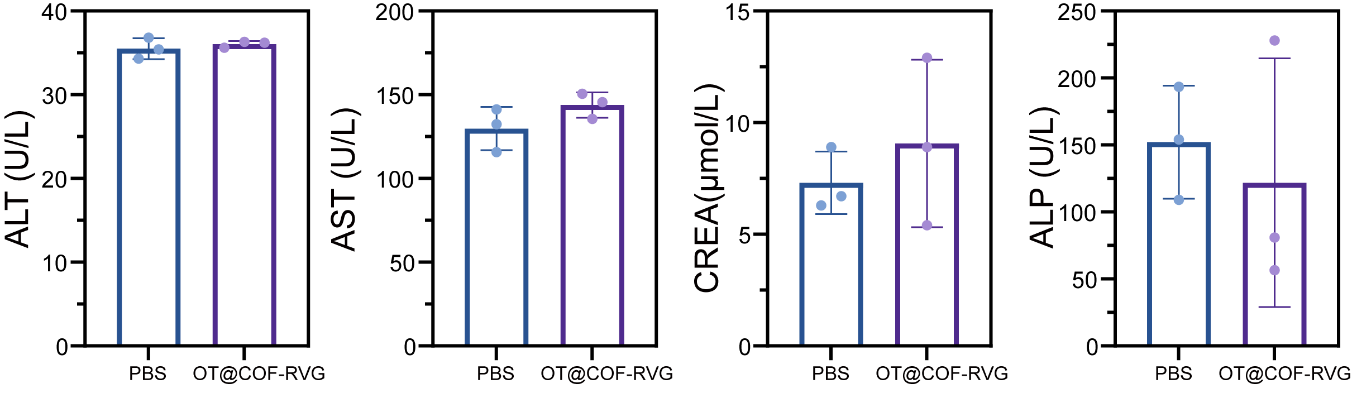


**Fig. S32** **Blood biochemistry experiments.** Blood biochemistry experiments were conducted to assess alterations in various liver and kidney function parameters in mice following the administration of different NPs via tail vein injection. Alanine aminotransferase (ALT), aspartate aminotransferase (AST), Serum creatinine (CREA), alkaline phosphatase (ALP).


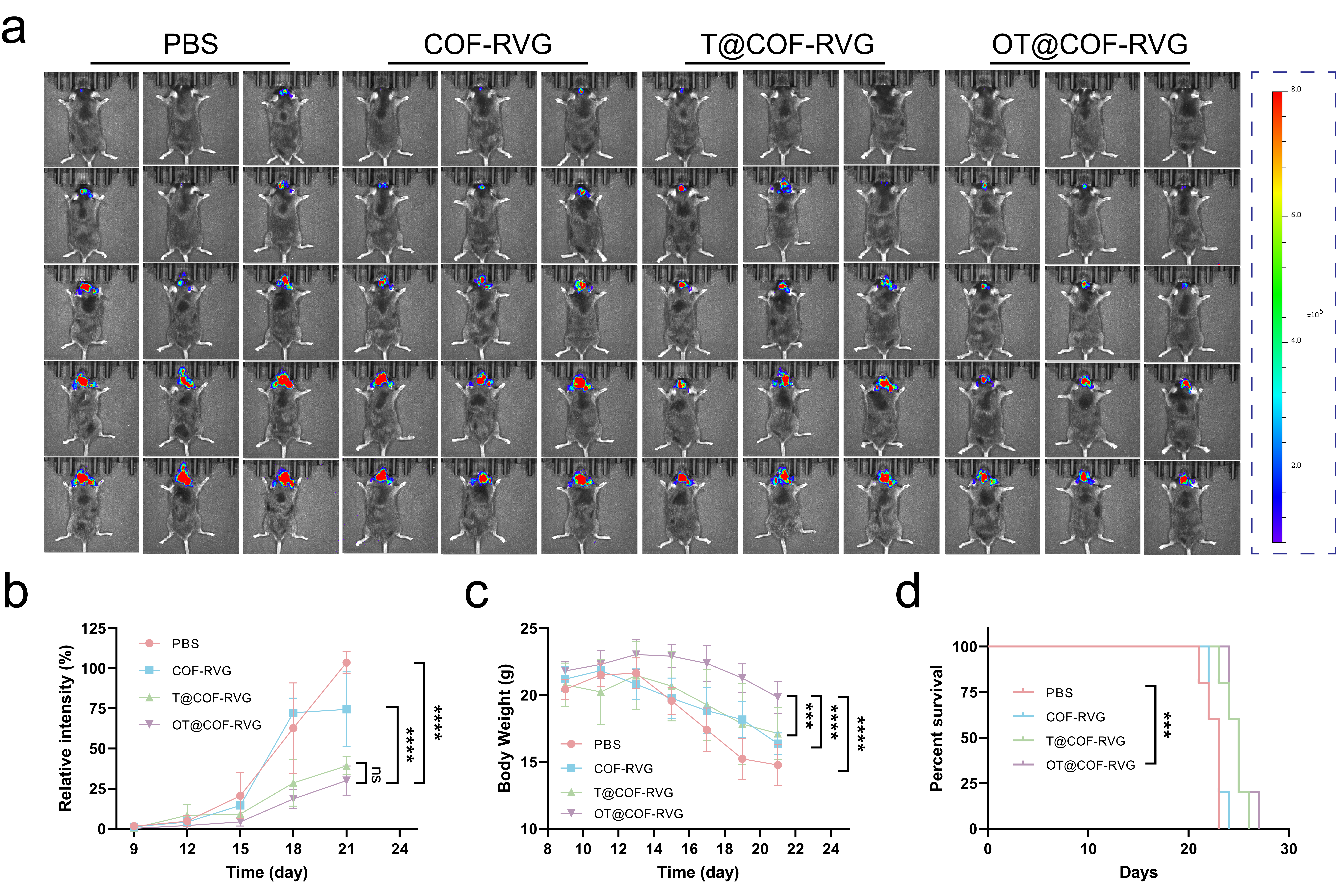


**Fig. S33** Assessment of therapeutic efficiency of OT@COF-RVG. (a) Bioluminescence images of GL261 tumor-bearing mice. (b) relative bioluminescence intensities of whole brains in various treatment groups. (c) Body weight changes for control and treated GL261 tumor-bearing mice. (d) Survival rate of GL261 tumor-bearing mice after received different treatments. Data are presented as the mean ± SD (n = 3 mice per group; ^ns^p>0.05, *p < 0.05, **p < 0.01, ***p < 0.001, ****p < 0.0001 by log-rank test).


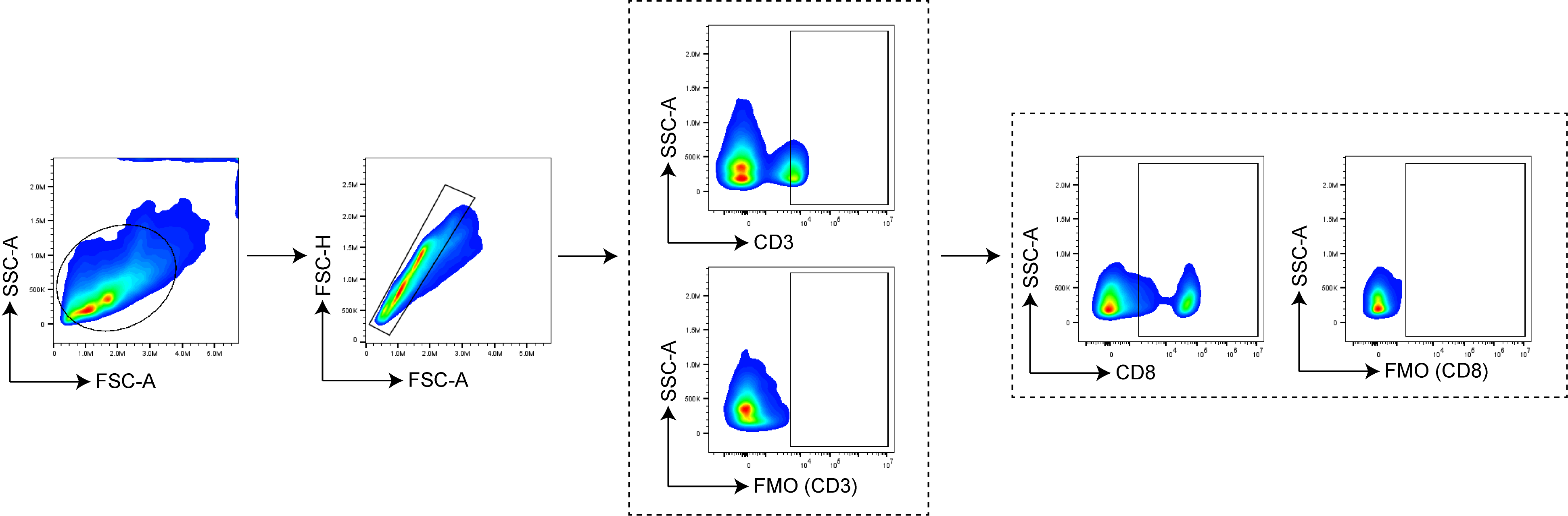


**Fig. S34** The utilization of a gating strategy for the purpose of sorting CD8^+^ T cells (CD8^+^CD3^+^).


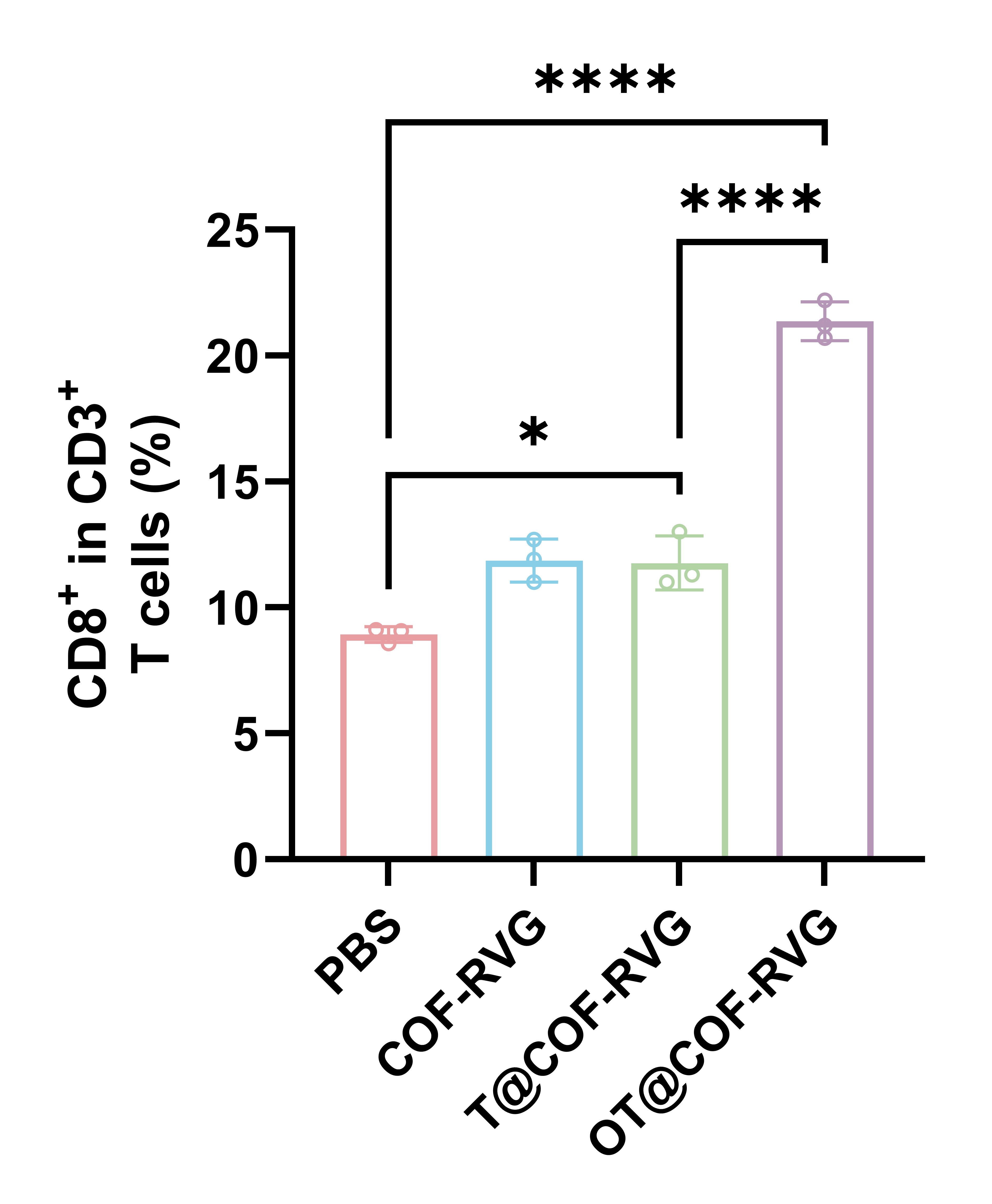


**Fig. S35** Relative quantification of T cells (CD8^+^CD3^+^) (n = 3 mice). (cf. **Fig.8c**). Statistical significance was calculated through one-way ANOVA using a Tukey post-hoc test.


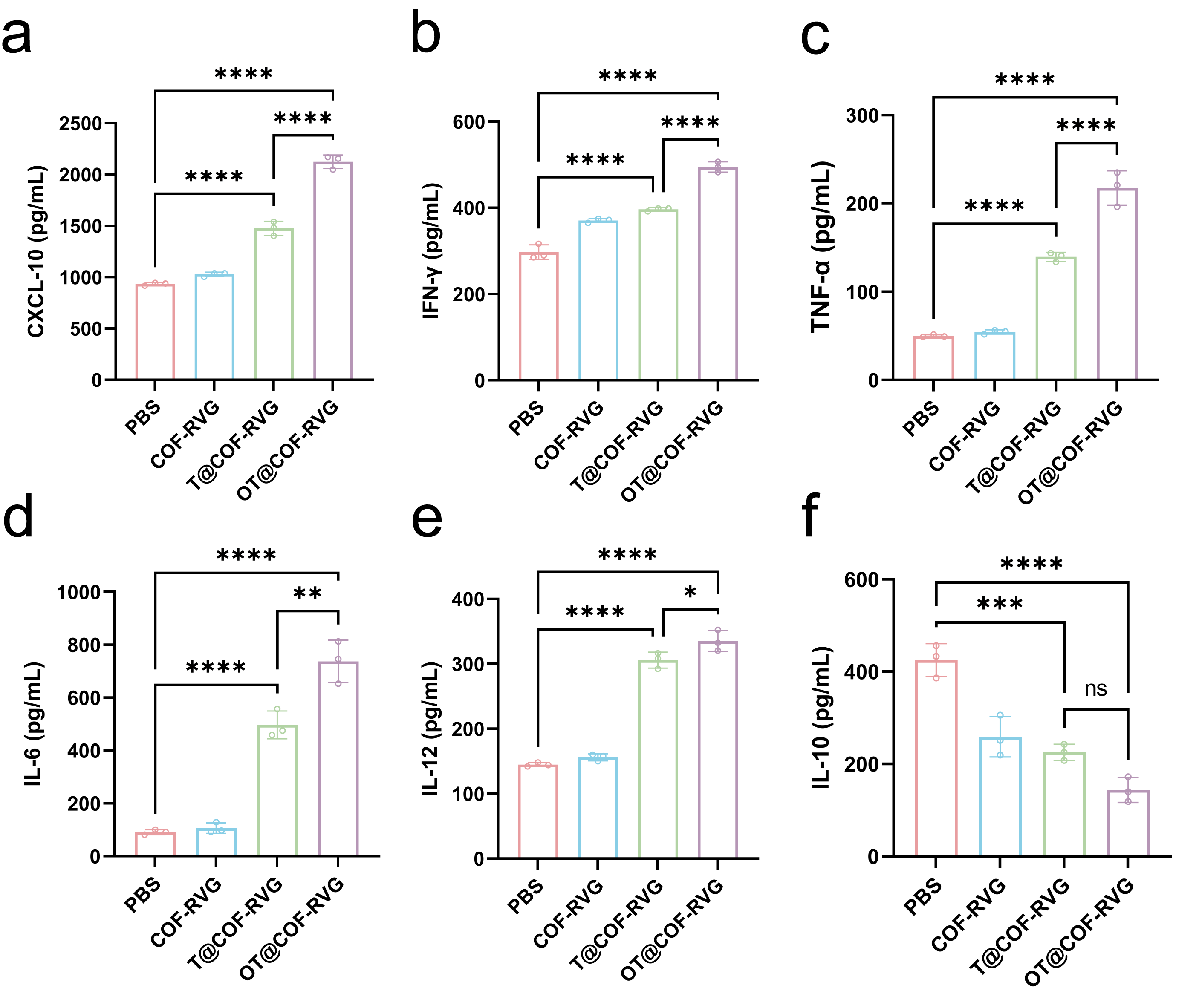


**Fig. S36** Quantification of cytokines secreted by mouse tumor tissues after treatment with different conditions. (a-e) The secretion of proinflammatory cytokines of tumor supernatant in GL261-luc tumor model (n=3 mice), (a) CXCL-10, (b) IFN-γ, (c) TNF-α, (d) IL-6 and (e) IL-12. (f) The secretion of anti-inflammatory cytokines of tumor supernatant in GL261-luc tumor model (n=3 mice), (f) IL-10. Statistical significance was calculated through one-way ANOVA using a Tukey post-hoc test.


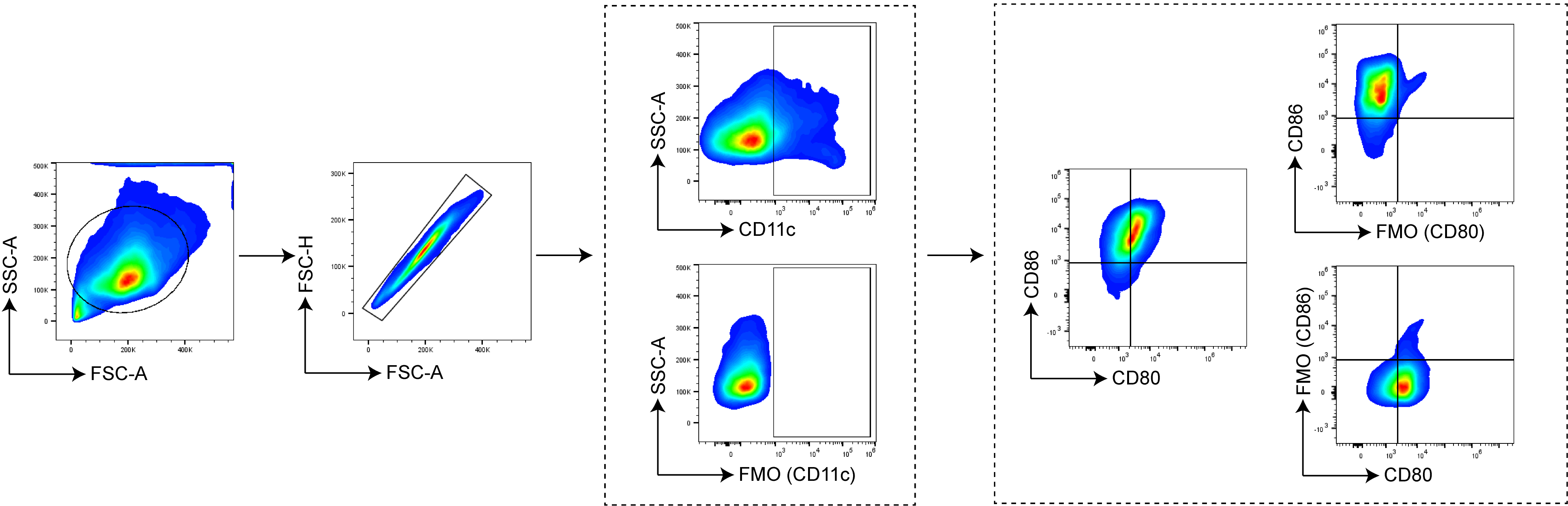


**Fig. S37** The utilization of a gating strategy for the purpose of sorting mDCs (CD86^+^CD80^+^CD11c^+^).


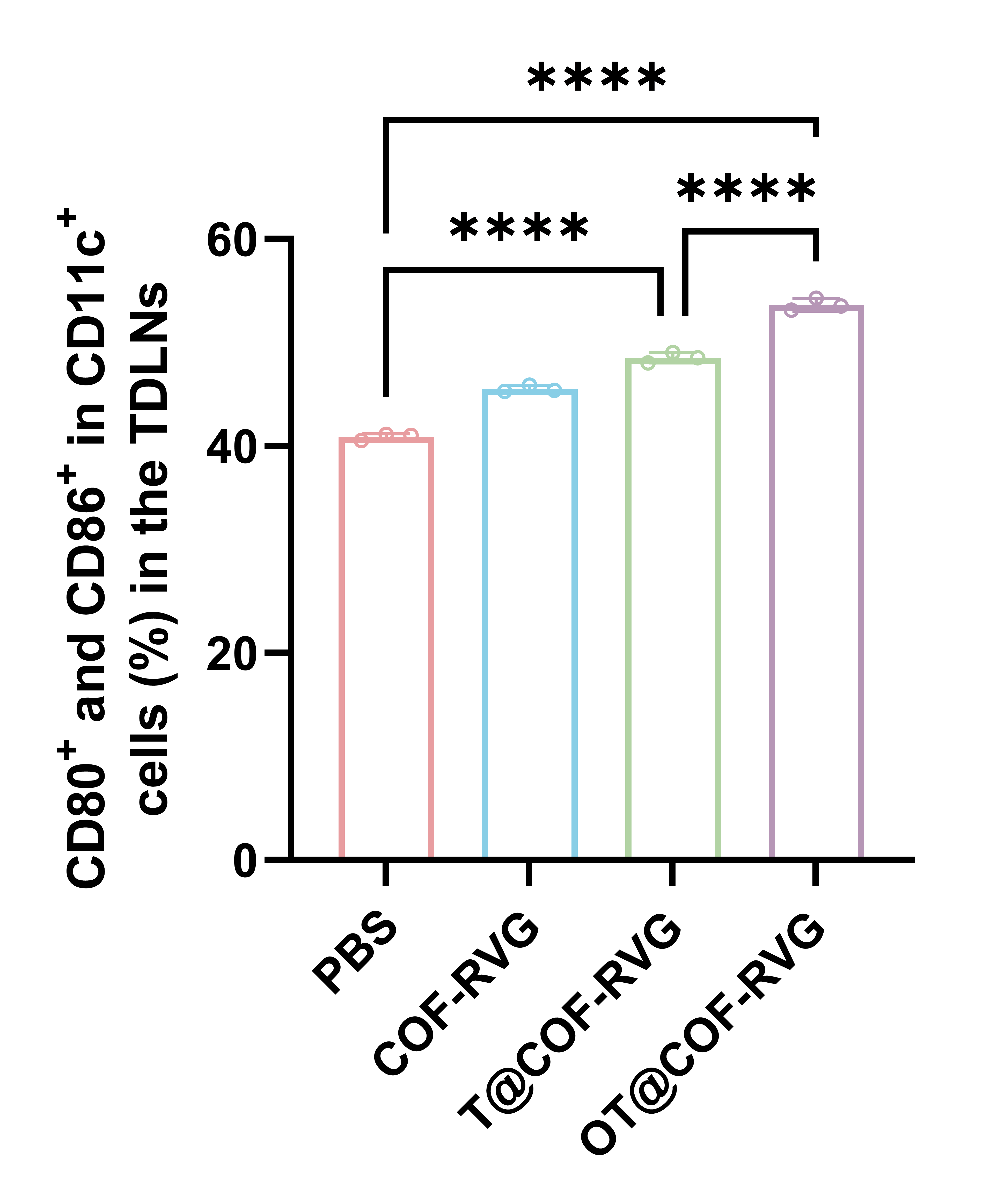


**Fig. S38** Relative quantification of DC maturation (CD86^+^CD80^+^CD11c^+^) (n = 3 mice). (cf. **Fig.8d**). Statistical significance was calculated through one-way ANOVA using a Tukey post-hoc test.


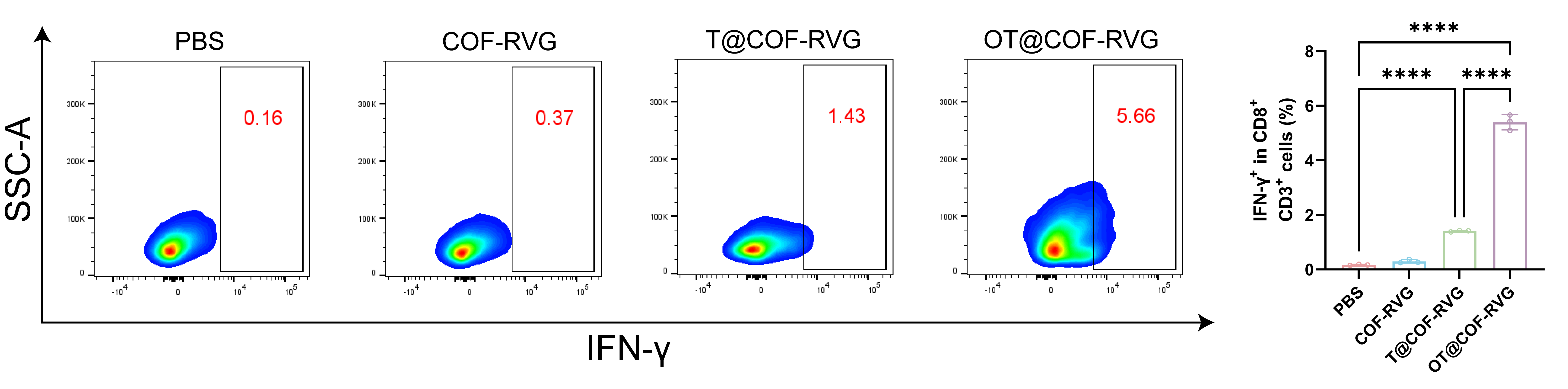


**Fig. S39** Flow cytometric assay and relative quantification of IFN-γ staining of CD8^+^ T cells in spleen in GL261-luc tumor model (n = 3 mice). Statistical significance was calculated through one-way ANOVA using a Tukey post-hoc test.


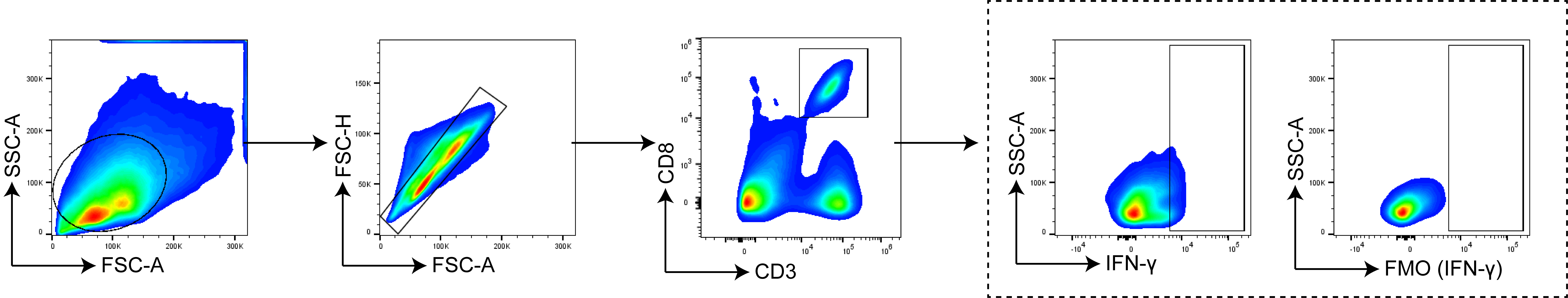


**Fig. S40** Gating strategy and fluorescence minus one (FMO) control for the flow cytometry analysis of IFN-γ specific CD8^+^ T cells in spleen (cf. **Fig. S39**).


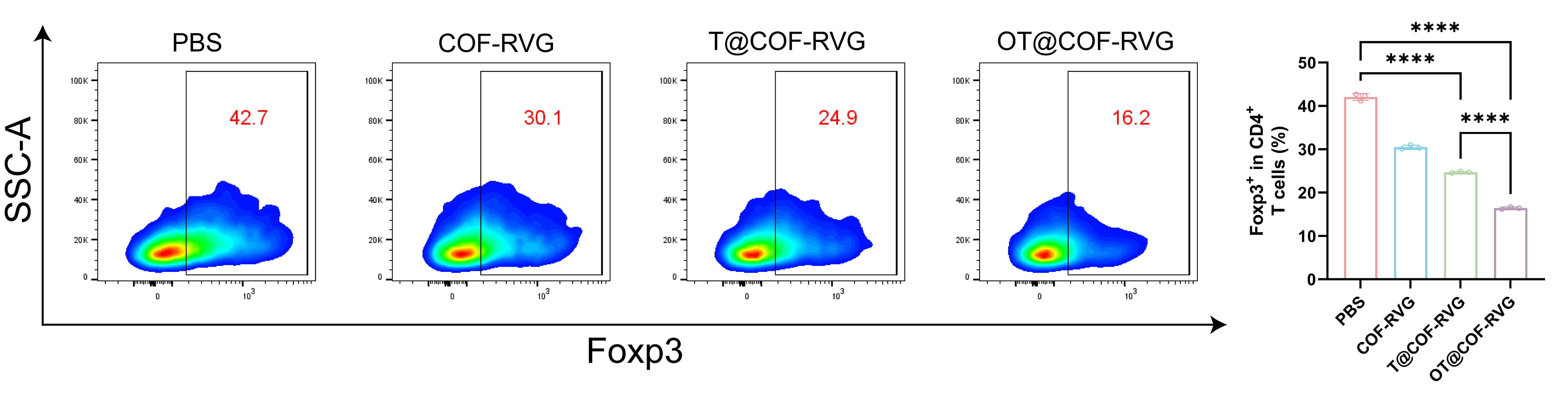


**Fig. S41** Flow cytometric assay of spleen CD4^+^ Foxp3^+^ Tregs (n = 3 mice). Statistical significance was calculated through one-way ANOVA using a Tukey post-hoc test.


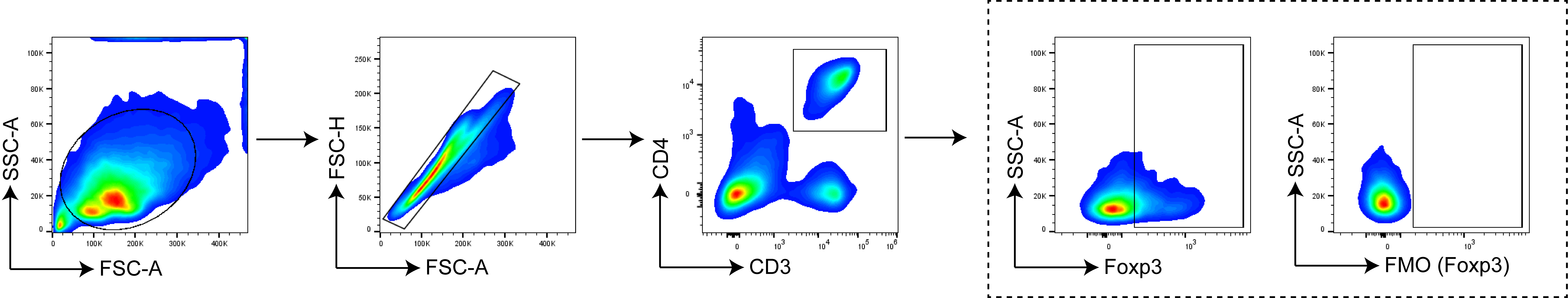


**Fig. S42** Gating strategy and fluorescence minus one (FMO) control for the flow cytometry analysis of spleen CD4^+^Foxp3^+^ Tregs (cf. **Fig. S41**).


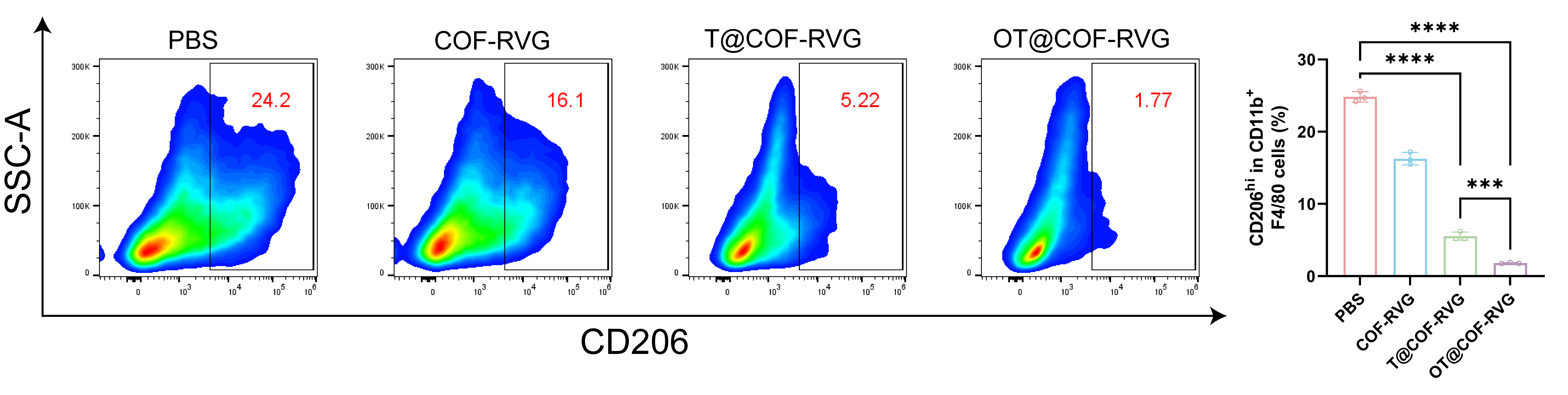


**Fig. S43** Flow cytometric assay of tumor-infiltrating M-2 like macrophages (CD206^hi^CD11b^+^ F4/80^+^) (n = 3 mice).Statistical significance was calculated through one-way ANOVA using a Tukey post-hoc test.


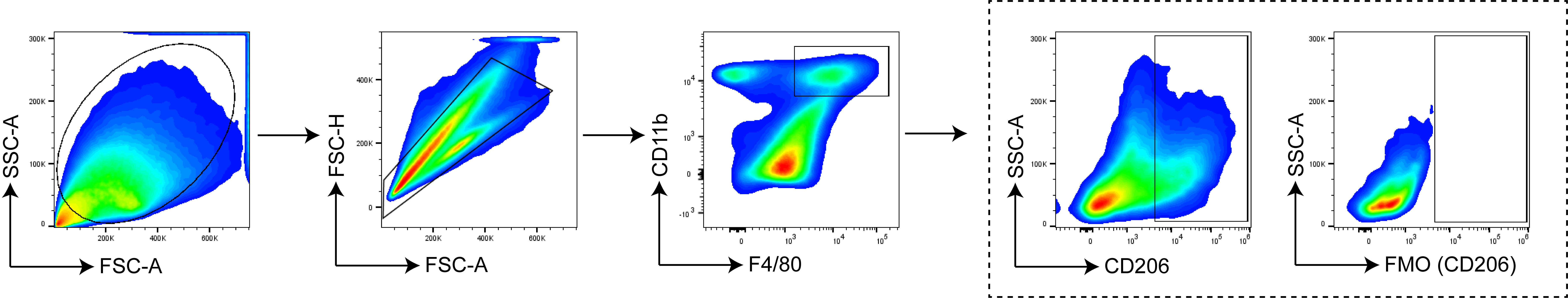


**Fig. S44** Gating strategy and fluorescence minus one (FMO) control for the flow cytometry analysis of M2-like macrophages (CD206^hi^CD11b^+^F4/80^+^) in tumor (cf. **Fig. S43**).


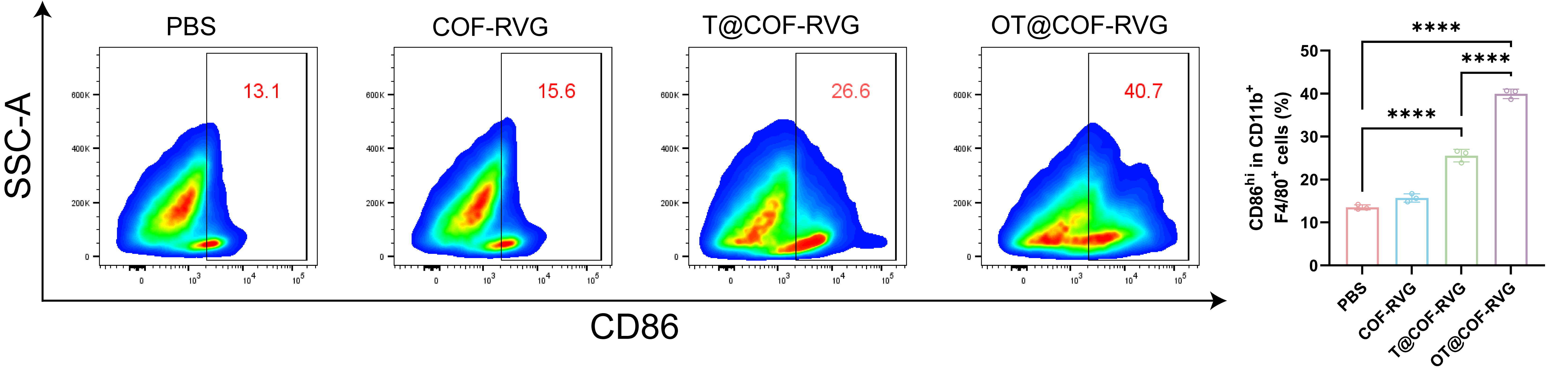


**Fig. S45** Flow cytometric assay of tumor-infiltrating M-1 like macrophages (CD86^hi^CD11b^+^ F4/80^+^) (n = 3 mice). Statistical significance was calculated through one-way ANOVA using a Tukey post-hoc test.


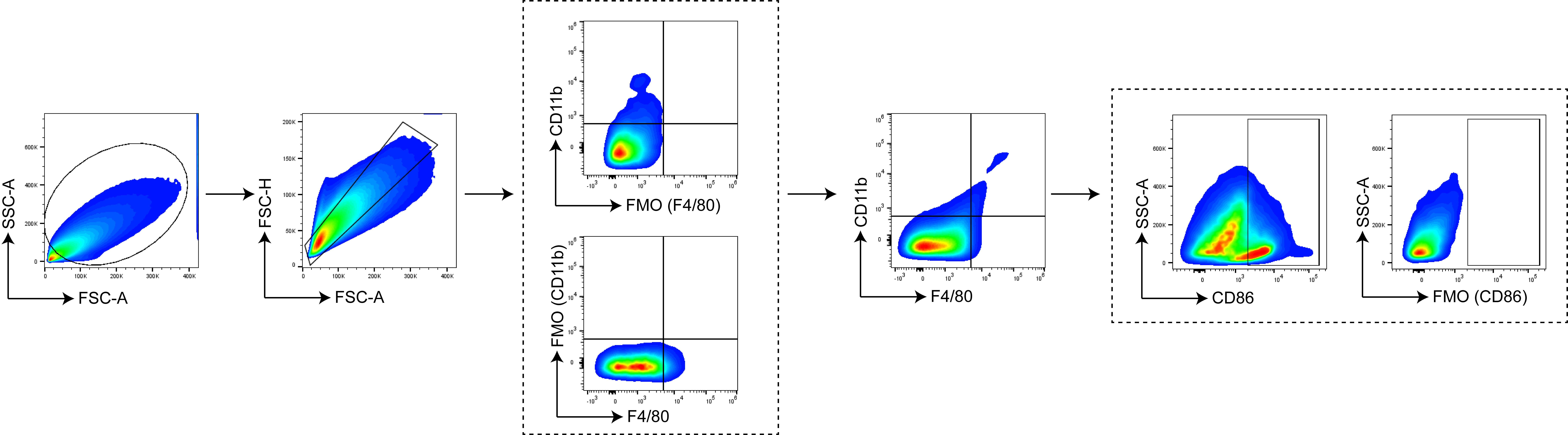


**Fig. S46** Gating strategy and fluorescence minus one (FMO) control for the flow cytometry analysis of M1-like macrophages (CD86^hi^CD11b^+^F4/80^+^) in tumor (cf. **Fig S45**)


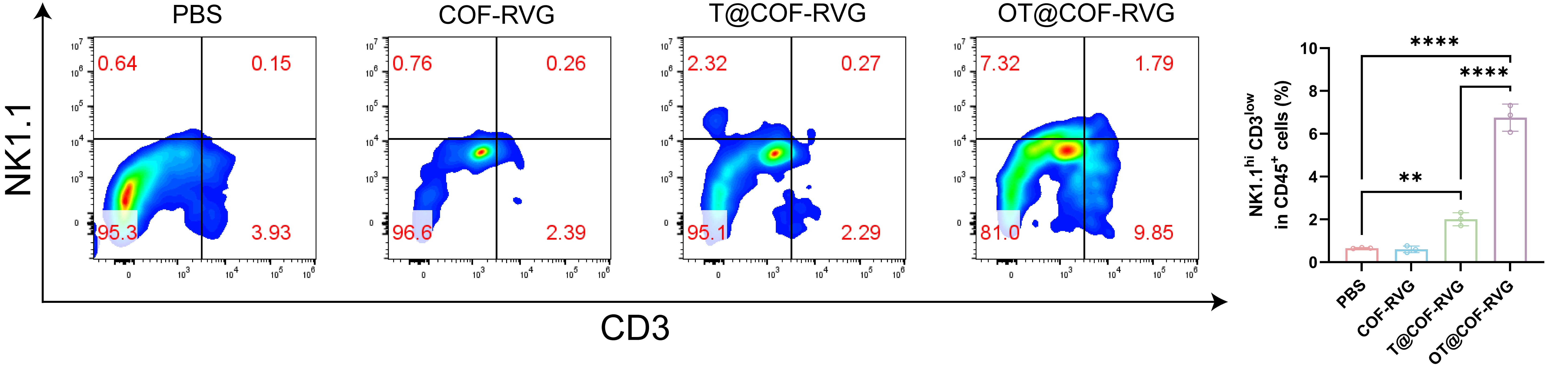


**Fig. S47** Flow cytometric assay of tumor-infiltrating NK cells (NK1.1^+^CD3^-^CD45^+^) (n = 3 mice). Statistical significance was calculated through one-way ANOVA using a Tukey post-hoc test.


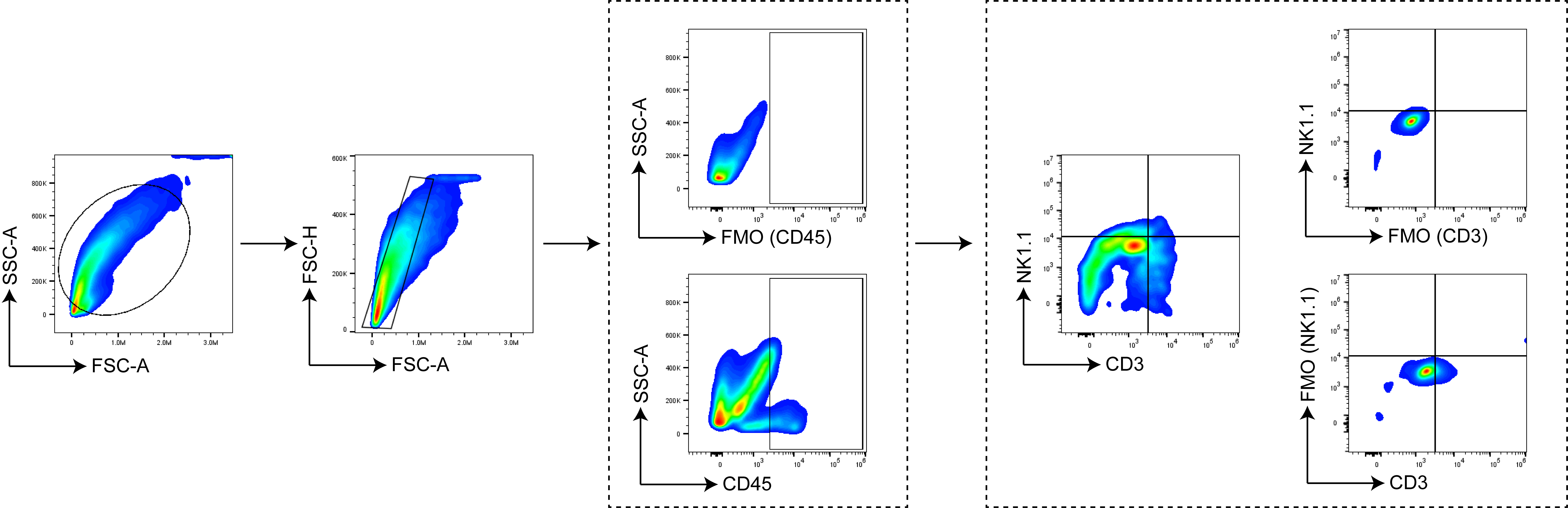


**Fig. S48** Gating strategy and fluorescence minus one (FMO) control for the flow cytometry analysis of NK cells (NK1.1^+^CD3^-^CD45^+^) in tumor (cf. **Fig. S47**).


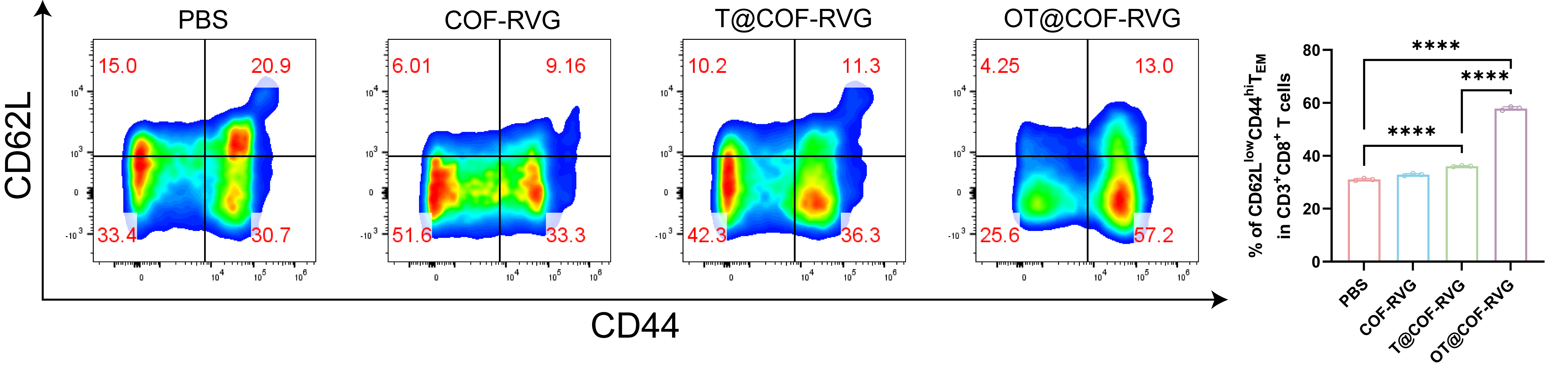
 **Fig. S49** Flow cytometric assay of T_EM_ (CD3^+^ CD8^+^ CD62L^low^CD44^hi^) in spleen (n = 3 mice). Statistical significance was calculated through one-way ANOVA using a Tukey post-hoc test.


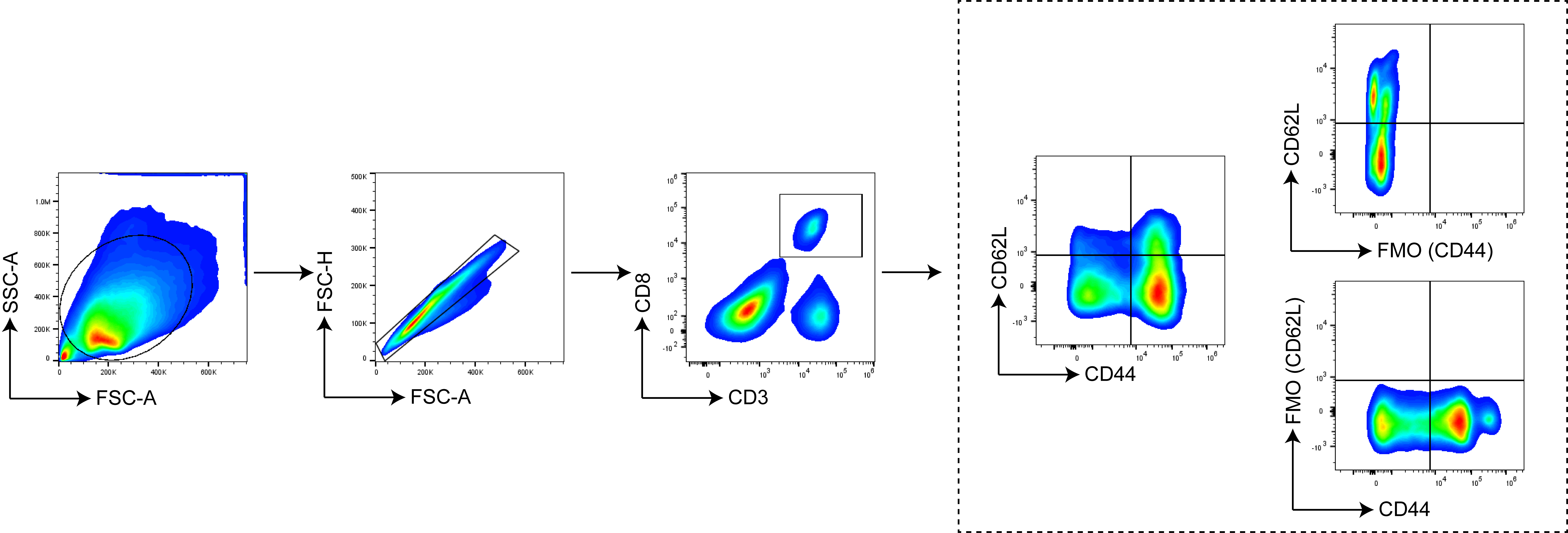


**Fig. S50** Gating strategy and fluorescence minus one (FMO) control for the flow cytometry analysis of T_EM_ (CD3^+^CD8^+^CD62L^low^CD44^hi^) in spleen (cf. **Fig. S49**).


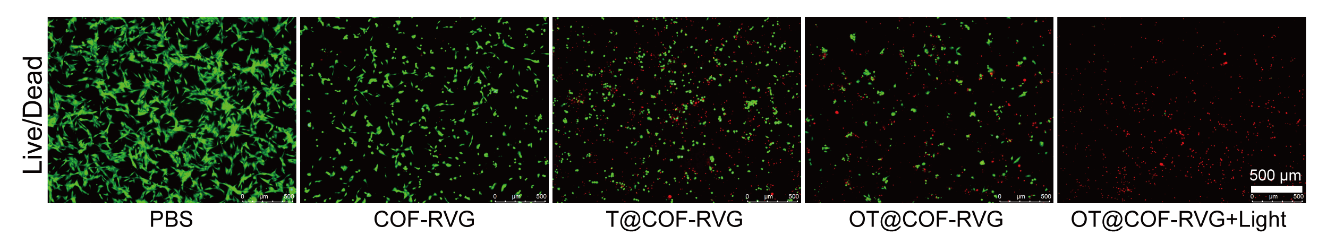
 **Fig. S51** **Live-dead cell assay.** Calcein AM/PI stain was performed to detect the survival of U87MG cells after co-incubation of U87MG and T cells with the addition of different NPs. Live cells were stained with Calcein AM (green), while dead cells were stained with PI (red).

**References**
